# How to set up and run a Bad Bugs Bookclub group

**DOI:** 10.1099/acmi.0.000846.v3

**Published:** 2024-09-06

**Authors:** Joanna Verran

**Affiliations:** 1 Department of Life Sciences, Faculty of Science and Engineering, Manchester Metropolitan University, Manchester, M1 5GD, UK

**Keywords:** bookclub, microbial literacy, public engagement, education

## Abstract

The Bad Bugs Bookclub is a public engagement initiative that enables scientists (microbiologists) and non-scientists to discuss the role of infectious disease and microorganisms in novels of fiction. The bookclub began in 2009, but since 2020, the meetings have taken place online, enabling international membership and occasional author participation. The bookclub has been shown, through peer-reviewed publications, to have impact and value to its members. For each book (the number now exceeds 100), a reading guide (questions to provoke discussion) and a meeting report (narrative of the discussion) were produced. Previously hosted on a website, the reading guides from this rich archive and resource are now presented alongside this paper, which provides tips on how to run a similar reading group.

## Data Summary

Reading guides (questions to stimulate bookclub discussion) for all books read by the Bad Bugs Bookclub between 2009 and 2023 are provided as Supplementary information (available in the online version of this article).

## Introduction

**Fig. 1.**
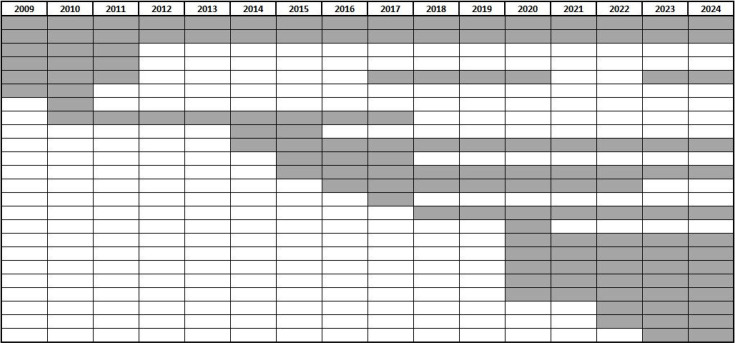
Approximate membership number of the Bad Bugs Bookclub across the 15 years of its existence (2009–2024) Each row represents an individual member. Bookclub leader not included. Data from members’ own estimation of the years encompassing meetings they attended from a 2019 survey [3] and from 2020 online registrations. There were three respondents (2019) who attended only one meeting who have not been included in the chart. Other occasional attendees have not been documented.

A bookclub, or reading group, comprises a small group of people meeting together to discuss a book they have read, usually on a regular basis [1,2]. Some bookclubs have a specific focus, on for example a particular genre of literature. There have also been bookclubs set up for science education [3,6], or within professional settings [7,8]. No group comparable to the Bad Bugs Bookclub could be found. The Bad Bugs Bookclub was set up in 2009 as a public engagement initiative, with the aim of enabling scientists (microbiologists) and non-scientists to discuss novels of fiction in which infectious disease forms part of the plot, in an informal, evening event (based in Manchester, UK). The aim was to enable two-way engagement focusing on the novel, where everyone brought something to the discussion – whether it be about the storyline, writing style, or the disease. This format provided a learning opportunity for all bookclub members, and enhanced science literacy as well as science communication and community engagement. Typically, up to eight members participated in meetings, all living in Manchester, and with around half being scientists. In 2020, the coronavirus pandemic forced meetings online. This delivery shift enabled membership to increase to around fifteen, with members joining from across the UK (Leicester, Newcastle, London), international representation (USA, Germany, Netherlands), and with the scientist: non-scientist balance remaining equal. This larger group (Fig. 1) remained loyal and enthusiastic, and meetings continued online (with occasional face-to-face social events for locally-based members). The meetings have always been led by the bookclub originator (the author of this paper), who also devises the discussion questions. The choice of book is made by the group at the previous meeting, or by email: all members are invited to suggest future books.

The longevity of the bookclub gives it credibility as a successful and engaging activity, with impact and value for its members. New findings – that the bookclub remained successful as an online activity during the pandemic, and that more non-scientists felt empowered to join – have enhanced recommendation of activities for different audiences: undergraduate and schools education [9,10] and community engagement [11], even during the COVID pandemic lockdowns [11]. Meetings can be one-off for a special event, hosted as a series, or designed as public engagement events to complement other activities.

This short communication therefore provides information to support others who might wish to set up their own Bad Bugs Bookclub, as an activity which has been demonstrably successful in public engagement, science communication, microbial literacy and education. (References [9,11] provide more detailed information on the impact and potential audiences of the Bad Bugs Bookclub, with additional background literature cited, whereas this paper is a ‘how-to’ guide).

## Procedures

### Setting up a bookclub group

Table 1 provides guidance for setting up a bookclub. At every meeting, whether held online or face-to-face, meetings begin with individual introductions, led by the bookgroup leader. It is important that the bookclub is open to new members, although the group needs to be small (8 [face-to-face] – 15 [online]) to ensure discussion is accessible to all [10,11]. Indeed, on one occasion, two meetings were run on consecutive nights, discussing the same book, to ensure that all members were able to express their views.

**Table 1.** Procedures for setting up a Bad Bugs Bookclub group. Necessary actions are listed, with suggestions made for consideration

Action	Considerations/suggestions
What is your aim	Community engagementScience/microbial literacyStudent educationFormative/summative assessmentDo you need ethical approval?
Who is your audience	Segment of the publicStudent year/level
Meeting duration	Suggest 1.5 h in a social environment to enable discussion without becoming tiring.For students, fitting in with timetables and workload.
Meeting frequency	Suggest six meetings per year (gives opportunity for other leisure reading).Secondary school perhaps three books per academic year.
First book selection	Accessibility, cost, readability, brevity, pathogen, relevance to aims (e.g. Nemesis by Philip Roth)
Call for participants	Academic mailing listCommunity groupFacebook groupUniversity/college external relations
Set up contact list	Contact each member separately if in public arena
Allow time to read the first book before identifying date	Suggest 2 months
Specify meeting location	Relate to aims and audience. Classroom, meeting room, coffee bar, bar serving alcohol, restaurant (e.g. The Island by Victoria Hislop), natural history museum (e.g. Fever 1793 by Laurie Halse Anderson).Online.
Send a reminder a few days before the event	Email (group or individual as appropriate)

Face-to-face meetings do not begin particularly promptly (late arrivals), require more ‘leadership’ to focus everyone on the ongoing discussion, and make the choice of the next book difficult (because there is no time for participants to think about preferences), but do enable the design and delivery of accompanying complementary public engagement events (for example, film screening, guided walks, special guest invitations, exhibitions) held on particular dates (for example science festivals, or special days in the microbiology calendar such as World AIDS Day, World Tuberculosis Day etc.). The leader also needs to ensure that discussion is kept on track, and to time, and that all members have had the opportunity to speak if they wish (confirmed via individual follow-up emails).

Until 2020, face-to-face meetings were the norm for the Bad Bugs Bookclub, but the pandemic forced meetings online [11]. It is easier to lead online meetings (Zoom has been preferable to Teams, for wider and easier access for all members: Teams was used once, with varying success) than face-to-face-although significant preparation is required whatever the format. Online, there are no distractions, and all members are engaged in the discussion (One benefit of the pandemic).

Online meetings also facilitated attendance by members at distance, increased membership numbers of both scientists and non-scientists (easier to attend), enabled occasional author attendance, ensured a prompt start and inspired more committed discussion and engagement, perhaps as various lockdowns engendered more of a community spirit. Suggestions for the next online reading are made through email, with members selecting preferences over a week. The majority vote determines the next read.

Since 2020, bookclub meetings have remained online, with occasional face-to-face meetings on a different topic for local members. (These topics are generally more broad, focusing on for example – non-fiction; books about COVID; favourite reads/new recommendations; apocalyptic survival stories).

Although it is not desirable to have too large a group, membership remains open. New members hear about the bookclub through professional societies, social media, authors etc.: they then contact the group leader through email, on average one enquiry per month.

### Using the reading guides

Reading guides for books read by the group from 2009 to 2023 are presented as supplementary information. For each book, there is a series of questions that help to guide discussion. The bookclub leader needs to do preparatory reading for extension of discussion, such as relating to current concerns (for example vaccination, AMR, emerging pathogens, current outbreaks and any other stories relevant to the pathogen of concern), and might need or like to modify the suggested questions. Meetings tend to follow a particular format, based on the reading guide, where the merits of the book as a ‘good read’ are considered before talking about the pathogen described. Using the narrative, discussion can progress into disease epidemiology and the scientific accuracy of the storyline, misconceptions and real-life comparisons, and consideration is made of the book as a potential educational resource for other audiences (such as students).

The reading guides are presented in chronological order (ie the order in which the meetings were held). The book titles, author and date of publication are listed as contents at the beginning of the document, with page numbers added that allude to the questions for each book. Perusal of the reading guides reveals changes in format to a more consistent form, and a more nuanced perspective of the literature. Since the reading guides were prepared in the past, there is of course opportunity for updating – for example to encompass the pandemic, new outbreaks or discoveries, or other newsworthy stories. Table 2 comprises a list of books that have provoked good discussion, and that might be useful starters for any new reading groups.

**Table 2.** Suggested (personal recommendations) ‘good’ books for Bad Bugs Bookclub discussions with brief note on context encompassing range of topics. The list is essentially in order of preference (Nemesis) or date of bookclub meeting. The last three are selected representatives of books about diseases (influenza, HIV/AIDS, COVID) where several are available

Book title	Reason for recommendation
Nemesis by Philip Roth (1971)	Short, easy to read, excellent account of polio
I am Legend by Richard Matheson (1954)*	Short, zombie/vampires, scientific method
The Waiting Rooms by Eve Smith (2020)	AMR, contemporary setting, provocative issues
The Island by Victoria Hislop (2005)†	East to ready, time-spanning, leprosy and cure
Arrowsmith by Sinclair Lewis (1924)‡	Ethics, clinical trials, medical profession (US), history
The Ghostmap by Steven Johnson (2006)§	Non-fiction, cholera, epidemiology, history
Oryx and Crake by Margaret Atwood (2003)	Detailed, technology, ethics, apocalypse
Aurora by Kim Stanley Robinson (2015)	Space travel, ecology, prions
The Death of Grass by John Christopher (1956)	Short, plant pathogen, journey, slightly dated
The Last Days of Smallpox by Mark Pallen (2018)	Non-fiction, investigative, academia
State of Wonder by Anne Patchett (2011)	Evocative, dream-like, malaria, pharma, scientists
The Butchering Art by Lindsey Fitzharris (2017)¶	Non-fiction, Lister biography, fundamental microbiology
How to Survive a Plague by David France (2016)**	Non-fiction, HIV/AIDS, activism, treatment
Not Forgetting the Whale by John Ironmonger (2015)††	Influenza, isolated communities, community spirit
Many Kinds of Love by Michael Rosen (2021)‡‡	Non-fiction, COVID, Long COVID, some poetry, NHS

1. *There are many ‘zombie’ novels: other good meetings focused on World War Z by Max Brooks (2006) and The Girl with All the Gifts by MR Carey (2014).

2. †Other excellent ‘leprosy’ novels: Moloka’i by Alan Brennert (2003) and Frog Music by Emma Donoghue (2014).

3. ‡The Citadel by AJ Cronin (1937) gives a UK slant on the medical profession pre-NHS.

4. §Or The Great Stink by Clare Clark (2005): fiction.

5. ¶Several accounts have been published about pioneering microbiologists. Microbe Hunters by Paul de Kruif was the first (1926).

6. **There are many novels about HIV/AIDS that the Bookclub has read – and many we have not. This book is a fantastic overarching narrative that has been recommended as supplementary reading for our group.

7. ††Just one of many novels about influenza: in each, the infectivity, antigenicity and epidemiology of the emerging strain can be explored.

8. ‡‡One of the first books to be published during the pandemic. Subsequently many are available, both fiction and non-fiction. The epidemiology of COVID was explored through many other books read during the pandemic.

## Conclusions

The Bad Bugs Bookclub format provides an opportunity for scientists to engage with a range of different audiences [10] . The Bad Bugs Bookclub has discussed more than 100 novels from a range of genres, encompassing a diversity of pathogens (including zombies [12], pandemics and post-apocalyptic scenarios. The reading guides presented as supplementary information in this paper enables the selection of books that can be used to address infectious diseases of current concern as well as those of historic importance. Discussion about these novels can reveal misunderstandings about scientific concepts, enable connections to be made between past and current outbreaks, and raise awareness of key preventative and curative measures. Loyalty of the Bad Bugs Bookclub membership (particularly online) has resulted in a committed social group, separated by geography. The informality of the bookclub meetings means that science literacy is used and enhanced by the lightest of touch. The bookclub format provides a rewarding means of enhancing science communication, microbiology education and public engagement.

## supplementary material

10.1099/acmi.0.000846.v3 Uncited Supplementary Material 1.
