# Supplementary material for: How to set up and run a Bad Bugs Bookclub group
Source: Access Microbiol. 2024 Sep 6;6(9):000846.v3. doi: 10.1099/acmi.0.000846.v3 (PMC11379038; doi:10.1099/acmi.0.000846.v3)
Supplement: Uncited Supplementary Material 1. [file acmi-6-00846-s001.pdf]

2009-2023

The Bad Bugs Bookclub

Reading Guides

Joanna Verran
1-1-2024

The Bad Bugs Bookclub Resource

This document contains reading guides (questions for discussion) for all books read by the Bad Bugs Bookclub from 2009 – 2023, almost 100 novels, primarily fiction, but some non-fiction. The guides were originally posted on the Bookclub website, which went offline in March 2023. (N/A = not sufficient relevant content)

The books are listed in the order in which they were discussed. The reading guides were prepared before each meeting, but were modified post-meeting for clarification purposes and in the light of discussion that took place. The questions posed might address issues that were topical at the time of the meeting, particularly COVID, rather than the present: of course, questions can be altered/updated.

Each reading guide begins with the same text, to introduce the reader to the bookclub, followed by a note (of varying length) about the book being discussed, thus individual book reports can be downloaded independently of others if desired. There is some change in format as the meetings progressed.

Index

Meeting	Title	Author	Publication	Page
2009	The Hot Zone	Richard Preston	2012	7
	Year of Wonders	Geraldine Brooks	2001	9
	Dorian	Will Self	2002	11
2010	The Andromeda Strain	Michael Crichton	1969	13
	Mary Barton	Elizabeth Gaskell	1848	15
	The Calcutta Chromosome	Amitav Ghosh	1995	17
	Unnatural Exposure	Patricia Cornwell	1997	19
	The Body Farm	Patricia Cornwell	1994	22
	Dracula	Bram Stoker	1897	24
2011	The Satan Bug	Alistair Maclean	1962	26
	The Street Philosopher	Matthew Plampin	2009	28
	Star of the Sea	Joseph O’Connor	2002	31

	Toxin	Robin Cook	1998	31
	The Immortal Life of Henrietta Lacks	Rebecca Skloots	2010	35
	I am Legend	Richard Matheson	1954	37
	World War Z	Max Brooks	2006	39
	28 Stories of AIDS in Africa	Stefanie Nolen	2007	41
2012	The Island	Victoria Hislop	2005	43
	Nemesis	Philip Roth	1971	45
	The Painted Veil	W Somerset Maugham	1925	47
	Intuition	Allegra Goodman	2006	49
	Arrowsmith	Sinclair Lewis	1924	52
2013	The Air We Breathe	Andrea Barrett	2007	54
	The Ghostmap	Steven Johnson	2006	56
	The Strain	Guillermo del Toro And Chuck Hogan	2009	N/A
	A Wind in the Door	Madeline L'Engle	1973	58
	Fever	Mary Beth Keane	2013	60
	Microbe Hunters	Paul de Kruif	1926	62
2014	The Last Town on Earth	Thomas Mullen	2006	64
	Petroplague	Amy Rogers	2011	66
	Rant	Chuck Palahniuk	2007	N/A
	Code Orange	Caroline Cooney	2005	68
	The First Eagle	Thomas Hillerman	1998	70
	Oryx and Crake	Margaret Atwood	2003	72

2015	Annihilation	Jeff van der Meer	2014	74
	Parasite	Mira Grant	2013	76
	The Lazarus Strain	Ken McClure	2007	78
	Fever Medicine	Shawn Harmon	2013	80
	The Trouble with Lichen	John Wyndham	1960	82
	Second Avenue Caper	Joyce Brabner	2014	84
2016	Consumed	David Kronenberg	2014	N/A
	The Plague	Albert Camus	1947	86
	The Girl with All the Gifts	M R Carey	2014	88
	A Fierce Radiance	Lauren Belfer	2010	90
	The Manchester Man	Isabella Banks	1876	92
2017	A Journal of the Plague<br>Year	Daniel Defoe	1722	94
	The Pesthouse	Jim Crace	2007	96
	A Lovely way to Burn	Louise Welsh	2014	98
	Isolation	S J Gardiner	2016	98
	I Contain Multitudes	Ed Jong	2015	101
	A Prayer for the Dying	Stewart O’Nan	1999	103
2018	The Stand	Stephen King	1990	105
	The Citadel	A J Cronin	1937	107
	Frog Music	Emma Donoghue	2014	109

	The Last Days of Smallpox	Mark Pallen	2018	111
	The Last Man	Mary Shelley	1826	113
	Aurora	Kim Stanley Robinson	2015	116
2019	The Samurai's Garden	Gail Tsukiyama	1994	118
	The Death of Grass	John Christopher	1956	121
	Station Eleven	Emily St John Mandel	2014	123
	The Killing Snows	Charles Egan	2012	125
	The Health of Strangers	Lesley Kelly	2017	127
2020	Contagion	Robin Cook	1995	130
	Fever 1793	Laurie Halse Andersen	2000	133
	Arthur Mervyn	Charles Brockden Brown	1799	133
	An American Plague	Jim Murphy	2008	133
	Pale Rider	Laura Spinney	2017	136
	Pale Horse, Pale Rider	Katharine Ann Porter	1937	136
	The Eyes of Darkness	Dean Kootz	1981	136
	Not Forgetting the Whale	John Ironmonger	2015	139
	The Constant Gardener	John Le Carre	2001	141
	The Waiting Rooms	Eve Smith	2020	144
2021	Little Women	Louisa May Alcott	1868	148
	The Bone Garden	Tess Gerritsen	2007	148
	Moloka'i	Alan Brennert	2003	151

	The Pull of the Stars	Emma Donoghue	2020	153
	Many Kinds of Love	Michael Rosen	2021	155
	Summer	Ali Smith	2020	155
	Hamnet	Maggie O'Farrell	2020	157
	The Perfect Predator	Steffanie Strathdee and Thomas Patterson, 2019	2019	159
2022	The End of Men	Christina Sweeney Baird	2020	162
	Darwin's Radio	Greg Bear	1999	164
	Off Target	Eve Smith	2022	166
	The Bone Code	Kathy Reichs	2021	169
	The Locked Room	Ellie Griffiths	2022	169
	The Fever Tree	Jennifer McVeigh	2012	172
	State of Wonder	Ann Patchett	2011	174
	Love from the Pink Palace	Jill Nader	2022	176
	All the Young Men	Ruth Coker Burks	2020	176
2023	Pure	Andrew Miller	2011	177
	The Great Stink	Clare Clark	2005	181
	Doomsday Book	Connie Willis	1992	183
	Spike	Jeremy Farrar and Anjana Ahuja	2021	185
	The Butchering Art	Lindsey Fitzharris	2017	187
	The Great Believers	Rebecca Makkai	2018	189
	The Way We Live Now	Susan Sontag	1986	189

Bad Bugs Book Club Reading Guide: The Hot Zone, by Richard Preston

The aim of the Bad Bugs Book Club is to get people interested in science, specifically microbiology, by reading books (novels) in which infectious disease forms some part of the story. We also try to associate books, where possible, with some other activity or event, to widen interest, and to broaden impact.

We hope to encourage others to join, to set up their own bookclub, suggest books and accompanying activities to us, and give feedback about the books that they have read (j.verran@mmu.ac.uk).

Our bookclub comprises both microbiologists and members of the general public. We felt that this would encourage some discussion on the science – accuracy, impact etc – as well as about the book.

The Hot Zone is described on its jacket as ‘one of the most terrifying books I’ve ever read’ (Arthur C Clarke). It describes the author’s search for the story of the emergence of Ebola virus, and the story itself, through his interviews with key players. It includes several graphic descriptions of symptoms, and some detailed scientific narratives.

We coupled the book club reading event with a prior screening of the film ‘Outbreak’, starring Dustin Hoffman. The film depicts the emergence of Ebola in scenarios comparable to the book, although occasionally using rather more artistic license in some of the scientific aspects.

Some points for discussion are suggested below.

Discussion points:

Presentation:

Did you enjoy the book/film?

How did the film compare with the book in terms of similarities and difference of content?

How were the different personalities presented in the film and the book? Were they the same people? Were there similar characters?

Did you prefer the book or the film?

Which was more inspiring scientifically?

Which bits did you enjoy most?

Content:

Was the science interesting? Surprising? Accurate?

What were the implications of the emerging infection to the wider global population?

How topical is the content?

Before reading this book, had you come across much information on infectious disease? If so, how did the book compare with your prior knowledge? Was it contradictory/confirmatory?

Has reading the booked had any impact on your thoughts around infectious diseases and how they are controlled?

Joanna Verran

Bad Bugs Book Club Reading Guide: Year of Wonders by Geraldine Brooks

The aim of the Bad Bugs Book Club is to get people interested in science, specifically microbiology, by reading books (novels) in which infectious disease forms some part of the story. We also try to associate books, where possible, with some other activity or event, to widen interest, and to broaden impact.

We hope to encourage others to join, to set up their own bookclub, suggest books and accompanying activities to us, and give feedback about the books that they have read (j.verran@mmu.ac.uk).

Our bookclub comprises both microbiologists and members of the general public. We felt that this would encourage some discussion on the science – accuracy, impact etc – as well as about the book.

Year of Wonders is a narrative of the outbreak of plague that occurred in 1665 in Eyam, a small Derbyshire village. The village isolated itself from neighbouring communities to prevent spread of the disease, and many villagers died. We coupled this bookclub meeting with a visit to the village itself. For bookclubs that are less conveniently close to Eyam, there are many factual books and articles, and other novels (Pepys, Defoe) about the events, a musical (Eyam!), a play (the Roses of Eyam), school learning guides etc.

Some points for discussion are suggested below.

Discussion points:

The book – did you enjoy it?

Based on a well-known story? (was it known to you?)

Likes and dislikes? Language, vocabulary?

Bringing in lots of issues that reflected the time (witchcraft, healing, superstition, lead mining, non-conformism, poppies)?

Useful for science-literature relationship?

Characters

Were the characters real and believable?

Do they change or evolve through the course of the story?

Plot

Can you relate to the predicament?

Comments on content?

Anything you liked/didn't like?

Visit

Did the visit affect your perception of the story? If yes, in what way?

Differences between the real story and the novel – does it matter?

Sentimentalisation of the story 150 years on et seq; heroic tales helping the legend?

Impact on current consciousness of village – tourism, education?

The Science

The Eyam plague was real.

Relationship to current infectious disease?

Value of isolation - heroic? lack of choice (related to wealth – nowhere to go)? Worsened situation? Imposed from outside (cordon sanitaire)?

Was it really plague? (rats, fleas)

Immunity to plague related to HIV?

The plague nowadays?

Other historical plague associations (Pepys, Defoe, etc)?

Joanna Verran

Bad Bugs Bookclub Reading Guide: Dorian by Will Self

The aim of the Bad Bugs Book Club is to get people interested in science, specifically microbiology, by reading books (novels) in which infectious disease forms some part of the story. We also try to associate books, where possible, with some other activity or event, to widen interest, and to broaden impact.

We hope to encourage others to join, to set up their own bookclub, suggest books and accompanying activities to us, and give feedback about the books that they have read (j.verran@mmu.ac.uk).

Our bookclub comprises both microbiologists and members of the general public. We felt that this would encourage some discussion on the science – accuracy, impact etc – as well as about the book.

Dorian describes events around the beginning of the HIV pandemic, when cases appeared to predominate amongst homosexuals. The book parallels Oscar Wilde's novel, The Picture of Dorian Gray, in that Dorian's deterioration is hidden from his contemporaries (in effect HIV positive status) whilst a hidden video installation reveals the damage done by disease and corruption.

Some points for discussion are suggested below.

Discussion points:

The book

Did you enjoy it? Language? Content? First paragraph? Imagery?

Do you know anything about Oscar Wilde's original novel, The Picture of Dorian Gray?

How well did Dorian parallel Oscar Wilde's original? Is it a clever idea?

(have you read it? Does it matter if you did or not?).

In what ways was it similar/different to the original?

Useful for HIV/AIDS education? In what way (if yes, or no)

Regarding HIV infection and AIDS, were any parts informative?

Shocking?

Characters

Some of the characters were also in the original novel.

How did you feel about the characters? Did you like/dislike anyone particular?

Were they real and believable?

Did they change or evolve through the story?

Plot

The plot uses HIV and AIDS as a (rather obvious?) parallel between the deterioration of the picture of Dorian Gray. In this case, it is a video installation rather than a portrait.

Were you able to follow the plot?

There is one incident which affects all the key characters?

The epilogue?

Science and cultural events

The bookclub meeting was held on World AIDS Day. Does the message from the book inform about HIV/AIDS today?

Information on symptoms, treatment etc are provided – has the situation altered since the book was written (and since the setting of the novel?)?

Joanna Verran

Bad Bugs Bookclub Reading Guide: The Andromeda Strain by Michael Crichton

The aim of the Bad Bugs Book Club is to get people interested in science, specifically microbiology, by reading books (novels) in which infectious disease forms some part of the story. We also try to associate books, where possible, with some other activity or event, to widen interest, and to broaden impact.

We hope to encourage others to join, to set up their own bookclub, suggest books and accompanying activities to us, and give feedback about the books that they have read (j.verran@mmu.ac.uk).

Our bookclub comprises both microbiologists and members of the general public. We felt that this would encourage some discussion on the science – accuracy, impact etc – as well as about the book.

The Andromeda Strain, written in 1969, describes an outbreak of a lethal and highly infectious disease brought to earth by a satellite, launched by the US Government to explore microorganisms in space (potentially for germ warfare). Death occurs within three seconds, and a group of scientists are brought together to identify the cause of the infection, and to find a cure. The book focuses on the actions of these scientists and their progress.

Some points for discussion are suggested below.

Discussion points:

The book

Did you enjoy the book? Have you read any other books/do you know of any films of books by Michael Crichton?

Did you like the characters?

Do you think the plot is unlikely, or can you see aspects that might be relevant to today? Did you spot any aspects of the plot that seemed erroneous, or that distracted you?

There is a lot of description of technology and equipment. Did you find this interesting? Do you think that it is correct? Can you find any items that are in common use today?

Did you think that the science described was accurate? Do you need to believe in the science of the novel, or does it not matter to you?

Were any aspects of the science particularly interesting? Surprising?

Microbiology forms a key part of the plot. It is one of the aims of the Bad Bugs Bookclub to encourage discussion about microbiology between scientists and the general public. Can you identify discussion points?

Do you see a value in the novel for public engagement/discussion regarding microbiology/science?

Film portrayals

Did you see the film (1972)? The television mini-series (2008)?

How did the film compare with the book in terms of similarities and difference of content?

How were the different personalities presented in the film and the book? Were they the same people? Were there similar characters?

Did you prefer the book or the film?

Which was more inspiring scientifically?

Which bits did you enjoy most?

Joanna Verran

Bad Bugs Book Club Reading Guide: Mary Barton by Elizabeth Gaskell

The aim of the Bad Bugs Book Club is to get people interested in science, specifically microbiology, by reading books (novels) in which infectious disease forms some part of the story. We also try to associate books, where possible, with some other activity or event, to widen interest, and to broaden impact.

We hope to encourage others to join, to set up their own bookclub, suggest books and accompanying activities to us, and give feedback about the books that they have read (j.verran@mmu.ac.uk).

Our bookclub comprises both microbiologists and members of the general public. We felt that this would encourage some discussion on the science – accuracy, impact etc – as well as about the book.

This novel was selected for the Bad Bugs Bookclub for a number of reasons: being set in Manchester, it provided an opportunity for a guided walk around the city, enabling us to visit some of the locations mentioned, to learn about the living conditions of the poor, to see any remains of the industrial heritage, and to think about public health in the nineteenth century in the world's first industrial city. This year, 2010, marks the bi-centenary of Mrs Gaskell's birth, and the Gaskell Society is active in Manchester, where she lived for part of her life.

This is the first 'classic' nineteenth century novel that the bookclub has tackled. The role of microbiology within the plot is very much intertwined with the general living conditions of the poor of the time, thus needs picking out if discussion is intended. There is opportunity for additional reading, investigating the conditions in Manchester of that time, implications for public health, research into the diseases mentioned – their transmission, prevalence, prevention and treatment – as well as those diseases which are not mentioned. Many other novels set in the nineteenth century could be used for the same discussions, for example:

Little Women by Louisa M Alcott, describes Beth's struggle with scarlet fever – this disease was a major killer of women in childbirth, and children in the 1800s.

Any novel by Charles Dickens will address similar aspects of poverty and disease.

The Manchester Man by Mrs Linnaeus Banks, was published in 1876, but set earlier than Mary Barton. This novel describes the life and times of Jabez Clegg, incorporating the Peterloo Massacre, again setting in some detail many of the

historical sites of Manchester, some of which, as for Mary Barton, remain, and some which do not.

Questions

1.Did you enjoy the novel? The language? The inclusion of footnotes and side comments made by the author?

2. The storyline begins with full accounts of the living conditions of the working poor in Manchester, but shifts later to a different sort of novel. Were you comfortable with the change?

2. Did you like the characters? Did you feel that they developed through the novel?

3. The key characters live in poverty. Did you feel that living conditions were well described? How did the characters cope with their lot?

4. The first few pages mention several different illnesses. How is the general health of the population portrayed?

4. At that time, many diseases were prevalent, with little access to any form of treatment. How did the characters cope with disease? What different diseases were mentioned or described? How were they transmitted? How were diseases treated?

5. Both Manchester and Liverpool figure in the novel. How are the two cities portrayed?

Joanna Verran

Bad Bugs Book Club Reading Guide: The Calcutta Chromosome by Amitav Ghosh

The aim of the Bad Bugs Book Club is to get people interested in science, specifically microbiology, by reading books (novels) in which infectious disease forms some part of the story. We also try to associate books, where possible, with some other activity or event, to widen interest, and to broaden impact.

We hope to encourage others to join, to set up their own bookclub, suggest books and accompanying activities to us, and give feedback about the books that they have read (j.verran@mmu.ac.uk).

Our bookclub comprises both microbiologists and members of the general public. We felt that this would encourage some discussion on the science – accuracy, impact etc – as well as about the book.

The reason we chose this book was that it had malaria as a focus within the narrative, and we were having the meeting on World Malaria Day (25th April 2010). The plot develops around the work of scientist Ronald Ross, who made the discovery that mosquitoes transmit malaria – for which he received the Nobel Prize in 1902. However, on top of this interesting example of the history of microbiology, and the problems, attitudes and thoughts of pioneer microbiologists of the nineteenth century, the novel adds additional fictional layers, encompassing Indian mysticism, chromosome/personality transfer, immortality, aspects of Indian culture and Colonialism.

Questions

1. Did you enjoy the novel? Did you find the plot easy to understand? What is your interpretation of the plot? What aspects did you like/dislike?

2. What about the characters? Were they well drawn? How many of them have/have had malaria? Did you see the repeated characters? Could you identify who was who? Lutchman is very intangible? Sonali/Urmila? The boy with the gap tooth?

3. Memorable images?

4. Indian mysticism? The link with microbiology?

5. The historical aspects of microbiology? Did you want to find out more about malaria?

6. What value might the novel be for encouraging discussion about malaria? Ideas for activities? The history of medicine? The global importance of malaria?

7. Colonialism? Ethics? Difficulties of pioneering research, associating a pathogen with a disease?

Joanna Verran

Bad Bugs Book Club Reading guide: Unnatural Exposure by Patricia Cornwell

The aim of the Bad Bugs Book Club is to get people interested in science, specifically microbiology, by reading books (novels) in which infectious disease forms some part of the story. We also try to associate books, where possible, with some other activity or event, to widen interest, and to broaden impact.

We hope to encourage others to join, to set up their own bookclub, suggest books and accompanying activities to us, and give feedback about the books that they have read (j.verran@mmu.ac.uk).

Our bookclub comprises both microbiologists and members of the general public. We felt that this would encourage some discussion on the science – accuracy, impact etc – as well as about the book.

Patricia Cornwell introduced Dr Kay Scarpetta in her first novel, Postmortem, written in 1990. Scarpetta is the Chief Medical Examiner, in Richmond, Virginia – although there are changes in her status though the career documented in the series. Cornwell has been a volunteer police officer, a crime reporter, and a computer analyst in the Chief Medical Examiner's office. Unnatural Exposure (1997) is the eighth Scarpetta novel.

The novels all describe various murders, with significant technical detail, particularly including recent forensic technology (but thus susceptible to dating). They also span a number of years, and the key characters develop through the novels. Unnatural Exposure begins with Scarpetta investigating a series of murders in which the victims were beheaded and dismembered. A new torso is discovered, which turns out to be a copycat killing. One of the key differences is that the victim presents some vesicular lesions reminiscent of poxvirus. Subsequently an infected body is found on Tangier Island, an isolated area with few inhabitants. Smallpox appears to have been the causative agent. Several other cases are identified, and an epidemic is feared…

The use of the internet in the novel is interesting – for the time it must have been very innovative, but unfortunately emails, attachments, chatrooms are no longer news.

Questions

1. Did you enjoy the novel? Have you read any others of the series? If not, would you now?
2. Characters – were they sympathetic/well described?
3. Plot – exciting, interesting? Any particularly exciting/dull bits?
4. Science – useful, interesting?
5. Microbiology – opportunities for identifying topics for discussion. Plenty of additional research to carry out. Smallpox remains an important pathogen, with importance in vaccine development, bioterrorism etc.

Questions for students

In terms of microbiology education, there are many points for discussion, or for students to update and research. For example, you could set different goals for a group of students on topics arising from the novel;

1. The entire story happens over winter, with Scarpetta suffering from a bad bout of flu (not smallpox). Would this be seasonal flu, or a pandemic? What year was the book set in?
2. Initially, VZV is suspected as the cause of the rash – there is some discussion about its aetiology, and of the vaccine (in US, but not in UK). What are the differences and similarities between VZV and smallpox?
3. Wingo, Scarpetta's assistant is HIV positive. What was the treatment status for HIV in 1997?
4. Scarpetta advises her chief of police to eat his burger medium rather than medium rare because of E.coli O157. How new is O157 in 1997?
5. Microscopic (light) of the tissues specimen showing the Guanieri bodies 'waves of bright red eosinphilic inclusions within infected epithelial cells, or the cytoplasmic Guanieri bodies indicative of a pox-type virus'. Opportunity for discussion on pox diagnosis via light microscopy (large virus).
6. From transmission electron microscopy a 'DNA double stranded brick shaped virus particles 200 – 250 nanometers diameter' is described (How would DNA structure be known?)
7. There are several wider issues discussed: USAMRIID – CDC, issues of terrorism, outbreaks, vaccination, quarantine. Did these work well within the plot?
8. Access to level 4 biosecurity (as in Outbreak movie and Hot Zone novel) for autopsy. 'scientists doing open war with Ebola, Hantavirus and unknown diseases for which there was no cure'. What are these security levels?
9. There is a bad economic situation affecting staffing (furlough): …'not to mention Medicaid, air pollution and tracking the winter flu epidemic or screening water supplies for the cryptosporidium parasite'…Did the list of outbreaks at that time provide additional stress to the plot?
10. How was the virus transmitted? blue crab? ..'a food, a drink, dust? With smallpox, transmission is airborne, spread by droplets or fluid from the lesions. The disease can be carried on a person or his clothes' . 'we've got to worry about houseflies hovering around patients, crabs headed for the

mainland…mosquito transmission, as in Tanapox'…what other poxviruses are there? What is whitepox?

11. Where is any residual smallpox? Should it all be destroyed? What are the arguments?
12. The different buildings in CDC are described. Why are the separate buildings needed? …bacteria and influenza labs, and the red brick and concrete area for rabies and AIDS
13. Other scientific procedures/issues are noted: PCR; mutants, difficulties of producing a vaccine (AIDS); diagnostic test. Is this technology useful for the 'Bad Bugs Bookclub' reader? For education?
14. When was smallpox declared eradicated? How was eradication achieved? What is the history of smallpox vaccination? Is lack of immunity in the population due to smallpox eradication and cessation of vaccination an issue?
15. Scarpetta expresses fear of disease as compared with other means of death. How do you feel?
16. How easy would it be to equip a lab in a trailer for cultivation of poxvirus?
17. The Birmingham (UK) outbreak is mentioned. What happened then? What were the repercussions?

Joanna Verran

Bad Bugs Bookclub Reading Guide: The Body Farm by Patricia Cornwell

The aim of the Bad Bugs Book Club is to get people interested in science, specifically microbiology, by reading books (novels) in which infectious disease forms some part of the story. We also try to associate books, where possible, with some other activity or event, to widen interest, and to broaden impact.

We hope to encourage others to join, to set up their own bookclub, suggest books and accompanying activities to us, and give feedback about the books that they have read (j.verran@mmu.ac.uk).

Our bookclub comprises both microbiologists and members of the general public. We felt that this would encourage some discussion on the science – accuracy, impact etc – as well as about the book.

The Body Farm is the fifth book about Kay Scarpetta, written by Patricia Cornwell in 1994.Within the plot, the use of a facility in Knoxville Tennessee to help solve a murder is described. This ‘Body Farm’ is an actual location where human bodies are allowed to decompose, usually in the open air, under specific conditions, to enable various parameters to be noted. The information gained can be used to help solve a range of crimes and problems relating to cause of death. The novel does not contain a significant amount of microbiology or forensic science despite its title. Information on the ‘real’ Body Farm can be found on the Internet, with film that can be used to illustrate discussion.

There are a number of aspects that can be used for discussion.

Questions

Do you typically read this type of novel? How does this novel compare with others in the series? Other authors? Which authors of this type of novel do you prefer, if any? Will you read more of this series, or similar novels?

Did you like the book? Characters? Plot? Writing style? How do you picture the characters?

If this is a ‘bad bugs bookclub’, how much microbiology is in this novel? Directly, or indirectly?

Was there sufficient forensic/scientific content to sustain interest and focus for the aims of a ‘bad bugs bookclub? Or perhaps for a bookclub focusing on forensic science?

Did you find the technology dated or current? Are there comparable technological developments currently receiving attention? Is the use of technology to identify criminals, or individuals generally, intrusive? (eg DNA databases?)

How similar is fictional forensics to real forensics? Is the relationship useful or misleading? How might students on forensic courses view the subject before applying/during study/after graduation.

Do the novel and the documentary provide a useful focus for discussion?

How valuable might this type of novel be (Kathy Reich, Patricia Cornwell, Jefferson Bass etc) in discussion relating to scientific aspects of forensic science studies?

Joanna Verran

Bad Bugs Bookclub Reading Guide: Dracula by Bram Stoker

The aim of the Bad Bugs Book Club is to get people interested in science, specifically microbiology, by reading books (novels) in which infectious disease forms some part of the story. We also try to associate books, where possible, with some other activity or event, to widen interest, and to broaden impact.

We hope to encourage others to join, to set up their own bookclub, suggest books and accompanying activities to us, and give feedback about the books that they have read (j.verran@mmu.ac.uk).

Our bookclub comprises both microbiologists and members of the general public. We felt that this would encourage some discussion on the science – accuracy, impact etc – as well as about the book.

Dracula was written in 1897, but the year in which it was set is not mentioned. It has had enormous impact on literature and the media ever since. Currently, interest in vampires is significant, particularly across younger generations due to novels, television series and films such as the Twilight, True Blood, Vampire Diaries series, and in the recent past Buffy, Angel, the Anne Rice series (Interview with the Vampire). There is considerable discussion regarding the causes of this interest: vampire fiction seems to represent many of the fears or interests of its contemporary readership…thus Twilight and romance/celibacy; True Blood and racism; Buffy and independent women; Interview with the Vampire and, possibly homosexuality. The original novel touched upon many issues of interest to the time in which it was written.

Discussion points

1. Did you enjoy the book?
2. How did you find the presentation – the use of different types of documents to provide the narrative? Representation of accents? Difficult or easy to read?
3. Were the characters well drawn? Did you have a favourite character?
4. There are many references to modern (of that time) technology. Did you find any examples? How important was technology to the story?
5. What does Dracula look like? Does it differ from your notion of Dracula? Relationship to appearance in the current media (tv, film etc)? Differences between current and original vampires?

6. Issues of feminism were touched upon. How did you feel they were dealt with, by the author and by the characters? Can you relate the novel to the beginning of the suffragettes?
7. Some of the scenes where vampires and humans meet are very sensuously described – do you agree? How do you think the novel was received by its initial readership? How much of this ‘voluptuousness’ remains in current vampire narrative?
8. How familiar is the story to you? Did some aspects surprise you?
9. Are there any questions to be asked of transfusion scientists regarding some of the assumptions made? What are the principles of blood donation? How is ‘good blood’ differentiated? Porphyria has been used as an example of the symptoms of a vampire.
10. Microbiology. The only direct mention is that Van Helsing owes Seward his life because Seward saved him from dying of gangrene. However, what diseases are reminiscent of symptoms/conditions described? Tuberculosis? Syphilis? Rabies? And more recently, HIV?
11. What relationships can you identify between vampirism and infectious disease? Use of vampire stories to encourage discussion on disease transmission? Prevention? Treatment?
12. Have you read any other novels about vampires? How do they compare with Dracula? Do the powers of the vampires differ?

Joanna Verran

Bad Bugs Bookclub Reading Guide: The Satan Bug by Alistair MacLean

The aim of the Bad Bugs Book Club is to get people interested in science, specifically microbiology, by reading books (novels) in which infectious disease forms some part of the story. We also try to associate books, where possible, with some other activity or event, to widen interest, and to broaden impact.

We hope to encourage others to join, to set up their own bookclub, suggest books and accompanying activities to us, and give feedback about the books that they have read (j.verran@mmu.ac.uk).

Our bookclub comprises both microbiologists and members of the general public. We felt that this would encourage some discussion on the science – accuracy, impact etc – as well as about the book.

Alistair Maclean wrote twenty-nine world-wide bestsellers, and by the 1970s was recognised as one of the outstanding popular writers of the 20th century. The Satan Bug was first published in 1962, under the pseudonym Ian Stuart. It describes the theft of microbiological cultures from an English Microbiology research establishment. The cultures were developed for germ warfare, and the pursuit of the wrong-doer was carried out by the hero, Pierre Cavell.

Some points for discussion are indicated below.

1. Did you enjoy the novel? Characters?
2. Is it outdated? If you have seen the film, do you have any points to make?
3. How much science did you find in the book? Did you feel that it was well researched/described?
4. What individual identification characteristics were used in the film (eg for security)?
5. Were you convinced by the plot?
6. How accurate is the microbiology?
7. Why are 'botulinus' and polio chosen as the worst threats of the time? What do you know about these two diseases?
8. Can the book be used to consider issues of germ warfare/bioterrorism today?

9. Mordon is presumably based on Porton Down (when were people most ‘scared’ of that location/institution?). Some of the work described there encompasses a range of infections:
   ..’sterling work against anthrax, polio, Asian flu (1957) and other diseases’. Were these diseases of current concern in the 1960s?
   ...’scientists have either discovered or refined to their purest and most deadly forms the germs for causing plague, typhus, smallpox, rabbit and undulant fever in man; hog cholera, fowlpest, Newcastle disease, rinderpest, foot-and-mouth, glanders and anthrax in livestock; and blights like the Japanese beetle, European corn borer, Mediterranean fruit fly, boll weevil, citrus cancer, wheat rust and heaven knows what in plants. All very useful in either limited or all-out warfare’. What do you know about these infections?
10. What diseases have been used in ‘germ warfare’ in history – can you make a timeline?
11. Are there other thriller/murder novels that are better suited (more accurate, more up to date etc) to the topic?
12. Are there any activities related to the book which could be usefully related to current public understanding of microbiology?

Joanna Verran

Bad Bugs Bookclub Reading Guide: The Street Philosopher by Matthew Plampin

The aim of the Bad Bugs Book Club is to get people interested in science, specifically microbiology, by reading books (novels) in which infectious disease forms some part of the story. We also try to associate books, where possible, with some other activity or event, to widen interest, and to broaden impact.

We hope to encourage others to join, to set up their own bookclub, suggest books and accompanying activities to us, and give feedback about the books that they have read (j.verran@mmu.ac.uk).

Our bookclub comprises both microbiologists and members of the general public. We felt that this would encourage some discussion on the science – accuracy, impact etc – as well as about the book.

The Street Philosopher (2009) describes the experiences of three news reporters during the Crimean War, their contact with various characters there – soldiers, senior officers and others - and their subsequent activities on returning to Manchester, England. An incident in the Crimea has repercussions for them all, with various activities occurring before resolution.

Cholera was a particular problem amongst the British troops, and in general, their care was significantly neglected. Symptoms of the disease are barely described in the novel, but the war wounds are graphically described. Mary Seacole, who nursed many of the injured, plays a small part in the story, but Florence Nightingale, based in Scutari, is not mentioned at all.

Other novels also touch upon cholera to various extents. Love in the Time of Cholera (set in South America) by Gabriel Garcia Marquez (1998) again uses cholera as the backdrop to the plot, whereas in The Painted Veil (set in Asia) by W Somerset Maughan (1925), a more specific focus on the impact of cholera itself is made. Both novels were made into movies (2007 and 2006 respectively). The Rose of Sebastopol (2007) by Katharine McMahon is similar in many ways to the Street Philosopher, describing the inter-relationships of various fictional character with real events in the Crimea, but focusing more specifically on nursing and thence Florence Nightingale. Both novels describe many of the battles engaged during the war, emphasising a dreadful pointlessness. In both novels, the same settings, Balaclava in particular, are described, providing some interesting familiarity to the reader.

Cholera remains a huge global problem in times of war or disaster: the recent Haiti earthquake set the scene for a cholera outbreak, but it has been argued that the epidemic was of exogenous origin. An outbreak was anticipated after the Thailand tsunami, but it did not happen. It was not anticipated after the Japan tsunami (why not?). The disease is transmitted by the faecal-oral route, thus it is eliminated by appropriate disposal and treatment of sewage, and the provision of clean drinking water. The disease remains on the school curriculum (along with Mary Seacole and Florence Nightingale – but not the Charge of the Light Brigade). Cholera was also a significant problem in Manchester in the 19th century, slightly earlier than the Crimea period.

The main symptom of cholera is significant water loss, with death resulting from dehydration – up to 20 Litres fluid loss per day (visualise 10 two litre Coke bottles). This is caused by a toxin coded for by a virus that infects the marine bacterium *Vibrio cholerae* that causes the disease http://www.pnas.org/content/100/3/1280.full.

After the 'miasma' theory of infectious disease – where it was believed that infection was transmitted through foul-smelling air, John Snow identified contaminated water as the source of an outbreak in London in 1849. The cause of the disease was identified in 1854. The germ theory of disease was postulated in the 1870s, pure culture and Koch's postulates, which associated a given pathogen with a particular disease around 1884. So discoveries about cholera were being made almost contemporary with the Crimea (Tognitti E, 2011: The dawn of medical microbiology: germ hunters and the discovery of the cause of cholera. Journal of Medical Microbiology http://onlinelibrary.wiley.com/doi/10.1111/j.1365-2958.2005.04676.x/full

Some points for discussion:

Did you enjoy the novel?

Did you enjoy the connection between the Crimea and the Manchester Art Exhibition?

Can you find any of the Manchester streets on a map? How many of the locations are now lost? Could you follow the walk made by Cracknell and Kitson to the Trafford Arms?

Did you like the characters?

Were relationships between the characters different from those of today, or similar?

The occupations of many of the characters were fairly new – war correspondent, war artist, street philosopher. Are there parallels with news stories of today?

Did the war strategy remind you of other wars?

Did you learn anything about cholera?

Does any character in the story die of cholera?

How many times is cholera mentioned?

What is the impact of cholera on the plot?

Cholera was rampant during the Crimea. Did it kill more people that the battles?

Is there any evidence in the novel that the cause of cholera was understood?

What diseases are associated with war, past and present?

Has war had any positive impact on infectious disease prevention and control?

Joanna Verran

Bad Bugs Bookclub Reading Guide: Star of the Sea by Joseph O'Connor

The aim of the Bad Bugs Book Club is to get people interested in science, specifically microbiology, by reading books (novels) in which infectious disease forms some part of the story. We also try to associate books, where possible, with some other activity or event, to widen interest, and to broaden impact.

We hope to encourage others to join, to set up their own bookclub, suggest books and accompanying activities to us, and give feedback about the books that they have read (j.verran@mmu.ac.uk).

Our bookclub comprises both microbiologists and members of the general public. We felt that this would encourage some discussion on the science – accuracy, impact etc – as well as about the book.

In Star of the Sea, the Irish potato famine is told through stories of various characters crossing the Atlantic as part of the mass emigration of the time (1847). Relationships between the characters are revealed gradually as the narrative shifts between present and past. Contemporary illustrations and notes are included in the novel, providing additional information on the prejudices and suffering prevalent at the time.

Some points for discussion:

1. Did you enjoy the novel?

Were you aware of this part of history? How many significant aspects of history are encompassed?

Did you like the way the book was written?

2. Did you like the characters?

Did any of the characters alter during the course of the novel? If yes, is there any reason for this?

3. Over one million Irish died during the Famine, and a further million emigrated. How did you feel about the portrayal of Ireland, its culture and the prejudices prevalent at the time? Has this history impacted on the current relationship between Ireland and 'the British'? What was the impact of Irish immigration on the United States?

4. Potato blight was the underlying disease that triggered the whole story, yet was mentioned little. What information did you find in the novel? Did you find any other information? What is it caused by? Is it still a problem? What is famine fever?

5. How are crop diseases controlled nowadays? Do you know of any other examples where plants, trees etc have been introduced into different countries with significant consequences?

6. The germ theory of disease transmission was yet to be defined. Can you find information on early research into the causes of plant diseases, and mycology?

7. How many different diseases were mentioned in the novel? Which did you find most interesting? How are they transmitted? Are they still a problem?

8. How much evidence is there that the transmission, control or prevention of disease was understood at the time?

Joanna Verran

Bad Bugs Bookclub Reading Guide: Toxin by Robin Cook

The aim of the Bad Bugs Book Club is to get people interested in science, specifically microbiology, by reading books (novels) in which infectious disease forms some part of the story. We also try to associate books, where possible, with some other activity or event, to widen interest, and to broaden impact.

We hope to encourage others to join, to set up their own bookclub, suggest books and accompanying activities to us, and give feedback about the books that they have read (j.verran@mmu.ac.uk).

Our bookclub comprises both microbiologists and members of the general public. We felt that this would encourage some discussion on the science – accuracy, impact etc – as well as about the book.

Published in 1998, 'Toxin' tells a story of negligence in the beef industry resulting in the death from 0157 of a cardiac surgeon's daughter, and his pursuit of the wrongdoers.

When the book was written, *Escherichia coli* O157 was an emerging pathogen, and the story highlighted perceived horrors of the transmission of the infection, and of its symptoms. The author specialises in medical dramas – other publications include 'Coma' and 'Contagion'. The book changes from narrative of the pathogenesis of *E.coli* O157 infection to a thriller with hired killers, via some graphic descriptions of slaughterhouses and lots of public information and political issues regarding the need for the interests of meat industry and public health to be independent of one another.

The subject of the book is inevitably dated; it was written when *E.coli* O157 was an emerging pathogen, and the infection, including haemolytic uraemic syndrome (HUS) was relatively new. The novel provides plenty of data concerning incidence of infections at that time. Reading this book could stimulate discussion on the food processing industry, food-borne illness, emerging diseases, the representation of infectious disease in the media, and there is plenty of opportunity for exploration of the importance of *E.coli* (in a scientifically interested group).

Questions:

Did you like the book?

Did you enjoy the development of the plot?

How did you feel about the slaughterhouse descriptions? Do you think they were/are representative?

How interesting is this to a non-microbiology readership? Is there too much scientific jargon, or is it useful science?

Did you like the characters?

Many political issues were touched upon. Do you think the issues addressed were well explained?

Were the examples given of O157 outbreaks real? What other outbreaks are you aware of? Are there other types of *E.coli* that cause foodborne illness?

How did O157 get into that particular burger?

What other foodborne infections are mentioned in the novel?

Can the novel be used in microbiology education/science literacy?

Did the novel flag a concern of that time? Were there consequences to the publication of the novel? Is there any underlying principle that can be taken from the story that is not dated?

Extension questions focusing on the microbiology could include:

E.coli O157 has been designated an ‘emerging disease’. What other diseases could also be called ‘emerging’ (new since the 1970s)

How realistic do you think the journey of the infecting *E.coli* O157 was?

What are the symptoms of *E.coli* O157 infection in cattle?

What are the symptoms of *E.coli* O157 infection in humans?

How realistic were the symptoms described in the child?

What is the current treatment for *E.coli* O157 infection?

How good is the microbiology?

How dated is the story?

Joanna Verran

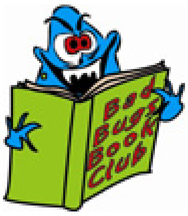

Bad Bugs Bookclub Reading guide: The Immortal Life of Henrietta Lacks by Rebecca Skloot.

The aim of the Bad Bugs Book Club is to get people interested in science, specifically microbiology, by reading books (novels) in which infectious disease forms some part of the story. We also try to associate books, where possible, with some other activity or event, to widen interest, and to broaden impact.

We hope to encourage others to join, to set up their own bookclub, suggest books and accompanying activities to us, and give feedback about the books that they have read (j.verran@mmu.ac.uk).

The Immortal Life of Henrietta Lacks was published in 2010. The book tells the story of Henrietta, a 'colored' American woman. She develops a particularly aggressive form of cervical cancer, and dies in 1951. Cells taken from the tumour when she was alive become the first able to grow in the laboratory. The story of these HeLa cells is told from three different perspectives: the life of the woman from whom the cells were taken; the journey of the author as she pieces together the story of the woman and her family – who were unaware of their mother's cells replicating in every tissue culture laboratory across the globe; and the impact of HeLa cells on science, health and the scientists who worked on the cell line – many familiar tissue culture and virology pioneers.

The book has received many awards, and is recommended for several bookclubs (including the TV bookclub. One example of a reading guide that focuses on fairly detailed aspects is http://oleanpubliclibrary.wordpress.com/2010/08/20/reading-guide-for-the-immortal-life-of-henrietta-lacks/). The BBC Documentary noted in the book 'The Way of All Flesh' is now available online (eg www.topdocumentaryfilms.com).

The book provides much opportunity for exploring many different issues such as ethics, clinical trials, informed consent, the development of science, scientists,

virology, tissue culture and so on. Further research is possible, for example finding out more about the scientists, identifying studies which were not what we would now deem ‘ethical’, investigating the scientific publications describing the advances made using HeLa cells.

‘It should be recommended reading for students starting out on a biomedical career’. David Spratt, review in July 2011 Institute of Biomedical Science Journal.

Some suggested questions

1. Did you enjoy the book? Did it affect you at all?
2. There were three parallel narratives (her family, her cells and the author’s quest) – did you think this worked? Did you think that the narratives were presented differently?
3. Did you like the characters? Henrietta and her family; the author; the scientists.
4. Did you know the story?
5. Did you find the science accessible/interesting/new?
6. Any comments on the ethical dimensions? Were they excusable for the time?
7. Do you know of any other ethically controversial stories?
8. Racial discrimination was embedded in the culture of the time. How did you feel about this?
9. Do you think that the family were badly treated? If yes, in what ways, and how could they have been better treated? If no, why not?
10. Do you think the book could be used in undergraduate or postgraduate teaching? If yes, in what ways? What additional activities could be encouraged/topics explored?

Joanna Verran

Bad Bugs Bookclub Reading Guide: I am Legend by Richard Matheson

The aim of the Bad Bugs Book Club is to get people interested in science, specifically microbiology, by reading books (novels) in which infectious disease forms some part of the story. We also try to associate books, where possible, with some other activity or event, to widen interest, and to broaden impact.

We hope to encourage others to join, to set up their own bookclub, suggest books and accompanying activities to us, and give feedback about the books that they have read (j.verran@mmu.ac.uk).

Our bookclub comprises both microbiologists and members of the general public. We felt that this would encourage some discussion on the science – accuracy, impact etc – as well as about the book.

I am Legend by Robert Matheson (1945) tells of the seemingly last survivor of pandemic which conveys vampire/zombie-type symptoms. He tries to identify the cause and a cure. The meeting was supplemented by screening of the three movies based on the book (The Last Man on Earth, the Omega Man, I am Legend) across a week.

Questions for discussion

1. Did you like the book? Is it science fiction? Horror?
2. The book was written in 1954. Do you think it has dated?
3. Essentially there is only one character – what do you make of him? What does he look like? Does his personality change over the 3 years described in the book?
4. How long after the initial epidemic does the book start? Why are only some chapters dated?
5. Why is Robert Neville immune?
6. The book describes plenty of medical science – is it good science? Neville learns a lot of microbiology – is this likely?
7. The role of a 'germ' in the pandemic is apparent. The book describes a lot of microbiology.
    a. Was it clearly explained?
    b. How did the plague begin?
    c. What was the agent?
    d. Spores?
    e. Bacteriophage?

   f. Are any known diseases with any similarities in epidemiology/pathogenesis?
   g. Would serum prevent/cure?
8. How likely is a new global and lethal pandemic?
9. Neville had to explore many myths – what were they, and could he relate them to the disease epidemiology? What myths did he explore? Were the infected actually vampires?
10. There are two sorts of vampires - live and dead vampires – were the differences clear?
11. Do you think the living people were there because the bacteria mutated?
12. How did you feel about him being killed at the end? Did his victors have a 'valid' reason for killing him? What reasons were given?
13. If you watched any of the films - How is the story interpreted in the different films? How are scientists portrayed in the different films?
14. Can this novel/novel+film(s) be used in (microbiology/science) education?

Joanna Verran

Bad Bugs Bookclub Reading Guide: World War Z by Max Brooks

The aim of the Bad Bugs Book Club is to get people interested in science, specifically microbiology, by reading books (novels) in which infectious disease forms some part of the story. We also try to associate books, where possible, with some other activity or event, to widen interest, and to broaden impact.

We hope to encourage others to join, to set up their own bookclub, suggest books and accompanying activities to us, and give feedback about the books that they have read (j.verran@mmu.ac.uk).

Our bookclub comprises both microbiologists and members of the general public. We felt that this would encourage some discussion on the science – accuracy, impact etc – as well as about the book.

World War Z by Max Brooks narrates the experiences of a number of key survivors from the Zombie war that had effectively destroyed civilisation. They are all interviewed by a journalist, who is now publishing the book to reveal the human dimension to the crisis– the government who had commissioned the work was only interested in data/statistics etc. (The meeting could be accompanied by a film screening, but the film does not reflect the book well.)

Suggested questions for discussion

1. Did you enjoy the novel? Did any of the characters create an impression on you?
2. Why do you think there is so much interest in zombies currently?
3. What other books/films describe similar (end of the world) scenarios? How does this book compare with them in terms of: Science? Humanity? Politics? Current attitudes and events?
4. Do you think the story will date? When was the story set? When did the events happen? Did this have any impact on the way in which the pandemic was detected/managed?
5. How did the pandemic emerge?
6. In what ways, if any, does zombieism reflect infectious disease? What were the symptoms? How was the disease spread? Are there any existing diseases with similar epidemiologies?
7. Was defensive action taken quickly enough? What actions were taken? Were they effective? What was learnt?

8. What prevention measures were taken?
9. What control measures were taken?
10. How was the world managed after the different plans were enforced?
11. What learning about microbiology could be derived from the novel?
12. Were you convinced as to the likelihood of the events described? Explain!
13. What exercises could students be assigned to enhance learning of science/microbiology from the story?
14. How easily/well do you think the story will be translated into a movie?
15. Did any particular incidents have an impact on you?
16. Do you think something like this could happen in ‘real life’?

Joanna Verran

Bad Bugs Bookclub Reading Guide : 28 stories of AIDS in Africa by Stephanie Nolen

The aim of the Bad Bugs Book Club is to get people interested in science, specifically microbiology, by reading books (novels) in which infectious disease forms some part of the story. We also try to associate books, where possible, with some other activity or event, to widen interest, and to broaden impact.

We hope to encourage others to join, to set up their own bookclub, suggest books and accompanying activities to us, and give feedback about the books that they have read (j.verran@mmu.ac.uk).

Our bookclub comprises both microbiologists and members of the general public. We felt that this would encourage some discussion on the science – accuracy, impact etc – as well as about the book.

28 stories of AIDS in Africa by Stephanie Nolen is just that: 28 individual experiences of HIV/AIDS. The author is a journalist who narrates the stories, each of which has some unique message about the disease, whether it be related to personal aspects, such as maintenance of secrecy, an unwillingness to divorce, prejudice, denial, access to treatment, and political aspects such as civil war, capitalism, corruption, inflation, or history. There is a vague chronology, where key changes such as access to treatment is improved, treatment of pregnant women is implemented, second line drugs become available etc.

There are many questions that can be asked to stimulate discussion about the book. They can relate specifically to AIDS, or be more general in terms of accessibility and impact of the book itself.

Questions

1. Did you like the book? The style of writing (journalistic?)? Did you relate to any of the characters?
2. Did you learn anything about HIV/AIDS the virus? Does HIV cause AIDS? What about the AIDS-deniers?
3. How is AIDS transmitted? How was it primarily transmitted in the book?
4. What are the prevention/treatment strategies?
5. Did any aspect of the book affect you particularly?

    a. How much did you know about Africa? How did different countries respond to AIDS?
    b. Did the story of any particular country seem especially moving?
    c. Thinking about the number of AIDS orphans growing up, how would the countries be run in the future?
    d. The book explored how AIDS prevalence and infection was related to other phenomena – war, refugees, industry, prostitution. Did you find this interesting?
6. To me, the following issues were thought-provoking. What about you?
    a. The number of ‘first people to be open’
    b. Scandal of divorce
    c. Women thrown out of home
    d. School fees in poor countries
    e. Political/governmental/historical aspects/unfairness
    f. Wealthier more infected that the rural poor
    g. Grandparents bringing up orphans
7. Did you notice any chronology?
8. What were the key changes in Africa’s reaction to the pandemic that helped in control?
9. Have things changed recently?
10. Do you think that the AIDS pandemic in Africa has global implications?
11. What educational activities might the book stimulate? Is it relevant to the school curriculum?
12. Did the book change anything about you/make you want to do something?

Joanna Verran

Bad Bugs Bookclub Reading guide: The Island by Victoria Hislop

The aim of the Bad Bugs Book Club is to get people interested in science, specifically microbiology, by reading books (novels) in which infectious disease forms some part of the story. We also try to associate books, where possible, with some other activity or event, to widen interest, and to broaden impact.

We hope to encourage others to join, to set up their own bookclub, suggest books and accompanying activities to us, and give feedback about the books that they have read (j.verran@mmu.ac.uk).

Our bookclub comprises both microbiologists and members of the general public. We felt that this would encourage some discussion on the science – accuracy, impact etc – as well as about the book.

The Island is Victoria Hislop's first novel (2005). It describes the relationships within a Cretan family across 4 generations spanning the latter half of the 20th century. Their story is intertwined with that of Spinalonga, an island off the coast of Crete, which housed Greece's lepers until the 1950s, when successful antibiotic treatment enabled them to be cured and 'freed' from the island. Our meeting took place in a Greek restaurant, over a meze.

Some points for discussion

1. Did you like the book and the plot?
2. Did you like the characters? Did you feel that the actions of the different characters were believable? Describe examples. How did the proximity of Spinalonga affect the behaviour of the characters?
3. If lepers are 'unclean', how 'clean' were the characters without leprosy?
4. The story is uncovered due to the interest of a contemporary historian. What contribution did her story make to the overall plot? Did it link the present with the past, or was it unnecessary?
5. Did the novel convey much of the history and character of Crete?
6. Have you been to Spinalonga?
7. How much did you know about leprosy before you read the book? Did the book change any of your preconceptions about the disease? Were the symptoms of leprosy described in the novel as you would have imagined? What are the symptoms and pathogenesis of leprosy?

8. Why do you think leprosy is so well known as an infectious disease? How common was it? How common were leper colonies in Europe and developed countries in the 20th century (and now)?
9. Is leprosy still of global importance?
10. How different is the prevention of leprosy from the prevention of other infectious diseases?
11. How accurate was the science? (traditional medicine, treatment, diagnosis, clinical studies). How important do you think the impact of antibiotics was in the mid-20th century? What are our perceptions of antibiotics nowadays?

Joanna Verran

Bad Bugs Bookclub Reading Guide: Nemesis by Philip Roth

The aim of the Bad Bugs Book Club is to get people interested in science, specifically microbiology, by reading books (novels) in which infectious disease forms some part of the story. We also try to associate books, where possible, with some other activity or event, to widen interest, and to broaden impact.

We hope to encourage others to join, to set up their own bookclub, suggest books and accompanying activities to us, and give feedback about the books that they have read (j.verran@mmu.ac.uk).

Our bookclub comprises both microbiologists and members of the general public. We felt that this would encourage some discussion on the science – accuracy, impact etc – as well as about the book.

'Nemesis' (2010) describes an outbreak of polio in a Jewish area of Newark New Jersey in 1944. The story is written in 1971, narrated by a child who was affected during the outbreak. The child in turn describes the impact of the events in the life of Bucky Cantor, the school playground instructor.

1. Did you like the book?
2. What was your impression of the main character, Bucky Cantor?
3. Bucky seems unable to accept any good fortune in his life. Why do you think this is the case?
4. How did the other characters impact on the development of the story? How did their personalities contrast with or complement Bucky's?
5. Philip Roth is an award-winning American author. Is this book typical of his stories?
6. What do you know about polio? Symptoms, epidemiology/history, transmission, treatment, prevention etc.
7. What ideas did the characters in the novel have about the transmission of polio? How was this fear of the invisible unknown interpreted with regards to transmission routes? What other battles (of any kind) were being fought at the time?
8. What is the current global situation regarding polio control?
9. Polio is successfully prevented by a vaccine. Is polio one of the best examples of successful vaccine development? What other diseases are prevented/eradicated by vaccines?

10. What different types of vaccines are available? Which type(s) of vaccine is used against polio?
11. What diseases have you been immunised against?
12. What do you know about post-polio syndrome?
13. Do you think you could trace the epidemiology of this particular outbreak as it affected Bucky? Do you think he was the Typhoid Mary? (who was Typhoid Mary?) Do you think it matters whether or not he was the ‘cause’?

Joanna Verran

This book was particularly valuable for the bookclub, and was used in several meetings for different audiences. Questions arising from subsequent meetings:

What/who is the Nemesis of the title?

Why are the outbreaks in the summer?

How much was known about polio at the time the book was set (1944, 1971) and written (2010)? (It is certainly mentioned in the book that it was caused by a virus)

Can you use this book to learn about polio? What else can you learn from the book?

Can you see any parallels with the recent coronavirus pandemic?

Bad Bugs Bookclub Reading Guide: The Painted Veil by W Somerset Maugham

The aim of the Bad Bugs Book Club is to get people interested in science, specifically microbiology, by reading books (novels) in which infectious disease forms some part of the story. We also try to associate books, where possible, with some other activity or event, to widen interest, and to broaden impact.

We hope to encourage others to join, to set up their own bookclub, suggest books and accompanying activities to us, and give feedback about the books that they have read (j.verran@mmu.ac.uk).

Our bookclub comprises both microbiologists and members of the general public. We felt that this would encourage some discussion on the science – accuracy, impact etc – as well as about the book.

The book (1925) is set in China/Hong Kong during the times of British colonialism. Walter Vane is the bacteriologist ('no great shakes', 'no great fry') investigating cholera. His wife is an attractive but superficial woman: she has an affair with the Assistant Colonial Secretary and Walter subsequently forces her to travel with him to a village in China where cholera is raging. He dies of cholera, she ultimately thrives, relishing the activity, space, beauty and reality (comparing previous experience of a 'duck-pond' to that of 'the sea') – having opportunity to work, reflect etc – and returns (pregnant, unknown father) to London for the Bahamas with her father.

There is not a lot of information about cholera in the book, so it might be useful to watch a movie alongside reading the book. (There has been more than one movie – the most recent in 2006 (2 hours).

1. What did you think of the book/film? How do they differ? Did you have a preference?
2. Why do you think there are differences in the plot between the book and the film?
3. When do you think the book was set? What attitudes are evident? What was happening in the world/China at the time?
4. Did you like the characters? Were they well drawn? Somerset Maugham is well-known for his portrayal of women – do you agree?
5. There is much thought and philosophising by Kitty, who narrates the story. Did you find this interesting? Was this thoughfulness also captured in the movie?
6. Why did Walter love Kitty? What did he look like (book/film)? How did he and Kitty's feelings for one another alter (book/film)?

7. How much cholera information is in the book? Film? What is the impact of cholera on the story?
8. What was known about cholera at the time? How much information could be found about cholera that was relevant then and now? How was/it prevented/treated?

Joanna Verran

Bad Bugs Bookclub Reading Guide: Intuition by Allegra Goodman

The aim of the Bad Bugs Book Club is to get people interested in science, specifically microbiology, by reading books (novels) in which infectious disease forms some part of the story. We also try to associate books, where possible, with some other activity or event, to widen interest, and to broaden impact.

We hope to encourage others to join, to set up their own bookclub, suggest books and accompanying activities to us, and give feedback about the books that they have read (j.verran@mmu.ac.uk).

Intuition (2006) describes the activity of a research laboratory searching for a cure for cancer, and is set in 1987. A strain of respiratory syncytial virus shows promise in animal studies and the novel describes how some of the research leaders and team subsequently promote their discovery, and others question the findings.

Questions

The questions that can be asked related to this novel can address laboratory research in some detail. The conduct of laboratory research and researchers, and the research laboratory environment can be explored. Some of the questions listed below are rather specific, but others are more general. A book group that comprised research scientists/students and the general public would be especially valuable for this novel.

1. Did you enjoy the book?
2. There are several different characters who play key roles in the development of the story. Did you like the characters? Did you feel that they were honest/likely to behave as they did? Did you have a favourite character? Why?

3. If you are not familiar with life in research, were any aspects of the story surprising to you? If you are familiar with working in a research laboratory, do you think that the roles and responsibilities were portrayed accurately?
4. What does the book say to you about research culture and scientific integrity? Who owns research? The grant awarder, awardee and their research staff? The public?
5. Where does funding for research come from? At what point do scientists disclose their findings – and to whom (peers? Funders? Public? Press releases?)
6. Where does research happen? Are there significant external forces affecting decisions made by researchers, or institutions in which research is taking place?
7. How were women, and women scientists portrayed? How is teaching portrayed?
8. Was there sufficient science content with regard to the research projects ongoing? Did you perceive any ethical issues?
9. Was the animal research necessary? How many mice/animal studies are necessary for publication? Does the breed of mice that is described actually exist?
10. What were the limitations of Cliff's results? Was the experimental design acceptable? Data recording? Reproducibility of results? What are the requirements for publication in a peer reviewed journal?
11. What were the findings of the research that caused the excitement? What is respiratory syncytial virus? Can viruses be used to cure or treat cancers? Do any viruses cause cancer?
12. What happened to the characters in the end of the novel? Was the fate of each appropriate?

13. Do you think this book has value as a focus for discussion considering scientific integrity, culture, research etc? How would it be viewed by the general public/student/researchers?

Joanna Verran

Bad Bugs Bookclub Reading Guide: Arrowsmith by Sinclair Lewis

The aim of the Bad Bugs Book Club is to get people interested in science, specifically microbiology, by reading books (novels) in which infectious disease forms some part of the story. We also try to associate books, where possible, with some other activity or event, to widen interest, and to broaden impact.

We hope to encourage others to join, to set up their own bookclub, suggest books and accompanying activities to us, and give feedback about the books that they have read (j.verran@mmu.ac.uk).

Our bookclub comprises both microbiologists and members of the general public. We felt that this would encourage some discussion on the science – accuracy, impact etc – as well as about the book.

Arrowsmith (1924) describes the life of Martin Arrowsmith, a doctor, whose passion is research but whose career leads him along different routes, where he encounters a range of individuals, professions and attitudes. The development of controlled research experimentation is relatively new in 1924 – it is only 50 years or so since the pioneer microbiologists including Pasteur and Koch made their discoveries – and aspects of clinical research design, ethics, commercialism, publication are touched upon.

Questions

1. Did you enjoy the novel? Writing style? Story?
2. Did you like the characters? Lewis is known for his portrayal of women – did you feel that Arrowsmith's two wives, and other women in the novel were described well – did you like them? Were they strong women?
3. Gottlieb is Arrowsmith's hero. Was Gottlieb's character modelled on anyone? Was Gottlieb well equipped for a career in research? Management? Commerce/business? What characteristics did Arrowsmith's fellow students display in their subsequent career choices?
4. Arrowsmith was faced with many choices throughout the novel. Do you think he made correct choices – what do you think of his personality? Did he make an heroic journey?

5. Is the pursuit of scientific research accurately described? Are issues of commercialisation of research, and funding of relevance today? What about the celebrity scientist? The publication of research – open access to findings, and the publication of negative results?
6. How relevant is the phage discovery to today's science? Phage therapy was somewhat abandoned when antibiotics were discovered, but there is a relative resurgence now – why might this be? How accurate was the phage-related science? How successful was phage in the treatment of the cholera outbreak?
7. How was research in virology progressing at the time the book was written?
8. Was the laboratory practice of microbiology portrayed well? Would you have been comfortable in the anthrax class?
9. During Arrowsmith's work in Public Health, many different diseases were encountered. What were they, and were the measures taken appropriate?
10. What do you know about Lewis' collaborator, Paul de Kruif?
11. The book was written between the wars. Is there any evidence of this in some of the personalities/prejudices/activities.

Joanna Verran

Bad Bugs Bookclub Reading Guide: The Air we Breathe by Andrea Barrett

The aim of the Bad Bugs Book Club is to get people interested in science, specifically microbiology, by reading books (novels) in which infectious disease forms some part of the story. We also try to associate books, where possible, with some other activity or event, to widen interest, and to broaden impact.

We hope to encourage others to join, to set up their own bookclub, suggest books and accompanying activities to us, and give feedback about the books that they have read (j.verran@mmu.ac.uk).

Our bookclub comprises both microbiologists and members of the general public. We felt that this would encourage some discussion on the science – accuracy, impact etc – as well as about the book.

The Air We Breathe by Andrea Barrett describes the activities of a small community in a tuberculosis sanatorium, their social interactions and treatments.

1. Did you enjoy the book? Did you like the characters? There were many different characters in the novel – were you able to follow the business of them all?
2. Who is writing the book, and why? What type of people were in the sanatorium, and why?
3. What are the symptoms of TB?

4. What is the treatment for TB that is described in the novel? Is there historical or epidemiological support for the treatment? Were the rules sensible or excessive?
5. Did the discussion group have any impact on health? Did you enjoy the different stories?
6. Aspects of chemistry are included throughout the novel – how many different industrial/applied or 'pure' processes are described? X-rays/Roentgen rays receive a significant amount of attention. They were used to monitor the progress of the patients. What impact might the X-rays have on the health of the population?
7. Why do you think Mendeleev's work is of importance in the story?
8. Did you enjoy the investigative science (eg Eudora and her X-ray work)?
9. There were many parallel situations described in the novel – which can you identify?
10. Why is there a family tree at the end of the book? Did you find it useful?
11. Thomas Mann's Magic Mountain is also about TB. Have you read it? Would it be useful for the Bad Bugs Boookclub? Do you see any comparisons/differences between the novels?
12. There has been significant historical impact of TB, with many famous victims (do you know any?). Why was it called the White plague?
13. What is the global impact of TB today? How is it treated?

Joanna Verran

Bad Bugs Bookclub Reading Guide: The Ghost Map by Steven Johnson

The aim of the Bad Bugs Book Club is to get people interested in science, specifically microbiology, by reading books (novels) in which infectious disease forms some part of the story. We also try to associate books, where possible, with some other activity or event, to widen interest, and to broaden impact.

We hope to encourage others to join, to set up their own bookclub, suggest books and accompanying activities to us, and give feedback about the books that they have read (j.verran@mmu.ac.uk).

The Ghost Map (2006) describes the work of John Snow and the less well-known Henry Whitehead in their attempts to identify the source of a cholera outbreak in London in 1854. The Bookclub meeting was held during National Science and Engineering Week, close to World Water Day. The book is not a novel, but a narrative of the historical information available at the time: the author provides additional and current perspectives on the importance of mapping, and city living.

1. The book is not a novel, although it does tell a story. Did you think the approach was successful/effective. Did you like the book?
2. The book only used documented fact in its narrative. Were any personality traits of the various characters conveyed?
3. Do you think that any of the issues encountered at the time are still relevant today?
4. Did you like the contemporary nuances brought into the narrative – city life, mapping, apocalypse (nuclear), H5N1 etc etc.
5. Was the microbiology well explained?
6. At the time of the outbreak, how significant was cholera to the population of Britain – at home and abroad?
7. Would you have believed the miasma theory?

8. What large scale engineering works were taking place in London at the time of the book?
9. How important was Snow and Whitehead’s work in terms of epidemiology and public health control.
10. Cholera causes pandemics (world-wide outbreaks). What other diseases are associated with pandemics?
11. How are the microbiological causes of outbreaks ‘typed’?
12. How important in world health is cholera today? Is it still a focus for microbiology research? What recent outbreaks have taken place? How is cholera treated/prevented? Is there a vaccine?

Joanna Verran

A Wind in the Door by Madeleine L'Engle: Reading guide

The aim of the Bad Bugs Book Club is to get people interested in science, specifically microbiology, by reading books (novels) in which infectious disease forms some part of the story. We also try to associate books, where possible, with some other activity or event, to widen interest, and to broaden impact.

We hope to encourage others to join, to set up their own bookclub, suggest books and accompanying activities to us, and give feedback about the books that they have read (j.verran@mmu.ac.uk).

Our bookclub comprises both microbiologists and members of the general public. We felt that this would encourage some discussion on the science – accuracy, impact etc – as well as about the book.

A Wind in the Door (1973) is the second in a series by Madeleine L'Engle. The book is classified as 'young adult science fantasy'. It focuses on aspects of the cell – the mitochondrion in particular.

A child is ill with a mitochondrial disease. His mother is trying to find a cure (she has an electron microscope in the house); his father is tackling galactic issues. His sister provides the focus for the story and its fantastic progression involving travel into the cell, fighting evil, working together to triumph over all.

Questions

This book can be discussed without reference to underpinning science – there are many religious and scientific aspects to consider. The questions below combine scientific and general questions.

1. Did you like the book? There was a lot of new language being introduced throughout – what is your view on this?
2. Did you like the characters? Did you have any favourite characters?
3. Did you think there was a lot of science in the book, or not? If yes, what did you think were the key concepts?
4. What are mitochondria? What are farandolae?
5. What is mitochondrial disease?
6. Is there any microbiology in the book?
7. Have you come across the work of Lynn Margulis? How does this relate to the story?
8. Does chaos theory or fractal science have a role in the novel?
9. There are many occasions in the book where relationships, love, connectivity are essential. What impression did they make on you? Do they connect well with some of the more scientific aspects?
10. Would you read another book in the series?

Joanna Verran

Bad Bugs Bookclub Reading guide Fever by Mary Beth Keane

The aim of the Bad Bugs Book Club is to get people interested in science, specifically microbiology, by reading books (novels) in which infectious disease forms some part of the story. We also try to associate books, where possible, with some other activity or event, to widen interest, and to broaden impact.

We hope to encourage others to join, to set up their own bookclub, suggest books and accompanying activities to us, and give feedback about the books that they have read (j.verran@mmu.ac.uk).

Fever (2013) is a fictional narrative based on the life of ‘Typhoid Mary’, an Irish cook in New York who caused several typhoid outbreaks in the city during the early twentieth century, because she was a typhoid carrier. The story follows many factual lines, but also introduces new characters, as well as some interesting narrative about living conditions, class systems and other prevalent prejudices.

Mary is ‘pursued’ by a doctor/epidemiologist, who has identified her as the cause of the outbreaks in the homes where she was employed. Mary refused treatment offered her (surgery), and was then effectively imprisoned on North Brother Island for many years. She was released because other carriers and outbreaks were identified, but she disregarded her conditions of release (no catering for others), and was returned to the island in after causing another outbreak. She died on the island.

1. Did you enjoy the book?

2. Mary’s personality is often noted (eg Wikipedia!). Do you think the novel gave appropriate attention to her personality? Did you like her? Should she have behaved differently?

3. Why was ‘the hat’ important to the story?

4. Alfred was a fictitious character. How did his experiences contribute to the development of the story?

5. Were you familiar with the story of Typhoid Mary? If yes, are there any differences in the outbreaks in the novel and in factual literature? If no, would you like to know more?

6. Living conditions and lifestyles of the time were key to the story. Did you think that the differences in class etc were well described? What attributes did Mary have that may have contributed to the way she was treated? What has happened to North Brother Island?

7. In the time that the story was set, what was the prevalent understanding of the transmission of infectious disease?

8. How is typhoid transmitted? How do you think Mary transmitted the typhoid bacteria to her 'victims'? Was she a bad cook? Did you notice points in the story when disease transmission may have occurred?

9. Do you think Mary was treated fairly? How were other typhoid carriers treated later in the novel? Are there more recent diseases where infected individuals were not treated very sympathetically?

10. What role did science and scientists play in Mary's story? Do you think they could have acted differently?

11. If this book were made into a film, who would play the parts of Mary, Soper, Alfred and Josephine Baker?

12. What do you know about typhoid? Why is it not present in this country today? Is it present anywhere else in the world? What is the current epidemiology of typhoid? How do you avoid contracting the disease in countries where it is endemic?

13. *Salmonella typhi* is the bacterium responsible for typhoid. What else do you know about the genus Salmonella?

14. As a genus, *Salmonella* is extremely well-researched (as is *Escherichia coli* – how do these bacteria compare?). What current research is taking place on this microorganism?

Joanna Verran

Bad Bugs Bookclub Reading Guide: Microbe Hunters by Paul de Kruif

The aim of the Bad Bugs Book Club is to get people interested in science, specifically microbiology, by reading books (novels) in which infectious disease forms some part of the story. We also try to associate books, where possible, with some other activity or event, to widen interest, and to broaden impact.

We hope to encourage others to join, to set up their own bookclub, suggest books and accompanying activities to us, and give feedback about the books that they have read (j.verran@mmu.ac.uk)

Our bookclub comprises both microbiologists and members of the general public. We felt that this would encourage some discussion on the science – accuracy, impact etc – as well as about the book.

Microbe Hunters by Paul de Kruif was published in 1926. De Kruif was a microbiologist. He had acted as medical consultant to Sinclair Lewis for his award winning novel Arrowsmith (also read by the Bookclub), and received 25% of the profits. Microbe Hunters is the best known of his books. It describes, through a series of short stories, the discoveries of the pioneer microbiologists from van Leeuwenhoek in the 17th century to Ehrlich's 1910 discovery of Salvarsan, the first targeted antimicrobial treatment. The book has often been credited as having a significant, inspirational impact on many scientists' choice of career.

Discussion points

This book is different from many others which the bookclub has read. The factual but informal short story format would allow easy translation to use in the undergraduate (and postgraduate curriculum). Most of the content is science-facing, so many of the discussion points can involve microbiology to different levels of detail, thus most of the questions below can be explored by students as well as the general readership. Many of the issues are of relevance today. Several of these scientists were the subject of movie 'biopics' in the 1930s. These films can be viewed alongside the book discussion.

1. Did you like the book? What about the writing style and the personalisation of the characters? Were there any particular aspects of the book that impressed you?

2. Did you have a favourite chapter/scientist/discovery/scientist? If yes, who and why? For example:

    a. Louis Pasteur. What do you think of him and his story? What other pioneer microbiologists did he work with? What were his key discoveries? How many different diseases were mentioned?

    b. Robert Koch. What do you think of him and his story? What other pioneer microbiologists did he work with? What were his key discoveries?

    c. Ehrlich. What do you think of him and his story? What other pioneer microbiologists did he work with? What were his key discoveries? What has happened in the intervening 100 years in terms of magic bullets?

3. Did your reading of the book change your ideas on the nature of science and scientists? Were there any particular attributes not necessarily associated with scientists that were key to the discoveries? Why is the stereotypical scientist dull, 'crusty' and male when the characters in the book are so vibrant (and male)? Is this changing? If so, why?

4. Do you think the book still has value despite being written almost 90 years ago? What issues face today's pioneer scientists that are different from the 'microbe hunters'?

5. Can you see any parallels in today's scientists tackling emerging disease? What diseases have emerged in recent decades (traditionally since the 1970s).

6. What are the biggest problems facing us today in terms of infections and infectious diseases?

7. Looking at the discoveries of Ehrlich, how often are/were dyes used in microbiology, and in what context? What about the 'side chain theory'?

8. If another volume of Microbe Hunters were to be written today, who would you want information about? Any women?!

Joanna Verran

Bad Bugs Bookclub Reading Guide: The Last Town on Earth by Thomas Mullen

The aim of the Bad Bugs Book Club is to get people interested in science, specifically microbiology, by reading books (novels) in which infectious disease forms some part of the story. We also try to associate books, where possible, with some other activity or event, to widen interest, and to broaden impact.

We hope to encourage others to join, to set up their own bookclub, suggest books and accompanying activities to us, and give feedback about the books that they have read (j.verran@mmu.ac.uk).

Our bookclub comprises both microbiologists and members of the general public. We felt that this would encourage some discussion on the science – accuracy, impact etc – as well as about the book.

The novel describes how one fictitious remote sawmill town in NorthWest America quarantined itself to prevent the 1918 influenza pandemic from spreading amongst its citizens – and the consequences of that decision. The novel also addresses many cultural and social aspects of the time, particularly America entering the war, and workers' rights. Across the world, influenza killed more people during the war than were killed by the war itself.

Questions

1. This was the Bookclub's first novel about influenza. Are you aware of any other novels about influenza? If there are not many, why do you think this is the case? (NB: There were many books published from 2018 – why?)
2. The author notes that he did not know anything about the 1918 pandemic. Did you? Is this typical – of the US, or the UK?
3. Did you enjoy the novel? Any particular aspects?
4. Did you feel that the motivation for the shooting of the first man, and the murder of the second man were well explained? Were the characters of Philip and Graham convincing to you? Did you particularly like/dislike any part of the novel?
5. Have you ever had the flu? How did you know that you had the flu? What were the symptoms? Have you ever been vaccinated against influenza? Why? When?
6. The World Health Organisation has an Influenza Surveillance Centre. Cases are also monitored in individual countries. How easy do you think it is to

collect data about cases of influenza during a pandemic, epidemic, or just during a ‘normal’ winter? What difficulties might arise?

7. This pandemic was particularly virulent. How do the symptoms vary from what we typically expect of influenza? Are there any other diseases with symptoms similar to those described here? Or other diseases with symptoms similar to ‘normal’ flu?
8. How did the characters in the novel think the disease spread? How did they prevent spread? Was there any treatment? Do you think the masks helped? How were doctors trained at that time? What was known about the transmission of disease?
9. The main strategy of the town was isolation and separation from an enemy – the disease. Are you aware of any other instances or examples of ‘reverse quarantine’? What about true quarantine - any examples?
10. Influenza recurs – what do you know about vaccination, emergence of new ‘pandemics’ (the recent H1N1) and how did it differ from the 1914 virus? And in what ways was it the same.
11. What are current concerns about influenza? What do you know about bird flu? Hong Kong flu? Swine flu?
12. Do you think that this novel would be useful for student learning? The general public?

Joannna Verran

Bad Bugs Bookclub Reading Guide: Petroplague by Amy Rogers

The aim of the Bad Bugs Book Club is to get people interested in science, specifically microbiology, by reading books (novels) in which infectious disease forms some part of the story. We also try to associate books, where possible, with some other activity or event, to widen interest, and to broaden impact.

We hope to encourage others to join, to set up their own bookclub, suggest books and accompanying activities to us, and give feedback about the books that they have read (j.verran@mmu.ac.uk).

Our bookclub comprises both microbiologists and members of the general public. We felt that this would encourage some discussion on the science – accuracy, impact etc – as well as about the book.

Petroplague by Amy Rogers (2011) is a thriller describing the escape, in Los Angeles, of genetically engineered bacteria which are able to break down gasoline. The consequences of this rapidly spreading infection to our oil-dependent culture are explored as scientists attempt to correct the situation.

The author is clearly very keen on science literacy and science education, and relevant notes are provided at the end of some versions of the book. There is also a website for science educators working with the novel (http://sciencethrillers.com; www.amyrogers.com). Where do you see value for the book in this context?

Discussion points

1. Did you enjoy the book? Did you like the characters? Were there any parts of the plot that you particularly liked/disliked? Were any sections unnecessary? How realistic was the plot for you?

2. The story was very fast-moving, with cinematic scale imagery. Would the novel make a good movie? Graphic novel?

3. Christina often explains the microbiology/chemistry behind her work to her River and Micky. Did you find these explanations clear/interesting/valuable to

the plot? What about the descriptions of the equipment and techniques which she uses?

4. There are several relatively technical aspects to the story which could be explored with students, and with the 'general public', for example with regards to how important they are in current research:

    a. There are 2 engineered bacteria – what are they? Do you think that the 'field-based' experiment was well planned? What went wrong?
    b. What is synthetic biology? How current is research in this area?
    c. What do you know about biofuels? There is considerable interest in biofuels amongst the research community, and in industry. Why do you think this is?
    d. What are the 2 proposed cures for the petroplague? How likely do you think they would be to succeed?
    e. What do you know about phage therapy?
    f. The petroplague spread very rapidly. Fortunately, this was also the case with the treatment. Was it all too fast?
    g. In some versions, the author notes at the end of the novel where some aspects of the story are unlikely/impossible. Do you think this information is useful/necessary? Were there any parts of the story you were uncomfortable with?

5. The plot is focused on science research and scientists, and the problems associated with an escaped genetically engineered bacterium. Do you think that there are appropriate controls for this type of work in real life?

6. Christina is studying for a PhD. Does the nature of postgraduate/University research come across clearly?

7. There is significant interest from industry in Christina's research – some of which is very negative. Do you think that commercial sponsorship of University research is appropriate/essential/important?

8. How does the novel present the 'ecoterrorists'? Collapsionarians? What is your view on such activism?

Joanna Verran

Bad Bugs Bookclub Meeting Report: Code Orange by Caroline B Cooney

The aim of the Bad Bugs Book Club is to get people interested in science, specifically microbiology, by reading books (novels) in which infectious disease forms some part of the story. We also try to associate books, where possible, with some other activity or event, to widen interest, and to broaden impact.

We hope to encourage others to join, to set up their own bookclub, suggest books and accompanying activities to us, and give feedback about the books that they have read (j.verran@mmu.ac.uk).

Our bookclub comprises both microbiologists and members of the general public. We felt that this would encourage some discussion on the science – accuracy, impact etc – as well as about the book.

Code Orange (2005) by Caroline B Cooney is a 'teen thriller'. It is set in a New York college: students (aged around 16) have each been assigned a different disease/pathogen to write a report on. Mitty Blake is allocated smallpox. The story describes his research, his exposure to material that may be contaminated with the virus, and his subsequent activities and experiences during the long incubation period of the disease.

Questions:

1. Who is the intended audience for this novel (age group)? If you were not the intended audience, did you enjoy reading the story? Did you think the characters were well drawn?

2. The format gives an opportunity for sections of extremely factual aspects of medical microbiology. How does this complement the fictional elements of the story? Does it fit comfortably with the style?

3. Did you learn much about smallpox? What strategy was used to eradicate the disease? Could/should global eradication of smallpox be used as a motivator for vaccination strategies for other diseases?

4. Smallpox has often been noted to be a 'good' candidate for bioterrorism. Why is this? Give specific examples of bioterrorism where infectious material has intentionally been used to disseminate infection.

5. Do you agree or disagree that the remaining stocks of smallpox virus should be destroyed?

6. Some versions of the novel provide bookclub questions which clearly indicate an educational (not necessarily microbiological) angle as well as the intended audience. How do the internet, terrorism and bioterrorism relate to the development of the storyline? How might this discussion impact on the intended audience.

7. The story is overtly educational, with students (16+) being the main characters. The storyline follows Mitty and his friends as they learn more about the diseases they have been assigned. This type of assignment is very common for undergraduate students – do you think that the book would be a useful adjunct in an undergraduate situation? Or any other educational environment?

Joanna Verran

Bad Bugs Bookclub Reading Guide: The First Eagle by Tony Hillerman

The aim of the Bad Bugs Book Club is to get people interested in science, specifically microbiology, by reading books (novels) in which infectious disease forms some part of the story. We also try to associate books, where possible, with some other activity or event, to widen interest, and to broaden impact.

We hope to encourage others to join, to set up their own bookclub, suggest books and accompanying activities to us, and give feedback about the books that they have read (j.verran@mmu.ac.uk).

Our bookclub comprises both microbiologists and members of the general public. We felt that this would encourage some discussion on the science – accuracy, impact etc – as well as about the book.

The First Eagle (1998) is one of a series of detective novels involving the Navajo tribal police. The novels encompass aspects of Native American life in the American West and reservations, and include interesting differentiation of the tribes who live in the area. The First Eagle describes the hunt for a missing person and a murderer. The missing person was working on *Yersinia pestis*, plague, which often emerges sporadically from an animal reservoir.

Questions

1. Did you like the novel? Would you read more of the series? Were the characters well drawn (‘the characters are imperfect’ is a cover quote)? Is the series well respected in the States?

2. Did you find the references to Native American life/life in this part of America and its geography and geology interesting?

3. Were you aware that bubonic/pneumonic plagues remain enzootic in parts of the world? Which parts?

4. What is an outbreak? Why was this particular outbreak of interest?

5. ’ If the viruses don’t get us the bacteria will’ – why is Woody, the scientist, a well-respected researcher (who has published in Science) saying this?

6. What is interleukin 6? What antibiotics are usually used to treat plague? Why is emerging resistance a problem?

7. What was Woody investigating?

8. What was Catherine Pollard doing?

9. What was the link between Catherine and Woody? What was the motive for the murder?

10. The focus of the story is on the murder/disappearance, although it centres around the people working on the plague. Why is the novel called The First Eagle? Were the forensics interesting?

11. There are numerous ‘hard science ‘ paragraphs. Did they contribute to the story for you?

12. Would this book be useful for school, college or University education?

Joanna Verran

Bad Bugs Bookclub Reading Guide: Oryx and Crake by Margaret Atwood

The aim of the Bad Bugs Book Club is to get people interested in science, specifically microbiology, by reading books (novels) in which infectious disease forms some part of the story. We also try to associate books, where possible, with some other activity or event, to widen interest, and to broaden impact.

We hope to encourage others to join, to set up their own bookclub, suggest books and accompanying activities to us, and give feedback about the books that they have read (j.verran@mmu.ac.uk).

Our bookclub comprises both microbiologists and members of the general public. We felt that this would encourage some discussion on the science – accuracy, impact etc – as well as about the book.

Oryx and Crake by Margaret Atwood (2003) is the first in a trilogy describing a future world which has, essentially, been destroyed by science. The lead character, once called Jimmy and now called Snowman, is a lone survivor who does not seem to have been manipulated in any way by the interventions that were routinely and readily available. He is looking after 'the children of Crake', who have been engineered in different ways.

1. What are your reactions to the book?

2. Jimmy is able to look back in time and reflect on people and events that are familiar to us. How far in the future do you think these events are happening? How old is Jimmy?

3. Although in the time in which the book is set is apocalyptically awful, the past narrative describes significant differences and ways of life for Jimmy and Crake as they grew up. What sort of world did they live in?

4. What sort of scientific interventions were commonplace?

5. What went wrong?

6. Despite the enormously destructive events, there seems to be at the core, a love triangle. To what extend do these human interactions affect the future of the planet? What was Oryx’s role in the story? Did you like the characters?

7. What are your reactions to the children of Crake?

8. Did you identify any religious overtones?

9. Crake was the (Gothic) ‘lone scientist’. What drove him? How does he compare to Frankenstein?

10. What happened in the end? Would you read the next two books in the series?

11. Would this book be of value in an undergraduate (or postgraduate) class discussion?

12. How important was microbiology to the story?

Joanna Verran

Bad Bugs Bookclub Reading Guide: Annihilation by Jeff Vandermeer

The aim of the Bad Bugs Book Club is to get people interested in science, specifically microbiology, by reading books (novels) in which infectious disease forms some part of the story. We also try to associate books, where possible, with some other activity or event, to widen interest, and to broaden impact.

We hope to encourage others to join, to set up their own bookclub, suggest books and accompanying activities to us, and give feedback about the books that they have read (j.verran@mmu.ac.uk).

Our bookclub comprises both microbiologists and members of the general public. We felt that this would encourage some discussion on the science – accuracy, impact etc – as well as about the book.

Annihilation is the first of the Southern Reach trilogy – all published in 2014. The author is better known for his 'steampunk' work – he is also interested in aspects of biology. Annihilation is the narrative of the twelfth expedition into Area X, given by the biologist member of the team. Area X is an uninhabited area of America, which is going 'back to nature'. An event thirty years ago was described, in a low-key manner in the media, as 'a localised environmental catastrophe stemming from experimental military research'. Local inhabitants had disappeared, and expeditions were sent by the Southern Reach organisations to investigate the area.

During our bookclub meeting, we did not find that questions specific to microbiology were appropriate for this novel. Instead we concentrated on wider issues, demonstrated by the suggested discussion questions below.

Questions for discussion

1. Did you like the book? What type of book is it (genre)?
2. What do you think had happened in Area X?
3. What was the expedition trying to do?
4. What characteristics of the biologist prepared her for the visit to Area X? What were the roles of the other members of the team, and why? Did you like any of the characters?
5. What were the symptoms of the biologist's infection? Does this affect the narrative? What happened to the other members of her team? What happened to previous expeditions?
6. Is there a relationship between the tower and the lighthouse? How did you visualise the Crawler?
7. The word 'Annihilation' first appears on p 124 – in what context? Does the psychologist explain anything to us/the biologist?
8. Do you think that advances in knowledge/ science (particularly ecology and microbiology) are of interest to the author?
9. Would you recommend the book? If yes, for what reason?

Joanna Verran

Bad Bugs Bookclub Reading Guide: Parasite by Mira Grant

The aim of the Bad Bugs Book Club is to get people interested in science, specifically microbiology, by reading books (novels) in which infectious disease forms some part of the story. We also try to associate books, where possible, with some other activity or event, to widen interest, and to broaden impact.

We hope to encourage others to join, to set up their own bookclub, suggest books and accompanying activities to us, and give feedback about the books that they have read (j.verran@mmu.ac.uk).

Our bookclub comprises both microbiologists and members of the general public. We felt that this would encourage some discussion on the science – accuracy, impact etc – as well as about the book.

Parasite by Mira Grant (2013) is the first in a trilogy. In the novel 83% of the population has been protected from a range of medical conditions by the use of the 'Intestinal Bodyguard'™. This is a one-pill treatment through which a tapeworm is introduced into the body. The tapeworm then becomes the vehicle for the delivery of medication.

However, scientists had carried out certain genetic modifications to the original tapeworm, which result in changes in behaviour of the infected population. The story is told mainly through the experiences of Sally Mitchell, who has many connections to key characters in the development, marketing and surveillance of Intestinal Bodyguard™.

Questions for discussion

1. Did you enjoy the book? Did you like the characters? The writing style?

2. What are your views on the underlying premise/science for the introduction of Intestinal Bodyguard^{TM}? What do you know about the 'hygiene hypothesis?

3. What was in the implants? Where is the microbiology?

4. How was 'big business' presented? What about the Scientists?

5. Would you recommend the book to science students as a learning experience?

6. There seemed to be several teams working on the outbreak – USAMRIID, SymboGen, Dr Cale and others. What were they trying to find out/do? What were the long term aims of the different teams?

7. Who was 'the baddie'?

8. Can you explain what had happened to the implants and how this was manifested?

9. At what point in the novel did you have a view on the origin of Sally Mitchell?

10. Would you read the other books in the series? Would you read the Feed series?

Joanna Verran

Bad Bugs Bookclub Reading Guide: The Lazarus Strain by Ken McClure

The aim of the Bad Bugs Book Club is to get people interested in science, specifically microbiology, by reading books (novels) in which infectious disease forms some part of the story. We also try to associate books, where possible, with some other activity or event, to widen interest, and to broaden impact.

We hope to encourage others to join, to set up their own bookclub, suggest books and accompanying activities to us, and give feedback about the books that they have read (j.verran@mmu.ac.uk).

Our bookclub comprises both microbiologists and members of the general public. We felt that this would encourage some discussion on the science – accuracy, impact etc – as well as about the book.

The Lazarus Strain by Ken McClure (2007) is one of a series of medical thriller outputs by the author. It includes animal rights activists as well as a bioterrorism element. The aim of the terrorists is to culture massive amounts of a virulent strain of influenza virus that would replace the vaccine being developed to counteract the new strain, thereby infecting extremely high numbers of people almost simultaneously.

Questions for discussion

1. Did you enjoy the book: Did you like the characters? Would you read more Ken McClure?
2. What role did animal rights activism play in the novel?
3. Was the animal experiment carried out well?
4. There is preliminary information about influenza as well as additional scientific facts in the bulk of the novel. Was this useful?
5. The focus of the novel is on vaccination – protection of populations against infectious disease. The characteristics of influenza virus strains vary from year

to year, thus the disease epidemiology and vaccine strains needs to be continually monitored. What was the particular strain described in the novel and why was it of concern?

6. The novel was published before the H1N1 influenza pandemic of 2009. Does the emergence of that strain give any additional credence to the plot of this story?
7. The new vaccine was produced hurriedly. Do you think that testing procedures would be omitted in a ‘real-life’ situation? Do you know of any instances when faulty vaccines were produced?
8. At what point did you begin to suspect that there was an infiltrator in the laboratory? Do you think that someone should have spotted the false identify (in 2007)? What procedures might be implemented for a visiting overseas scientist?
9. The 2013 thriller ‘I am Pilgrim’ by Terry Hayes focused on the replacement of a vaccine with live virus, smallpox in this case. If you have read both books, can you comment on similarities and differences in content/style etc.?
10. Do you think that Ken McClure novels could be useful adjuncts to undergraduate teaching?

Joanna Verran

Bad Bugs Book Club Reading guide: Fever Medicine by Shawn Harmon

The aim of the Bad Bugs Book Club is to get people interested in science, specifically microbiology, by reading books (novels) in which infectious disease forms some part of the story. We also try to associate books, where possible, with some other activity or event, to widen interest, and to broaden impact.

We hope to encourage others to join, to set up their own bookclub, suggest books and accompanying activities to us, and give feedback about the books that they have read (j.verran@mmu.ac.uk).

Our bookclub comprises both microbiologists and members of the general public. We felt that this would encourage some discussion on the science – accuracy, impact etc – as well as about the book.

Fever medicine (2012) is an illustrated novel written by Shawn Harmon, and illustrated by Mara Aum, Sky Chase and Kevin Allen; these four make up the Graphic and Novel Project team. The book is free to download; those downloading are asked to provide feedback. The aims of the project are outlined at the beginning of the book, along with information about its structure, and suggestions for a reading approach. Key aims are 'to encourage contemplation of and dialogue around a range of socio-ethical issues and potential medical futures'.

Questions

Question boxes aimed at offering framework questions that might structure discussions are interspersed through the text. These are colour coded, related to topics such as ethics and regenerative medicine; some provide additional scientific information.

For a bookclub meeting, some of the questions already provided in the text could be included in the discussion – although some are very sophisticated, others are usefully provocative and most are scientific/ethics-based.

1. Did you enjoy the book? What were your impressions of the format?
2. There were three artists/illustrators – could you spot them? Did you have a favourite?
3. What impression did you get of the world in which the story was set? How important was technology? Did you appreciate the Edinburgh-laden nuances?
4. There were several characters in the novel – did you have any particular favourites?
5. What was the infection that was the focus of the novel? How prevalent was it, and how could it be prevented?
6. What was the plot?
7. A sideline to the story focused on body modification/regenerative medicine. How many of the characters had utilised any sort of enhancement? There were several question boxes focused around these aspects – could you use them to enhance your discussions?
8. The aim of the project which resulted in the novel was to raise awareness about ‘a range of socio-ethical issues and potential medical futures’. Do you think this was successful?
9. Of the many issues addressed, did any particularly interest/stimulate you?
10. Did you find the ‘lessons’ in the boxes useful? Would they be useful in an educational context?
11. A book for entertainment or education?

Joanna Verran

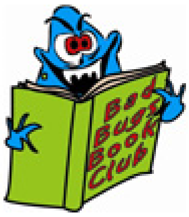

Bad Bugs Bookclub Reading Guide: Trouble with Lichen by John Wyndham

The aim of the Bad Bugs Book Club is to get people interested in science, specifically microbiology, by reading books (novels) in which infectious disease forms some part of the story. We also try to associate books, where possible, with some other activity or event, to widen interest, and to broaden impact.

We hope to encourage others to join, to set up their own bookclub, suggest books and accompanying activities to us, and give feedback about the books that they have read (j.verran@mmu.ac.uk).

Our bookclub comprises both microbiologists and members of the general public. We felt that this would encourage some discussion on the science – accuracy, impact etc – as well as about the book.

John Wyndham wrote 'Trouble with Lichen' in 1960. It was one of the stories that he called 'logical fantasy'. His novels include The Day of the Triffids, The Kraken Wakes and others. Trouble with Lichen describes the discovery of a product from a particular type of lichen which significantly slows the ageing process by up to a factor of 5, enabling people to live for 200 years or more. The two scientists who discover the 'antigerone' develop their research independently, one administering it to himself and his two adult children (without their knowledge), the other using it as part of wealthy women's beauty therapy. The consequences of the slow revelation of their discovery are described in the novel.

Questions for discussion

1. Did you like the book? Writing style? Characters? Story? Have you read any other of John Wyndham's books?
2. In what time/era do you think the story was set?

3. Do you think that this is a feminist novel? Where does Diana fit into this aspect?
4. Do you think that this is a novel about class?
5. What were the effects of consumption of anti-G on men and women?
6. The story broke ultimately as the result of an allergy claim against the beauty product. How likely are allergies to beauty products, and should the scientists have been more concerned? How much preliminary research was carried out before the product was administered? Were there any clinical trials?
7. What did you think was going to happen in the end? (what did happen in the end?)
8. Do you know of any other microbiological products used to enhance beauty/delay ageing?
9. The consequences of the pursuit of youth are explored beyond the appearance of the individual. What were the consequences, and who would be affected?
10. In one published version, the cover credits note' this is a scintillating story of the power wielded by science in our lives and asks: how much trust should we place in those we appoint to be its guardians?' Who do you think are the guardians of science?
11. What do you know about lichen?
12. What types of products are generated by microorganisms generally and lichen specifically?
13. Do think the book would be of value to the public to discuss some of the issues raised?

Joanna Verran

Bad Bugs Bookclub Reading Guide: Second Avenue Caper by Joyce Brabner

The aim of the Bad Bugs Book Club is to get people interested in science, specifically microbiology, by reading books (novels) in which infectious disease forms some part of the story. We also try to associate books, where possible, with some other activity or event, to widen interest, and to broaden impact.

We hope to encourage others to join, to set up their own bookclub, suggest books and accompanying activities to us, and give feedback about the books that they have read (j.verran@mmu.ac.uk).

Our bookclub comprises both microbiologists and members of the general public. We felt that this would encourage some discussion on the science – accuracy, impact etc – as well as about the writing.

Second Avenue Caper by Joyce Brabner (2014) is a graphic novel that describes how a group of artistic friends are affected by, and respond to, the emergence of HIV in the 1980s.

World AIDS Day is held on the 1st December each year and is an opportunity for people worldwide to unite in the fight against HIV, show their support for people living with HIV and to commemorate people who have died (information from the World AIDS Day website). World AIDS Day was the first ever global health day, held for the first time in 1988.Over 100,000 people are living with HIV in the UK. Globally there is an estimated 34 million people who have the virus. Despite the virus only being identified in 1984, more than 35 million people have died of HIV or AIDS, making it one of the most destructive pandemics in history.

World AIDS Day is important because it reminds the public and Government that HIV has not gone away – there is still a vital need to raise money, increase awareness,

fight prejudice and improve education. This year (2015), the theme is ‘think positive: rethink HIV’. The website provides many positive examples of advances that have been made, but also flags the importance of keeping HIV-AIDS in the public eye.

1. Do you know much about the graphic novel genre? What is your response to the genre? How do/did you approach reading this graphic novel?
2. Did you enjoy the book?
3. What topic do you consider to be the key focus of the novel?
4. Do you think the character portraits are well-drawn (literally and in writing)? Are they real? Does it matter?
5. Recent movies have also addressed the issue where those affected by HIV were unable to access drugs for treatment legally, and developed their own (illegal) access routes. Do you think that this action was ethically, clinically, politically, financially valid?
6. Have you seen Dallas Buyers Club (2013)? Do you see similarities in the storylines?
7. Did the drugs acquired in the stories effectively treat the infection? How does this affect the storyline?
8. Many stories and movies about AIDS now have a historical context. Do you think that these are dated and irrelevant, or that they have significant value in conveying messages about HIV-AIDS and other infectious diseases today?
9. The first World AIDS Day was in 1988. Do you think that there is value in the event?
10. What groups do you consider to be currently at risk from HIV-AIDS?
11. Do you think that HIV-AIDS is less of a significant life event than it used to be?

Joanna Verran

Bad Bugs Book Club Reading Guide: The Plague by Albert Camus

The aim of the Bad Bugs Book Club is to get people interested in science, specifically microbiology, by reading books (novels) in which infectious disease forms some part of the story. We also try to associate books, where possible, with some other activity or event, to widen interest, and to broaden impact.

We hope to encourage others to join, to set up their own bookclub, suggest books and accompanying activities to us, and give feedback about the books that they have read (j.verran@mmu.ac.uk).

Our bookclub comprises both microbiologists and members of the general public. We felt that this would encourage some discussion on the science – accuracy, impact etc – as well as about the book.

'The Plague' by Albert Camus is a modern classic novel. Published in 1947, it tells the story of a plague sweeping the Algerian city of Oran. The characters in the book help to show the effects of the plague on the population.

Questions for discussion

1. Did you like the book? The characters? The writing style? Who was the narrator, and why did s/he write in third person rather than first person?

2. 'The Plague' is considered to be 'an existentialist classic'. Existentialism is 'a philosophical theory or approach that emphasizes the existence of the individual person as a free and responsible agent determining their own development through acts of the will' (Oxford Languages). How does this knowledge affect your reading of the story?

3. There is much analytical literature about Camus and the plague. What do you know about Absurdism? In the light of Absurdism, what role does religion play in the development of the story?

4. The novel is also considered to be an allegory in which the plague signifies the German occupation of France from 1940 to 1944. Were you aware of this? If not, what is your reaction? Which parts of the novel might be read differently with this knowledge?

5. The subject of the book 'the plague' clearly makes it relevant to our bookclub. Did you think that the epidemiology/bacteriology (how much was there?), was convincing? How was the infection treated or prevented? What were the isolation strategies? What factors contributed to its spread? For how long into the twentieth century did plague outbreaks extend? Is it still around?

6. Do you think that the book works on the literal as well as metaphorical level? What other diseases could be used to narrate this story in the recent or less recent past?

7. What other 'classic' books focus on the plague? Do you know of any? What about novels with a more accessible approach? Would you have preferred to read a different one (have you tried?). We have read other 'plague' books during bookclub meetings. Do you have a favourite?

Joanna Verran

Bad Bugs Book Club Reading Guide: The Girl with all the Gifts by MR Carey

The aim of the Bad Bugs Book Club is to get people interested in science, specifically microbiology, by reading books (novels) in which infectious disease forms some part of the story. We also try to associate books, where possible, with some other activity or event, to widen interest, and to broaden impact.

We hope to encourage others to join, to set up their own bookclub, suggest books and accompanying activities to us, and give feedback about the books that they have read (j.verran@mmu.ac.uk).

Our bookclub comprises both microbiologists and members of the general public. We felt that this would encourage some discussion on the science – accuracy, impact etc – as well as about the book.

The Girl with all the Gifts (2014) is a story about a fungal infection caused by *Cordyceps*, the ‘zombie fungus’ which attains pandemic status. The manifestations of infection vary with different hosts, and the novel focuses on Melanie, a bright and intelligent child, imprisoned – and studied – by military, psychological and medical personnel. However, the situation ‘gets out of hand’….

The book can also be discussed along with the game ‘The Last of Us’, where the same fungal pathogen has triggered an apocalyptic event, and survivors make their journeys across this dangerous world. There is also a movie.

Questions

1. Did you enjoy the book?
2. Did you have any favourite characters? Who had the most ‘human’ characteristics? How did Melanie’s relationships with the different characters change as the book progressed? Do the attitudes of the uninfected adults change as the book progresses?

3. 'Your compassion or my commitment to my work?' What does the book have to say about scientific enquiry?
4. How valuable/necessary is the children's education?
5. What is Caldwell's final decision about finding a cure?
6. How long has the pandemic been going on?
7. How many novels can you recall of post-apocalyptic scenarios where we focus on the 'survival' or otherwise of (a) group(s)?
8. How does the final decision, made by Melanie, relate to any other novels you have read? How do you feel about Melanie's decision? What do you think will happen to the planet and its populations?
9. Is this the first fungal bad bug we have read about? The ability to produce spores enables airborne spread, whereas in other novels, spread is attained through bite/blood/saliva Why do you think there are so few fungal bad bugs?
10. *Cordyceps* and *Ophiocordyceps* capture the imagination with regard to their ability to modify behaviour of hosts (zombie ants). This control over humans is common to both 'the last of us' game and 'the girl with all the gifts' novel: how are the infected represented in the two outputs? Do you know of any other infections that affect host behaviour?
11. Both the game and the book focus on infected/immune/partially infected children (girls). Why do you think this is necessary for the plot to progress?
12. How similar is the behaviour of the fungus in the game/book to that of real life fungi? Time to sporulation? Trigger to sporulate? Ho do you envisage the wall of fungal mycelium 'plague-flavoured candyfloss'? Why is the wall specifically located there?
13. Do you think that games can be used to discuss 'bad bugs' in the same way as novels? How useful is the novel in microbiology education, for student use?

Joanna Verran

Bad Bugs Bookclub Reading Guide: A Fierce Radiance by Lauren Belfer

The aim of the Bad Bugs Book Club is to get people interested in science, specifically microbiology, by reading books (novels) in which infectious disease forms some part of the story. We also try to associate books, where possible, with some other activity or event, to widen interest, and to broaden impact.

We hope to encourage others to join, to set up their own bookclub, suggest books and accompanying activities to us, and give feedback about the books that they have read (j.verran@mmu.ac.uk).

Our bookclub comprises both microbiologists and members of the general public. We felt that this would encourage some discussion on the science – accuracy, impact etc – as well as about the book.

A Fierce Radiance (2010) was described in USA Today as ‘a love story and murder mystery rolled into a panoramic family saga and industrial espionage thriller’. Underpinning the plot is a focus on the government-driven need for industrial production of the newly described antibiotic, penicillin, to help treat troops injured in World War 2.

A Fierce Radiance

There are so many different characters, sub-plots and stories in the novel that questions could address many different aspects. These questions focus on the scientific dimension.

1. What did you think of the book/characters/plot? There is a lot going on - was it manageable/comfortable to read?

2. The novel was set in New York during the Second World War. How well do you think it captured the atmosphere at the time, in a country we in the UK supposed to be distant from the war?

3. The book, although fairly ‘romantic’ in tone, has its prime focus on antibiotics and microbiology. How do you think these two rather different aspects meld in the novel?

4. Do you think it would be a useful book for undergraduates to read? If yes, what sort of accompanying exercises could be set?

5. How well did the book capture the impact of antibiotics on mortality and morbidity? What diseases are mentioned through the book? What is the incidence of those diseases nowadays?

6. What was the particular difficulty of mass-producing penicillin? How has that changed?

7. Would reading this novel help you to understand the significance of AMR (antimicrobial resistance) which is of concern today?

8. Were you aware of the legislative/political processes behind antibiotic discovery, particularly in terms of the development of penicillin as compared to ‘the cousins’. What do you know about ‘natural products’ not being patented?

9. Significant side effects of the blue cousin were found. What other side effects of antibiotics are you aware of?

10. Overall, do you think that the book conveyed new and useful information about ‘bad bugs’ and antibiotics?

Joanna Verran

Bad Bugs Bookclub Reading Guide: The Manchester Man by Isabella Banks

The aim of the Bad Bugs Book Club is to get people interested in science, specifically microbiology, by reading books (novels) in which infectious disease forms some part of the story. We also try to associate books, where possible, with some other activity or event, to widen interest, and to broaden impact.

We hope to encourage others to join, to set up their own bookclub, suggest books and accompanying activities to us, and give feedback about the books that they have read (j.verran@mmu.ac.uk)

The Manchester Man by Isabella Banks (1876) was set in Manchester between 1799 and 1841. It describes the fictional life of Jabez Clegg, in the context of significant 'real' events, people and places in Manchester's history. It is not particularly (if at all) concerned with infectious disease, in contrast with, for example Mrs Gaskell's Mary Barton, but it provides an opportunity for fans of the city to talk about it!

Questions

1. Did you enjoy reading the book? What particular attributes did you/did you not enjoy?
2. What do you know about the author?
3. What is your opinion of the way the different characters are portrayed? This is a Victorian novel – did it seem as such to you? How would you define a 'Victorian' novel?
4. The book is full of information and detail. How did you feel about the pace of the story?
5. We have also read Mrs Gaskell's *Mary Barton* in the bookclub. In contrast to *Mary Barton* (written in 1848, set between 1839 and 1842), there are very few references made in the *Manchester Man* to infectious disease – or illnesses in general - in the novel. Where in the book are these references made, what

are the illnesses, and why do you think there are so few? What sort of diseases do you think would have been a problem?

6. The book addresses many historical events and well-known people in Manchester’s past. Were any new to you? Did any stand out? How well were they described, and how did they affect the development of the story and the characters?
7. The streets and districts of Manchester and the surrounding countryside that are named in the novel are ‘real’. How did this affect your reading of the book? Would you like to visit the locations?
8. What other novels about Manchester are you aware of? What makes them particularly ‘Manchester’-focused? How do they compare with *Manchester Man* with regard to this focus?
9. In previous bookclub meeting, we read *The Street Philosopher*, written in 2009 and set between 1854 and 1857, focusing on the Crimean War and Queen Victoria’s visit to Manchester for the Great Exhibition. How does this novel also contribute to setting the scene in the city in Victorian times?

Joanna Verran

Bad Bugs Bookclub Reading Guide: A Journal of the Plague Year by Daniel Defoe

The aim of the Bad Bugs Book Club is to get people interested in science, specifically microbiology, by reading books (novels) in which infectious disease forms some part of the story. We also try to associate books, where possible, with some other activity or event, to widen interest, and to broaden impact.

We hope to encourage others to join, to set up their own bookclub, suggest books and accompanying activities to us, and give feedback about the books that they have read (j.verran@mmu.ac.uk).

Our bookclub comprises both microbiologists and members of the general public. We felt that this would encourage some discussion on the science – accuracy, impact etc – as well as about the book.

A Journal of the Plague Year was published in 1722. Written by Daniel Defoe, better known perhaps for Robinson Crusoe and Moll Flanders, the story described the 1665 outbreak of bubonic plague in London. The outbreak is compressed into one year, and although it provides statistical data throughout, it is nevertheless a novel, not a 'true' account.

Questions

1. Did you enjoy the book? Why/why not?
2. Anthony Burgess' introduction to some published versions of the novel is informative. He notes' the Defoe we prize is not a working journalist but a novelist whose method is that of the working journalist'. How does this impact on Journal of the Plague Year? To what extent is the format reminiscent of a journal?
3. Another quote from Burgess is ' Defoe is one of the greatest inventors of English fiction, and yet he is unhappy about inventing. The Journal is full of

little anecdotes but the narrator is often over-careful to insist that this is all hearsay, he cannot vouch for the truth of it'. To what extent do you think the novel is 'fact' or 'fiction'?

4. The novel was published in 1722. What was the understanding of the aetiology and transmission of infection at that point? What understanding of this is revealed by the narrator?
5. How important is place (London) in the novel?
6. How does human behaviour in the face of this plague match with that we have seen in other apocalyptic novels? How does the lack of a protagonist/central character affect your reading of the novel? Burgess references Camus' 'The Plague' and HG Wells' 'War of the Worlds' as comparisons. What other novels would you compare to this description of such a virulent outbreak of disease in an enclosed population?
7. Much is made of statistics in the text. What does this contribute to the narrative?
8. What other diseases were initially misdiagnosed with plague – accidentally and deliberately How well are the symptoms of plague described? Are bubonic and pneumonic plague differentiated? How are individuals affected?
9. What strategies were used to contain the spread of infection? Which were successful and which were less so? Is there any scientific underpinning to any of these strategies?
10. Of the novels we have read about the plague, which do you remember/prefer?
11. Would you recommend this book to students for discussion?

Joanna Verran

Bad Bugs Bookclub Reading Guide: The Pesthouse by Jim Crace

The aim of the Bad Bugs Book Club is to get people interested in science, specifically microbiology, by reading books (novels) in which infectious disease forms some part of the story. We also try to associate books, where possible, with some other activity or event, to widen interest, and to broaden impact.

We hope to encourage others to join, to set up their own bookclub, suggest books and accompanying activities to us, and give feedback about the books that they have read (j.verran@mmu.ac.uk).

Our bookclub comprises both microbiologists and members of the general public. We felt that this would encourage some discussion on the science – accuracy, impact etc – as well as about the book.

The Pesthouse by Jim Crace (2007) is described on its back cover as ‘a devastated America exists in an imagined future. Its technologies are forgotten, its communities have splintered and its refugees, reversing the course of history, travel eastwards in search of safety and a new start’. The story describes the journey and experiences of a man and a woman.

Questions

1. Did you enjoy the book? What particular aspects did you find good/bad?

2. The relationship between the two protagonists, Margaret and Franklin develops through the story. Do you think this is well described?

3. How far in the future do you think the story is set? What do you think happened to cause this major change in the life of America? When do you think the change event happened?

4. Where is Ferrytown? Where is Tidewater? Why was everyone making the journey from west to east of the country?

5. What do you think is across the sea?

6. Thinking about 'Journal of a Plague Year' do you see similarities in the urban landscapes? In comparison, is the Pesthouse a story of apocalypse, adaptation or of recovery?

7. A lack of knowledge, beliefs, culture, literature, art, imagination is apparent throughout the story. Events described in the story are left incomplete. Why do you think this is?

8. How much tradition (oral or otherwise) do you think would have been carried down through the generations? How would knowledge have moved between populations? Is there any evidence of religion? Apart from metal, what else remained from before the apocalypse? What do you think would have remained that is not mentioned?

9. What is the role of metal in the story?

10. Objects recur throughout the story – the coat, her treasures, blue scarf, herb pot. What function do these objects hold?

11. Since this is the 'bad bugs bookclub', where is the microbiology? What disease do you think Margaret had?

12. Thinking about 'A Fierce Radiance', a novel we have read previously, what was the impact of treatment, on infection and antimicrobial resistance.

13. What treatments or prevention strategies did they use? Were they sensible? How do you think they developed?

14. How well does this novel fit into the bad bugs bookclub catalogue?

Joanna Verran

Bad Bugs Bookclub Reading Guide:

A Lovely Way to Burn by Louise Welsh

Isolation by SJ Gardiner

The aim of the Bad Bugs Book Club is to get people interested in science, specifically microbiology, by reading books (novels) in which infectious disease forms some part of the story. We also try to associate books, where possible, with some other activity or event, to widen interest, and to broaden impact.

We hope to encourage others to join, to set up their own bookclub, suggest books and accompanying activities to us, and give feedback about the books that they have read (j.verran@mmu.ac.uk).

Our bookclub comprises both microbiologists and members of the general public. We felt that this would encourage some discussion on the science – accuracy, impact etc – as well as about the book.

A Lovely Way to Burn by Louise Welsh (2104) is the first in a trilogy describing how individuals cope with life in the UK during a significant disease outbreak caused by a virus. A Lovely Way to Burn concerns Stevie, a shopping channel host, who survives the infection and then attempts to find out the cause of her boyfriend’s death. This is a thriller set within a disease outbreak scenario.

Unusually, we considered a second novel at this meeting, which similarly embraced the thriller-within-a-disease-outbreak format. Isolation by SJ Gardiner (2016) describes an outbreak of infections caused by an unusual strain of an antibiotic resistant bacterium within an Australian hospital (Perth). Julia Sinclair, the doctor is faced with controlling the outbreak and treating the patients, whilst a serial killer is at large.

The questions below address each book separately, so you do not need to read both books. If you do, then later questions search for comparisons.

A Lovely Way to Burn

1. Did you enjoy the book? Did you engage with the characters?
2. Did you think that the significant disease outbreak was credible? Was it a pandemic? Was the disease prevalent elsewhere? Was there any microbiology/virology of note? What was the role of the prologue?
3. Where did the outbreak originate?
4. Did you get any feel for the epidemiology of the disease? The virus was named V5N6. Is this name reminiscent of any other pathogen?
5. Was Stevie a carrier?
6. The infrastructure of London rapidly deteriorated (how rapidly?). Do you think this was likely?
7. How well did the murder/parallel storyline sit within the outbreak scenario?
8. The story touched upon the ethics of and publications about clinical studies. Do you think this would be of interest in an education context?
9. Do you think that Stevie's priorities were credible?
10. Do you think that the outbreak itself provides a useful focus for student discussion according to the bookclub remit?
11. Would you read the next two books in the series?

Isolation

1. Did you enjoy the book?
2. The blurb calls 'move over Kay Scarpetta' (Patricia Cornwell' s character) because of the scientific expertise of the author. This is SJ Gardiner's first novel: did you engage with the characters, the plot and the murders?
3. Plenty of microbiology laboratory procedures are described in some detail. Do you feel that this scientific focus is well balanced alongside the murder storyline?

4. How accurate is the microbiology? What do you know about AMR (antimicrobial resistance)? MRSA and VRSA? Where did the VRSA strain originate?
5. What barrier methods are implemented to prevent the spread of VRSA in the hospital? In what ways could VRSA be transmitted to other patients? What do you know about hospital/healthcare acquired infections (HCAI)?
6. The effect of VRSA on the individual hospital patients was touched upon with varying levels of detail. Does this personal angle convey impact more effectively that stories about the destruction of populations?
7. This is one of few novels which we have discovered which addresses antimicrobial resistance (AMR). Would you recommend using this book to engage students and/or other audiences with discussion around AMR? Can you think of other storylines that might address AMR?

Comparisons

1. Which novel did you prefer, and why? What do you know about the authors?
2. Which of the novels, if either, would you use to stimulate discussion about microbiology? If neither, do you have another suggestion?
3. The protagonist in both novels is female. What are their different motivations, and how does this affect their behaviour?
4. The transmission of the infection (and the nature of the pathogens) in the two novels was very different. How does this affect the development of the storyline?

Joanna Verran

Bad Bugs Bookclub Reading Guide:

I Contain Multitudes by Ed Jong

The aim of the Bad Bugs Book Club is to get people interested in science, specifically microbiology, by reading books (novels) in which infectious disease forms some part of the story. We also try to associate books, where possible, with some other activity or event, to widen interest, and to broaden impact.

We hope to encourage others to join, to set up their own bookclub, suggest books and accompanying activities to us, and give feedback about the books that they have read (j.verran@mmu.ac.uk).

Our bookclub comprises both microbiologists and members of the general public. We felt that this would encourage some discussion on the science – accuracy, impact etc – as well as about the book.

I Contain Multitudes (2015) is a popular science book that focus on the exciting developing field which is the study of the microbiome. Ed Jong is an award-winning science writer and communicator, and this book encompasses the microbiomes of animals, insects and the environment.

Questions

1. Did you enjoy reading the book? How did you find the structure, writing style, format?
2. This is the first overtly factual narrative that the Bookclub has read: what do you think are the differences between books written for a specialist audience, including textbooks, and popular science publications?
3. The content is entirely microbiological. Were there any particular facts/stories that stood out for you?
4. Were there any 'human interest' elements in the book?

5. Did you find any of the science particularly challenging? As a scientist/non-scientist, did you find the content interesting?
6. The human microbiome is of significant current interest in the media – can you think of any particular stories of which you are aware?
7. Has the book encouraged you to consider any changes in your behaviour?
8. Do you think the book will date? If no, why not; if yes, how could it be used in the future?
9. Do you think the general public should be more aware of the human microbiome? If yes, why; if no, why not? How could the content be modified for different audiences (children, older adults...the ‘uninterested/interested/engaged’)?
10. Would you recommend this book to students (of what age)? What activities might you use with students (or other audiences) to accompany reading of the book?
11. Would you recommend this book to future Bad Bugs Bookclubbers? To other bookclubs?

Joanna Verran

Bad Bugs Bookclub Reading Guide: A Prayer for the Dying by Stewart O'Nan

The aim of the Bad Bugs Book Club is to get people interested in science, specifically microbiology, by reading books (novels) in which infectious disease forms some part of the story. We also try to associate books, where possible, with some other activity or event, to widen interest, and to broaden impact.

We hope to encourage others to join, to set up their own bookclub, suggest books and accompanying activities to us, and give feedback about the books that they have read (j.verran@mmu.ac.uk).

Our bookclub comprises both microbiologists and members of the general public. We felt that this would encourage some discussion on the science – accuracy, impact etc – as well as about the book.

A Prayer for the Dying (1999) is set in a small Wisconsin town after the American Civil War. It describes a virulent outbreak of diphtheria, which forces self-imposed quarantine that is challenged by a forest fire sweeping the region. The local pastor, undertaker and sheriff tasks himself with supporting the inhabitants through these severe challenges.

Questions

1. Did you enjoy the book? Pace? Characters? Narrative style? Second person? Present tense? Language?
2. The book is described as 'a deeply unsettling and sophisticated horror story...an exploration of evil and solipsism'. What is solipsism, and how is it relevant to the story, in terms of content and writing style? Where do you think the horror in the story lies?
3. Did you think that the location/environment/town was well drawn? How do the isolated communities communicate with one another? The author acknowledges Michael Lesy's Wisconsin Death Trip as inspiration: why is this?

4. Are there any similarities to any other books we/you have read?
5. The book describes an outbreak of diphtheria. What do you know about diphtheria? How well does the spread of the outbreak resonate with any outbreak of diphtheria that you might be aware of?
6. What is the transmission route of diphtheria? What are the symptoms? Do the symptoms described in the book sound like diphtheria? What are the hosts for diphtheria? In the novel, the infection is 100% lethal. Do you know of any diseases that are 100% lethal? What is the mortality rate for diphtheria in ‘real life’?
7. What strategies were employed to limit the spread of diphtheria? What external pressures made these strategies difficult? How well does Jacob control cross-infection risks?
8. Is this book about an outbreak of diphtheria? What other underlying narratives are evident? The book quotes Albert Camus:’ there is no escape in a time of plague. We must choose to either love or to hate God’. Why did O’Nan choose this quote.
9. Would you recommend this book to students to learn about diphtheria? Or the spread of any other disease? How is diphtheria controlled or prevented now?

Joanna Verran

Bad Bugs Bookclub Reading Guide: The Stand by Stephen King

The aim of the Bad Bugs Book Club is to get people interested in science, specifically microbiology, by reading books (novels) in which infectious disease forms some part of the story. We also try to associate books, where possible, with some other activity or event, to widen interest, and to broaden impact.

We hope to encourage others to join, to set up their own bookclub, suggest books and accompanying activities to us, and give feedback about the books that they have read (j.verran@mmu.ac.uk).

Our bookclub comprises both microbiologists and members of the general public. We felt that this would encourage some discussion on the science – accuracy, impact etc – as well as about the book.

The Stand by Stephen King (1990 version) is a post-apocalyptic horror/fantasy novel. It outlines the total breakdown of society after the accidental release of a strain of influenza that has been modified for biological warfare, which kills off the majority of the world's human population. It tells the story of a number of survivors, and a subsequent battle between 'good' and 'evil'.

Questions

1. This is by far the longest book we have read. Were you comfortable with the length, and did you feel that the length justified the content?
2. The book has been updated three times: did you spot any references to popular culture that placed the book in a particular timeframe?
3. Stephen King's early novels focused on ordinary people ruined or damaged by extraordinary situations. Here the ordinary people of the entire world are affected. There are many characters in the novel. Did you enjoy this large cast list? Did you have a favourite character? Did the fate of any of the characters surprise you?
4. Who was the baddie?

5. There were Biblical aspects to the ‘good’ and ‘evil’ elements of the story. How did the two populations behave when establishing their different ‘civilisations’?
6. In addition to the influenza pandemic, there was also a large nuclear explosion. Were these fears appropriate for the setting of the novel, or for when the novel was originally written?
7. Did you find any microbiology? What was the disease? How did it emerge? What was its route of infection, infection rate and mortality rate? The variation in these characteristics can affect the number and behaviour of survivors and uninfected described in many novels where an influenza pandemic has occurred (eg Louise Welsh - A Lovely Way to Burn; Emily St John Mandel - Station Eleven; John Ironmonger – Not Forgetting the Whale; Taylor Antrim – Immunity; Thomas Mullen – Last Town on Earth).
8. In addition to humans, what animals were/were not affected? Certain key animals were affected – how did this affect the behaviour of the immune population?
9. Would the influenza continue (ie. would it affect any babies born to survivors)?
10. Why do you think that influenza captures the world’s interest? What current influenza stories are happening?
11. There are several novels which we have read, and others we haven’t, of apocalyptic events that decimate the world’s population. Some are ‘real-world’, and others are very much science fiction, some using vampire-or zombie-like infectious agents. Can you think of any, and do you see any similarities with The Stand?
12. Do you think the infectious disease pandemic and aftermath story remains a rich vein for authors to explore?

Joanna Verran

Bad Bugs Bookclub Reading Guide: The Citadel by AJ Cronin

The aim of the Bad Bugs Book Club is to get people interested in science, specifically microbiology, by reading books (novels) in which infectious disease forms some part of the story. We also try to associate books, where possible, with some other activity or event, to widen interest, and to broaden impact.

We hope to encourage others to join, to set up their own bookclub, suggest books and accompanying activities to us, and give feedback about the books that they have read (j.verran@mmu.ac.uk).

Our bookclub comprises both microbiologists and members of the general public. We felt that this would encourage some discussion on the science – accuracy, impact etc – as well as about the book.

The Citadel by AJ Cronin (1937) tells the story of the career and home life of Andrew Manson, an idealistic and temperamental young doctor, from his first work in a Welsh mining town, to his own practice, and finally – via various enlightening encounters that are not all positive - to a clinic that he sets up with his friends. Throughout the novel, a number of different patient cases are described, but the novel particularly highlights the difficulties of working in the pre-NHS era, and is thought to have directly influenced the foundation of the NHS. (There is also a classic 1938 movie.)

Questions

1. The book was written in 1937. Did you feel that the writing style reflected the time in which it was written? How well do you think the characters were drawn? Did the romance weaken or strengthen the novel?
2. The book was really popular. Is it appropriate to describe AJ Cronin as 'the JK Rowling of his day?'
3. The book begins with a description of general practice in the South Wales valleys. It is deemed to be hugely influential, addressing aspects of medical

ethics and laying the ground for the NHS (the film [1938] was re-released in 1948 when the NHS was established). What issues does the novel raise that are addressed by the NHS?

4. What do you know about the NHS and its original concepts? How well do you think the NHS currently copes with the demands placed upon it? How is general health in the Welsh valleys at present?
5. At what points does the book address/anticipate:
    a. Medical ethics
    b. Evidence-based medicine
    c. Continuing medical education
6. Is any of the book based on 'real' people/experiences?
7. Several infectious diseases are described in the novel – what are they, and in what context does Andrew address/treat/describe them?
8. Antibiotics were yet to be discovered/introduced. What sort of treatments were used in the novel, and how were they susceptible to immoral practices?
9. What factors contributed to making Andrew described, rather ironically as being 'well on the way to becoming an admirable honorary physician'? What finally makes Andrew 'see sense'?
10. Towards the end of the novel, Andrew defends his collaboration with the non-medically qualified Stillman by reference to Pasteur, Ehrlich, Haffkine, Metchnikoff. Why does he use these examples? Who were they and what did they do?
11. Do you think The Citadel is a useful book for discussion with students – or any other particular groups of people?

Joanna Verran

Bad Bugs Bookclub Reading Guide: Frog Music by Emma Donoghue

The aim of the Bad Bugs Book Club is to get people interested in science, specifically microbiology, by reading books (novels) in which infectious disease forms some part of the story. We also try to associate books, where possible, with some other activity or event, to widen interest, and to broaden impact.

We hope to encourage others to join, to set up their own bookclub, suggest books and accompanying activities to us, and give feedback about the books that they have read (j.verran@mmu.ac.uk).

Our bookclub comprises both microbiologists and members of the general public. We felt that this would encourage some discussion on the science – accuracy, impact etc – as well as about the book.

Frog Music by Emma Donoghue (2014) tells the story of a murder that takes place in nineteenth century San Francisco, during a heatwave when the city is also in the middle of a smallpox outbreak.

Questions

1. Why do you think the book is called Frog Music?
2. Did you enjoy the book? Were there particular aspects of the story that you found interesting? What was your opinion of the characters? (and their back stories).
3. Who are the main female characters? They are predominantly self-made, successful independent women who have made different choices in how to lead their lives, as well as in terms of their relationship with men. How does the novel address the roles of women?

4. Smallpox is mentioned in the sleeve notes as a contextualising feature of the novel (alongside the heat and the date, 1876). Do you think the novel captures San Francisco in this period?
5. The novel focuses on French and Chinese communities. What do you know of these communities in the United States (and San Francisco) in the nineteenth century – and now?
6. Donoghue was inspired by a real event to write this story. When did you discover this fact, and did it affect your reading of the novel?
7. How important is smallpox to the plot? Where does it feature? If smallpox were absent from the novel, how would the plot be affected? Is it a 'plot device'?
8. What scientific/medical aspects of smallpox are mentioned? Symptoms? Survival rates? Vaccination? Prevention? Management?
9. What do you know about smallpox?
10. Smallpox is one of the few diseases that has been declared eradicated globally. When was this declaration made? Was smallpox ever a problem in Great Britain?
11. When did the last person in the world die of smallpox? Is there any smallpox virus left in the world?
12. We previously read 'Code Orange', a 'teen novel' that described a school student's project on smallpox and his exposure to some potentially contaminated material (it was not contaminated). Code Orange seemed to be overtly educational, providing much information about smallpox. I am Pilgrim (a thriller which we have not read) describes a bioterrorist's attempts to develop smallpox as a weapon. Frog Music uses smallpox as a background to the story. Are you aware of any novels/stories that would acquaint the readership with smallpox in a more informative (than Frog Music), less clunky (Code Orange) or briefer (I am Pilgrim) style?

Joanna Verran

Bad Bugs Bookclub Reading Guide: The Last Days of Smallpox by Mark Pallen

The aim of the Bad Bugs Book Club is to get people interested in science, specifically microbiology, by reading books (novels) in which infectious disease forms some part of the story. We also try to associate books, where possible, with some other activity or event, to widen interest, and to broaden impact.

We hope to encourage others to join, to set up their own bookclub, suggest books and accompanying activities to us, and give feedback about the books that they have read (j.verran@mmu.ac.uk).

Our bookclub comprises both microbiologists and members of the general public. We felt that this would encourage some discussion on the science – accuracy, impact etc – as well as about the book.

The last days of smallpox by Mark Pallen (2018) tells the story of the 1978 Birmingham smallpox outbreak using interviews, records and other sources of information. Mark Pallen is Professor of Microbial Genetics at the University of East Anglia, and a Research Leader at the Quadram Institute in Norwich. He worked from 2001 for 12 years at the University of Birmingham, where the outbreak occurred.

Questions

1. Did you enjoy reading the book? Did you like the structure?

2. The author of Last Days of Smallpox is an active research scientist. Did you like the writing style in comparison with other books we have read written by scientists, science journalists or non-scientists?
3. How important is it for scientists to communicate to public audiences about their work?

4. The book was self-published. What do you think are the advantages and disadvantages of this?

5. Did you read about the trial or take the option to skip it? Why? Do you think the trial was carried out fairly?

6. Were the different characters well drawn? We have previously read books about ‘real’ scientists. How many of the scientists had you heard of previously?

7. Did you know about the Birmingham smallpox outbreak? Were any particular aspects surprising or shocking? How many people actually caught smallpox? How many people died?

8. Did you learn much about smallpox? Were there any particularly aspects that you were unaware of?

9. How was smallpox eradicated?

10. Do you think there are any smallpox stocks remaining in the world? Smallpox has been noted to be a potential bioterrorism agent. Why is this?

11. How do you think the outbreak occurred? Is there any historical evidence of disease (any disease) being spread through air ducts?

12. What were the repercussions of the smallpox outbreak in microbiology laboratories in terms of health and safety procedures? In terms of the use of smallpox virus in laboratories?

13. Would you recommend this book for students to read? What particular aspects would be of interest or value to students?

Joanna Verran

We were joined by the author, online and continued the discussion:

1. *What compelled you to write the book? How did you manage to fit this in with your day job?*
2. *Why did you self-publish/develop that particular structure (narrative, personal perspective)? Why did you give the reader the option to omit the court case?*
3. *Why did you decide to write in the present tense?*
4. *Your views on the importance of good science communication?*
5. *How much research was necessary? Was it easy to get interviews with key characters? Were you surprised about what you found out?*
6. *Do you have any comments on the health and safety issues raised by the case?*
7. *We all liked the final pages that actually told a more credible story about how the outbreak began. How soon in your research did you decide that this was a more likely scenario?*

Bad Bugs Bookclub Reading Guide: The Last Man by Mary Shelley

The aim of the Bad Bugs Book Club is to get people interested in science, specifically microbiology, by reading books (novels) in which infectious disease forms some part of the story. We also try to associate books, where possible, with some other activity or event, to widen interest, and to broaden impact.

We hope to encourage others to join, to set up their own bookclub, suggest books and accompanying activities to us, and give feedback about the books that they have read (j.verran@mmu.ac.uk).

Our bookclub comprises both microbiologists and members of the general public. We felt that this would encourage some discussion on the science – accuracy, impact etc – as well as about the book.

The Last Man by Mary Shelley was written in 1826. It is believed to be the first novel written about apocalyptic disease. Shelley is also attributed as having created science fiction (Frankenstein). Based in the 21st century, The Last Man describes how a global pandemic of 'plague' affects a group of people whom we meet in the first half of the novel – in the context of wider political movements, war, travel – and death.

Questions

1. The reason we selected this book (apart from the obvious) is because it is 200 years since Mary Shelley wrote Frankenstein. Have you read Frankenstein? If yes, how does this book compare?
2. Much has been written about Mary Shelley – her parents, upbringing, acquaintances with key thinkers of the time, relationship with Percy Shelley and Byron, bereavement, tragedy, loss and death. Did you read any background information out of curiosity about her? The impact of these

experiences on her life is deemed to be reflected in her works. Frankenstein is the most widely studied and highly regarded of her outputs. How much of these experiences are captured in the Last Man?

3. Did you enjoy reading the book? Were you anticipating the impact of the pandemic? When did it arrive? How did the two parts of the novel integrate with one another?
4. Did you like the characters? It is said that Raymond was based on Byron; Adrian on Percy Shelley, with Mary Shelley herself also inspiring Lionel and Idris. Does this affect your reading? How were women portrayed in the novel (and in Frankenstein)?
5. The narrative begins around 2073. Considering the significant scientific aspects to Frankenstein, what attention did Mary pay to any futuristic notions of life in the 21st century?
6. Were there any contemporary political influences on the story? Do any of the political issues described resonate today?
7. Plague first gets significant attention in Constantinople. How did the disease spread? How many years passed before the story finished?
8. Was there any useful information about the nature of the plague? What was known about microbiology at the time of writing? How was the disease controlled? Treated? What were the symptoms? Seasonality? Incubation period? How was it transmitted? What was its virulence? It is noted that the disease is ‘not what is commonly called contagious like scarlet fever or extinct (?) smallpox…It was called an epidemic’.
9. The plague swept inexorably across the continent (and had also affected the Americas). How do the black sun, and the storm, affect the narrative? Why was England seen as a refuge? What was the motivation for the few survivors to leave England?
10. The key characters of the story remain alive for much of the narrative. The final part of the story describes travels across Europe. Finally, Lionel is left alone to write his memoirs, believing himself to be the Last Man (unless there are a couple more isolated individuals). Why did he write so much about the pre-plague era?
11. This novel is the first to tell of an apocalyptic pandemic – before knowledge of microbiology. It is probably the most gloomy – can you think of another

pandemic during which everyone died? How successful is the novel? (and how successful was it at the time of publication?)

Joanna Verran

Bad Bugs Bookclub Meeting Report: Aurora by Kim Stanley Robinson

The aim of the Bad Bugs Book Club is to get people interested in science, specifically microbiology, by reading books (novels) in which infectious disease forms some part of the story. We also try to associate books, where possible, with some other activity or event, to widen interest, and to broaden impact.

We hope to encourage others to join, to set up their own bookclub, suggest books and accompanying activities to us, and give feedback about the books that they have read (j.verran@mmu.ac.uk).

Our bookclub comprises both microbiologists and members of the general public. We felt that this would encourage some discussion on the science – accuracy, impact etc. – as well as about the book.

Aurora by Kim Stanley Robinson (2015) describes the voyage of 2000 people from Earth to colonise a new planet/moon. The journey will take almost two centuries. The role of microorganisms in the success or otherwise of this venture is key to the plot.

Questions

1. Did you enjoy the book? Did your views on the book/writing style/story alter as the book progressed? Would you read any more of this author? Would you recommend the book to students?
2. Technology provided an underpinning necessity for the ship's progress. How did you react to the technical sections?
3. There were many characters in the novel. Did you have any favourites? The computer itself developed as a character – how was this conveyed? Who is/are the narrator(s)?
4. Timescales and distances are vast in space. Do you think that this was addressed successfully? Did the pace vary in different sections? How do you think the travellers view time?
5. How did the pioneers cope with the new conditions on Aurora? How would you have coped? Do you think some of the environmental conditions were unexpected?
6. Although the ship is very large, with many different 'countries' and ecosystems opportunities for travel within, was your sense of confinement

affected? How did the ship’s survivors cope when they finally returned to Earth?

7. How good was the relationship of the ship and its population with Earth/Terra, as they were travelling and as they returned to Earth? Do you think they were treated well/sympathetically? How had Earth developed in their absence?
8. Microbiology doesn’t really play much of a part initially, although it is said that ’the bacteria are evolving faster than the big animals and plants and it’s making the whole ship sick.’ Was this an early warning, or just a comment?
9. The fatal problem on Hvalsey was infection by a prion-like agent. Are you happy with the way the pathogenesis and identity of the pathogen was described? What was the incubation period? Transmission route?
10. After the failed colonisation attempt, Freya split the ship, causing a significant division between opinions as to what to do next. How would you have voted (leave/return or remain)?
11. What is co-devolution? When and why did it happen on the ship?
12. The different types of microorganisms encompass bacteria, viruses, fungi, algae and protozoa. How many of these types of microorganisms are mentioned in the novel?
13. Much research is conducted nowadays on ‘biofilms’ – communities of microorganisms (usually bacteria) attached to a surface. Is there any evidence of biofilms in the novel?
14. Also relatively unusually in our reading, it is plant pathogens rather than human pathogens which cause major problems in the ship. How many different diseases are mentioned?
15. What role does water play in the novel? Why do you think Freya’s swim marked the end of the novel?

Joanna Verran

Bad Bugs Bookclub Reading Guide: The Samurai's Garden by Gail Tsukiyama

The aim of the Bad Bugs Book Club is to get people interested in science, specifically microbiology, by reading books (novels) in which infectious disease forms some part of the story. We also try to associate books, where possible, with some other activity or event, to widen interest, and to broaden impact.

We hope to encourage others to join, to set up their own bookclub, suggest books and accompanying activities to us, and give feedback about the books that they have read (j.verran@mmu.ac.uk).

Our bookclub comprises both microbiologists and members of the general public. We felt that this would encourage some discussion on the science – accuracy, impact etc. – as well as about the book.

The Samurai's Garden by Gail Tsukiyama was written in 1994. It tells the story of 20-year-old Stephen (Chinese, but with an English name) who is sent from Hong Kong to stay in his family's summerhouse in Japan, as he recovers from tuberculosis. He develops relationships with many people who live in the tourist village through the year of his stay, particularly with his family's housekeeper, and his friend Sachi, a 'leper' who lives in isolation with other 'lepers' in a colony that is not far from the town.

Questions:

1. Did you enjoy the book? Did you have any favourite characters? How are tenses used? Does the plot reflect the seasons of the year?
2. Did you do any research on any topics covered in the book? If yes, what were the topics?
3. Did you think that Japanese culture was well-described? Was it as you might have expected? (what did you expect?)
4. How does art, and the art of gardening influence the story? How does it impinge on the relationship between Matsu and Stephen? Who is the Samurai

(what is a Samurai?) How do Sachi and Matsu's gardens compare? What roles do fire and flood play in the plot?

5. Were you aware of the Sino-Japanese war? How did the war affect the development of the plot? How do the Japanese as enemy affect Stephen's attitude to the people of Tarumi, or theirs to him (Chinese)? When did Britain gain control of Hong Kong? How did World War 2 relate to the Sino-Japanese war?
6. Where is Tarumi? Did you look on a map? What does the location of Tarumi bring to the plot?
7. Is Stephen changed by his stay in Tarumi? What about the people he meets there?

The bookclub group noted that microbiology played a significant role in the story, and suggested that students might be able to carry out project work investigating the two diseases in more depth. The following questions can be addressed by non-microbiologists, but can also be used as research prompts for students:

8. Stephen was sent to Tarumi because he was recovering from tuberculosis. At the start of the novel, what are his symptoms, and how was he treated?
9. What do you know about tuberculosis? Symptoms? Prevention? Treatment? How have things changed in terms of TB epidemiology since the Second World War? We read another book about tuberculosis (The Air we Breathe by Andrea Barrett). Can you recall the role of the disease in that novel?
10. Tuberculosis is related to leprosy. Do you know how? Stephen is taken to Yamaguchi 'so he would know he is not alone'. Is this in terms of disease, or socialisation?
11. The main focus in terms of infectious disease is leprosy rather than tuberculosis. This ancient disease is renowned for the disfigurement it causes to sufferers, and how lepers were treated. Is 'leper' an acceptable term? What do you know about leper colonies? How did the Yamaguchi leper colony first develop? Was there any treatment for the lepers? What do you know about leprosy? Did we have leprosy in England? We also read The Island by Victoria Hislop, which addressed leprosy: when was that set, and how do the outcomes differ? What is the current global status of leprosy?

12. What are the symptoms of leprosy? Are there any similarities to those of tuberculosis? How are symptoms described in the book? The disease is known for not being easy to pass on ‘couldn’t be spread by simple touch’ – yet it spread like wildfire in the story. Why might this be?
13. Antibiotics transformed the treatment of tuberculosis and leprosy. How successful is antibiotic treatment nowadays? (We read A Fierce Radiance by Lauren Belfer which described the impact of antibiotics on disease at the end of World War 2).

Joanna Verran

Bad Bugs Bookclub Reading Guide:
The Death of Grass by John Christopher

The aim of the Bad Bugs Book Club is to get people interested in science, specifically microbiology, by reading books (novels) in which infectious disease forms some part of the story. We also try to associate books, where possible, with some other activity or event, to widen interest, and to broaden impact.

We hope to encourage others to join, to set up their own bookclub, suggest books and accompanying activities to us, and give feedback about the books that they have read (j.verran@mmu.ac.uk).

Our bookclub comprises both microbiologists and members of the general public. We felt that this would encourage some discussion on the science – accuracy, impact etc. – as well as about the book.

The Death of Grass (1956) by John Christopher is a post-apocalyptic novel in which the pathogen is a virus that attacks grass. The novel focuses on a small group of English people who escape from London and journey towards a protected and isolated farmland valley where they hope to start a new and self-sufficient life. However, the behaviours of the group, and of those they meet, reveal a 'descent into barbarism'. The 2009 Penguin Modern Classics version of the book has an excellent introduction by Robert Macfarlane. The website *deathofgrass.com* provides more information about the novel and its author, as well as a podcast discussion by Matthew Jones and Ben Taylorson addressing both the book and the 1970 film version.

Questions

1. Did you enjoy reading the book? Did it remind you of any novels we had read before? The book has been likened to Lord of the Flies, and Day of the Triffids – why do you think this is?
2. The book begins in a 'green and pleasant land', and the landscape gradually changes as the virus wreaks its damage. What would a world without grass look like?

3. The novel focused on a small group of characters that enlarged as the story progressed. Did you feel that the characters were well drawn? In some cases, there were significant changes in their individual behaviours. What are your reactions to these changes? Who would you have liked to lead you?
4. The book was written in 1956. Do you feel that it was of its time, or can we learn from it today? What were key concerns of the 1950s?
5. How are women treated/presented in the novel? How is the rest of the world viewed by our protagonists?
6. The relationship between the brothers John and David is likened (by John) to the story of Cain and Abel. Do you agree with this analogy? What else could the valley represent?
7. The core of the book is a journey through which many of the characters are changed/revealed. The journey is a common plot device: can you think of any books we have read in the bookclub, or any others you know?
8. Do the issues addressed resonate with anything happening today, in agriculture, politics, etc.? Are you aware of any historic examples of similar ecological/agricultural disasters? Do you know any significant crop diseases?
9. What changes have taken place in agricultural practice since the 1950s? How might this affect susceptibility to a new disease affecting crops, soil, livestock or ‘nature’?
10. The epidemic was caused by Chung-Li virus. How was it spread? What hope is there for the USA ? What control strategies were used?
11. If the book were written today, how might it be different? What disease/crop/target might be affected?
12. How do you think the group will fare in the future?

Joanna Verran

Bad Bugs Bookclub Reading Guide: Station Eleven by Emily St. John Mandel

The aim of the Bad Bugs Book Club is to get people interested in science, specifically microbiology, by reading books (novels) in which infectious disease forms some part of the story. We also try to associate books, where possible, with some other activity or event, to widen interest, and to broaden impact.

We hope to encourage others to join, to set up their own bookclub, suggest books and accompanying activities to us, and give feedback about the books that they have read (j.verran@mmu.ac.uk).

Our bookclub comprises both microbiologists and members of the general public. We felt that this would encourage some discussion on the science – accuracy, impact etc. – as well as about the book.

Emily St. John Mandel's book Station Eleven (2014) is a post-apocalyptic narrative describing events up to twenty years after an influenza pandemic that killed everyone it infected: there are few survivors. It is based in Canada. The story follows a number of people (and objects) through the pandemic: the link between them is Arthur Leander, an actor.

Questions

1. Did you enjoy reading the book? What particular aspects affected you?
2. Does the novel have a main character?
3. What roles do objects play in the story?
4. Is it better or worse to remember the time before the flu? What would you remember?
5. These questions are adapted from those posed at the end of the book (Picador, 2014):
   a. How do Shakespearean motifs coincide with those of Station Eleven, both the novel and the comic?
   b. What is the metaphor of the Station Eleven comic books?
   c. How does the Undersea connect to the events of the novel?

6. Jeevan seemed very content with his world; where do you think is the best community? If Jeevan had been able to save Arthur, how would the trajectory of the novel have been different (second part of the question is adapted from questions provided in the book)?
7. The Georgia Flu is the pandemic that brings about the end of the world as it was known. It appears to arrive in Canada on a plane. What particular issues affect disease spread during flight? What other epidemics have been associated with spread by flight? It was also noted that 'Georgian and Russian officials' were 'somewhat less than transparent about the severity of the crisis there'. Does this resonate with any more recent events?
8. What do you know about influenza virus and influenza outbreaks, epidemics and pandemics? Have you ever had influenza?
9. What was the epidemiology of this pandemic?
    a. How infectious?
    b. How long an incubation period?
    c. How lethal?
    d. Any immunity? Any recovery?
    e. Symptoms?
10. The Travelling Symphony enables a journey across the country to be described, and other settlements to be encountered. What other novels do you know with 'journeys' as a plot device?
11. There are many novels written about influenza, and the plot varies depending on the virulence of the pandemic – various clues are given about the properties of the virus. Do you know of any? Can this be related to our knowledge of 20th century influenza outbreaks?
12. 2019 marks the centenary of the influenza pandemic that accompanied and followed World War 1. Do you think the public are sufficiently aware of this global catastrophe? Is there anything we could learn from it?
13. Could you use fiction to engage students with influenza?

Joanna Verran

Bad Bugs Bookclub Reading Guide:
The Killing Snows by Charles Egan

The aim of the Bad Bugs Book Club is to get people interested in science, specifically microbiology, by reading books (novels) in which infectious disease forms some part of the story. We also try to associate books, where possible, with some other activity or event, to widen interest, and to broaden impact.

We hope to encourage others to join, to set up their own bookclub, suggest books and accompanying activities to us, and give feedback about the books that they have read (j.verran@mmu.ac.uk).

Our bookclub comprises both microbiologists and members of the general public. We felt that this would encourage some discussion on the science – accuracy, impact etc. – as well as about the book.

The Killing Snows (2012) is the first of a trilogy by Charles Egan. It is a fictional account of a family during the Irish potato blight and Great Famine, inspired by the author's family history research, and his interest in Irish history and migration. The Killing Snows is based in County Mayo.

Questions

1. Did you enjoy the book? Writing style, plot, characters?
2. Did you feel that it gave a good description of rural life in Ireland (before the blight/famine?). Would you read the next two novels in the series?
3. The trigger for the famine was potato blight. What do you know about this infection? Why did it have such an impact in Ireland? What is the pathogen? What was available to eat instead of potatoes?
4. Is potato blight still a problem? How is it controlled/prevented/treated?
5. By 1846, and even before the potato blight, many Irish were 'navvies' in Britain, involved in a range of significant building projects. Are you aware of any local projects that utilised the Irish workforce? Where is Box Tunnel?

6. In the novel, there are links between Liverpool, Manchester (Stockport) and Ireland. What are these links and why are they so important? What role did the ports have in Ireland?
7. How did Danny make money out of the Irish immigrants? Do you think Danny was a good businessman?
8. What was the Rebellion in 1798? Were any other historical events referenced?
9. What strategies were used to feed the people (rock breaking, road building, workhouses, soup kitchens)? Where did food come from? What was the food?
10. Luke gets a valuable but difficult job providing employment in his quarry/on the roadworks. What is ‘selection’? How did the work affect his mental health?
11. How do you think the Great Famine has impacted on Ireland today? Its population, landscapes, communities, villages, cemeteries?
12. Black fever – what is it and how is it spread?
13. Many different symptoms are described. What are they? Are they all due to one disease? What were the ‘fox children’?
14. Of the various pressures on the Irish poor, what do you think were the worst in terms of causes of death?
15. What role did religion play in the story?
16. We have also read Star of the Sea – how does the books compare in terms of content/storyline?

Joanna Verran

Bad Bugs Bookclub Reading Guide: The Health of Strangers by Lesley Kelly

The aim of the Bad Bugs Book Club is to get people interested in science, specifically microbiology, by reading books (novels) in which infectious disease forms some part of the story. We also try to associate books, where possible, with some other activity or event, to widen interest, and to broaden impact.

We hope to encourage others to join, to set up their own bookclub, suggest books and accompanying activities to us, and give feedback about the books that they have read (j.verran@mmu.ac.uk).

Our bookclub comprises both microbiologists and members of the general public. We felt that this would encourage some discussion on the science – accuracy, impact etc. – as well as about the book.

The Health of Strangers (2017) by Lesley Kelly is the first in a series of novels focusing on the work of the North Edinburgh Health Enforcement Team (HET), which was created as part of the response to the Virus – a new pandemic of H1N1 influenza. Set in current times, the HET follows up on individuals who fail to turn up for their regular health checks, encountering a range of people and organisations whose activities are connected to the Virus.

Questions

1. Did you enjoy the book? Were the characters well drawn?
2. Typically, ‘place’ is key to plot. What role, if any, does the city itself have in the plot? What other novels do you know about where Edinburgh provides the setting?
3. When do you think the story is set? Do you think that issues around Scottish independence or Brexit could have/should have been included?
4. Were you impressed by the state’s response to the outbreak? How might HET activities affect the epidemiology of the outbreak? What prevention strategies were in place for the populace?

5. How did ‘the virus’ affect the plot and its development? How would the story have progressed without the underpinning and ongoing disease outbreak? We have read other novels that focus on crime, but which are set within a context of a significant or potential disease outbreak (eg A Lovely Way to Burn, Unnatural Exposure). Why do you think disease outbreaks are used as a plot device?
6. What was the source/origin of the outbreak? How long had it been going on?
7. The story is set after the peak of a significant pandemic. What information could you glean about the disease epidemiology? The Virus seems to be based on the 1918 influenza pandemic strain. What similarities in the epidemiologies could you spot?
8. Religion is one of the perceived ‘solutions’ for treatment of the disease. Do you know of other novels or historical instances where this is the case? What about other ‘cures’?
9. In what context is Camus’ The Plague used?
10. What do you know about influenza? Have you had influenza? Have you been immunized?
11. The book is the first in a series featuring the same characters, but with an overarching ‘arc’ about the virus and its management. Would you read the other novels?
12. This bookclub meeting took place as part of the ESRC festival, in an event focused on ‘the city’. We have read several books about pandemics or epidemics that have been set in cities [for example Nemesis (New Jersey), The Plague (Algerian city of Oran), A Journal of the Plague Year (London), Frog Music (San Francisco), Ghost Map (London), Mary Barton (Manchester), Fever (New York)]. Can you think of any others? Why is this a popular plot device? How does the city and its populace affect the development of the plot or the spread of disease?

Joanna Verran

Questions for the author (who joined our discussion online)

1. Tell us about your book series.
2. Where did your idea come from?

3. Why did you set the book after the epidemic was essentially over?
4. How much research did you do?
5. Why did you choose influenza? Where is the series going? When is the next book out?
6. Why Edinburgh?
7. Would you put the books in a genre?

Bad Bugs Bookclub Reading Guide:
Contagion by Robin Cook

The aim of the Bad Bugs Book Club is to get people interested in science, specifically microbiology, by reading books (novels) in which infectious disease forms some part of the story. We also try to associate books, where possible, with some other activity or event, to widen interest, and to broaden impact.

We hope to encourage others to join, to set up their own bookclub, suggest books and accompanying activities to us, and give feedback about the books that they have read (j.verran@mmu.ac.uk).

Our bookclub comprises both microbiologists and members of the general public. We felt that this would encourage some discussion on the science – accuracy, impact etc. – as well as about the book.

Robin Cook (b 1940) is an American physician and writer. Cook says he chose to write thrillers because the forum gives him "an opportunity to get the public interested in things about medicine that they didn't seem to know about. I believe my books are actually teaching people”.

Contagion (1995) was selected by the bookclub because it encompassed a range of microorganisms, and a mention of AMR. The novel describes a series of nosocomial outbreaks caused by unusual and virulent pathogens. The outbreaks are eventually revealed to be caused deliberately.

For the purposes of the bookclub, we decided that Contagion could be read from two different viewpoints: as a thriller which contains a significant amount of microbiology that could stimulate significant debate and learning exercises for students – and as a novel read for enjoyment (and to meet the aims of the Bad Bugs Bookclub). For this reason, questions for discussion are divided into two sections. Of course any of the questions can be used (there is some repetition between the two sections)!

Questions for general discussion

1. Did you enjoy the book? Do you think it could be used within an undergraduate/postgraduate microbiology curriculum?
2. What do you feel are key components of a ‘thriller’? Did you feel appropriately ‘thrilled’? At what point in your reading did you identify the cause of the outbreaks?
3. The book begins by noting that three people’s lives intersected. How many clues were provided to help readers to identify these three individuals, and their ‘intersections’? How did their backstories contribute to the plot and their motivations? What was the motivation for the outbreaks?
4. Did you like the characters? How was the gender and diversity balance?
5. The diagnosis, pathology and microbiology were described in some detail throughout. As a microbiologist/non-microbiologist/non-scientist, how did you find the technical descriptions/language?
6. What is a nosocomial infection? How many nosocomial outbreaks were mentioned other than those created by the saboteurs? Are you familiar with any ‘real’ nosocomial outbreaks?
7. What did all the cases have in common? What routes of infection/contamination were considered? What infection control procedures were undertaken in each case?
8. Why are the initial outbreaks small?
9. How does Therese’s job relate to Jack’s? What does she learn from him? Did you learn anything from him?
10. The final outbreak is a viral pneumonia. What was its origin? How did the outbreak differ from the four previous ones? How many cases? How was it treated/controlled?
11. How/where were the pathogens sourced and cultivated? How were patients infected? What do you think were the locked cultures in the incubator room? What was A-81 ‘mucoid, transparent colonies on chocolate agar’? Do you think these pathogens were sensible choices to trigger nosocomial outbreaks? What would have been better pathogens to cause similar problems?
12. Why did ‘the baddies’ not kill Jack immediately?
13. What is your overall opinion of this novel as a Bad Bugs Book?

Questions for microbiology students

1. Did you enjoy reading the book?
2. What is a nosocomial infection? How many nosocomial outbreaks were mentioned other than those created by the saboteurs? Are you familiar with any 'real' nosocomial outbreaks? How does AMR fit into nosocomial infection?
3. How many outbreaks were caused by the saboteurs? Write a case history for a selected patient.
4. How many cases were there for each outbreak? What was the mortality for each outbreak? Draw a map of the outbreaks and the chain of infection.
5. How have diagnostic methods changed since the book was written? Were the laboratories/procedures described well?
6. What were the common symptoms of the outbreaks? How might this affect infection control protocols?
7. The first three outbreaks were caused by rare 'arbo' diseases. What are vectors – what additional diseases do you know that are caused by arthropod borne pathogens?
8. What do you know about plague, tularaemia and Rocky Mountain spotted fever, and how common are they? What are the diagnostic procedures? Class 3 unit? What category of pathogens are the different outbreaks caused by?
9. Treatment for tularaemia and plague? How does rickettsia differ from the other two bacteria. What is the treatment for Rocky Mountain Spotted fever?
10. The fourth outbreak is caused by meningococci. What is Waterhouse-Friedrichsen syndrome? The meningococcus is described as being transmitted through the air – why does it not have outbreak potential? What is the treatment/prophylaxis for meningococcal meningitis? Why are throat cultures taken from staff?
11. What do you know about the influenza virus, and the 1918 strain? How was it typed? Where were the different pathogens sourced?
12. Summarise the similarities and differences between the five pathogens.
13. Do you think that reading fiction can help the reader to learn about microbiology?

Joanna Verran

Bad Bugs Bookclub Reading Guide:
Fever 1793 by Laurie Halse Anderson

The aim of the Bad Bugs Book Club is to get people interested in science, specifically microbiology, by reading books (novels) in which infectious disease forms some part of the story. We also try to associate books, where possible, with some other activity or event, to widen interest, and to broaden impact.

We hope to encourage others to join, to set up their own bookclub, suggest books and accompanying activities to us, and give feedback about the books that they have read (j.verran@mmu.ac.uk).

Our bookclub comprises both microbiologists and members of the general public. We felt that this would encourage some discussion on the science – accuracy, impact etc – as well as about the book.

Fever 1793 (2000) by Laurie Halse Anderson is set in Philadelphia in 1793, during the well-known (in US) yellow fever outbreak. It focuses on the experiences of a fourteen-year-old girl across the summer period when the fever was rife, and is intended for a primarily young adult audience.

In addition, if members wanted additional more challenging reading, they were directed to the American Gothic novel Arthur Mervyn (1799) by Charles Brockden Brown, which is also set in Philadelphia in 1793.

Questions for discussion

1. Did you enjoy reading the book? It was written for young adults (quite young – how young?), so what did you get from it? It was set 200 years ago – does it feel that long ago. What was Philadelphia like then? Did you try to make the cake (recipe in the book)?
2. The story took in much of the history that framed the yellow fever outbreak. Did you spot any particular aspects of American History that you followed up?

a. Philadelphia as the capital of US. Presence of Washington and Jefferson
b. War of independence
c. The French
d. Posh houses, poor houses, coffee houses, the docks
e. The balloon event
f. Docks, slavery, immigration
g. Free blacks and prejudice

3. With regards to yellow fever, what was known regarding transmission at the time (the first line gives a hint!)? Where did people think it came from?
4. What were the prevention strategies?
5. What were the treatment strategies? Was there any Government/central administration advice? What was Bush Hill? How were bodies disposed of?
6. How do the behaviours of the population relate to those in other books we have read? Of any other diseases you know from history (eg plague).
7. What are the symptoms/incubation period/duration of illness? Can you tell when Mattie got infected?
8. What do you know about yellow fever nowadays? How is it prevented/transmitted? How was the transmission route discovered, and when?
9. Is yellow fever still a problem? What other arboviruses do you know of? For example, Zika virus and dengue belong to the same virus family as yellow fever.

Arthur Mervyn

1. Who in the group read this book? What did you think of it?
2. The book was actually written in 1799 only 6 years after the yellow fever outbreak. Did you feel that a good picture of Philadelphia was painted?
3. This is ‘American Gothic’. It appears to have inspired Mary Shelley to write The Last Man. Which of the 2 did you prefer?
4. Can you see complementary aspects of the yellow fever outbreak in the two novels?
5. What descriptions of yellow fever were there? What was the treatment?

6. The novel considers aspects of multiculturalism, and the role of women. Do you think this was successful?

General

1. Would you recommend either of these books to others? In what circumstances?
2. How useful an addition to the bookclub canon would either of these books be?

Coronavirus discussion points (this meeting took place in 2020, a week before UK lockdown):

1. Has reading fiction assisted in your understanding of pandemics?
2. Do you feel more/less/same levels of anxiety?
3. Do any of the books we have read so far present similar/different patterns from coronavirus?
4. Have you/are you taking any current action with regards to coronavirus? What are your current sources of information?

Joanna Verran

Bad Bugs Bookclub Reading Guide:

Pale Rider by Laura Spinney

The Eyes of Darkness by Dean Koontz

Pale Horse, Pale Rider by Katherine Anne Porter

The aim of the Bad Bugs Book Club is to get people interested in science, specifically microbiology, by reading books (novels) in which infectious disease forms some part of the story. We also try to associate books, where possible, with some other activity or event, to widen interest, and to broaden impact.

We hope to encourage others to join, to set up their own bookclub, suggest books and accompanying activities to us, and give feedback about the books that they have read (j.verran@mmu.ac.uk).

Our bookclub comprises both microbiologists and members of the general public. We felt that this would encourage some discussion on the science – accuracy, impact etc – as well as about the book. This was the first online meeting due to the coronavirus pandemic (all subsequent meetings took place online).

Three books were selected for discussion, mainly due to their relevance to the coronavirus pandemic: The Eyes of Darkness by Dean Koontz (1981, revised 1996) , a thriller about a lethal virus engineered in Wuhan (much hyped to have predicted the pandemic); Pale Rider: the Spanish flu and how it changed with world by Laura Spinney (2017), a non-fiction account of the 1918 influenza pandemic; and Pale Horse, Pale Rider by Katherine Anne Porter (1937), a short story about a young woman struck down with influenza just when the armistice was declared.

Questions for discussion

Eyes of darkness

1. Did you enjoy reading the book?
2. 'Did this thriller predict the coronavirus outbreak?' (Daily Mail)
3. Do you think that this is a good 'bad bugs book?'

Pale Rider

1. Did you enjoy reading the book? Did it help you reflect on the current coronavirus pandemic? Is it possible not to think about coronavirus when reading this?
2. The author, Laura Spinney has been fairly prominent in the media during the coronavirus pandemic. Why do you think this is?
3. Why is the book called 'Pale Rider?' Who originated the term in context of the 1918 pandemic?
4. 'The Spanish flu of 1918 and how it changed the world'. Why was it called Spanish flu? What other pandemics do you know of? What do their names reflect?
5. Were there any conspiracy theories focusing on influenza 1918?
6. The total estimated number of deaths from the 1918 influenza pandemic ranges from 50 million to 100 million. Why do you think there is such a huge difference?
7. What were the treatments/prevention strategies?
8. Although it is often interwoven with WW1, Spinney gives the 1918 pandemic a global perspective. Were there any countries/populations whose stories you found especially memorable?
9. We see war analogies in our current 'battle' against coronavirus. What role did the media have in reporting in the 1918 crisis (and now)?
10. Spinney provides plenty of accessible scientific information in Pale Rider. Did scientific/medical knowledge affect the progress of influenza in 1918? Has this changed over time?
11. Would you recommend this book? If yes, to whom?

Pale Horse, Pale Rider

1. Did you enjoy reading the story? (characters, writing style etc).
2. There are very few novels or short stories which focus on the 1918 influenza pandemic, and which were written contemporaneously. Spinney addresses this in her book Pale Rider. Why do you think this is?
3. How did the story contextualise influenza in Miranda’s life?
4. Miranda’s symptoms progress with the story: did you think this was effective?
5. How did you feel about the ending?
6. We have read several novels that deal with influenza, many of which have been written relatively recently. Why has influenza been such a good subject for fiction? Do you anticipate coronavirus in future fiction?

Joanna Verran

Bad Bugs Bookclub Reading Guide:

Not Forgetting the Whale by John Ironmonger

The aim of the Bad Bugs Book Club is to get people interested in science, specifically microbiology, by reading books (novels) in which infectious disease forms some part of the story. We also try to associate books, where possible, with some other activity or event, to widen interest, and to broaden impact.

We hope to encourage others to join, to set up their own bookclub, suggest books and accompanying activities to us, and give feedback about the books that they have read (j.verran@mmu.ac.uk).

Our bookclub comprises both microbiologists and members of the general public. We felt that this would encourage some discussion on the science – accuracy, impact etc – as well as about the book.

Not Forgetting the Whale by John Ironmonger (2015) describes how a small Cornish village survives an influenza pandemic, led by a young incomer from London. The author joined our discussion online.

Questions

1. Did you enjoy the book? What were your particular emotional impressions?
2. There are many different characters in the novel. Were they well drawn? Why do you think there were so many characters in St Piran? What were your impressions of the relationship between Joe Haak and Polly Hocking – and then Amanita?
3. Did you identify any particular themes in the novel?
4. Are you familiar with the south-west of Cornwall? Where would you flee before ‘getting your tyres wet’? Did you envision the village in any particular location? How likely is a small fishing village in Cornwall to have been left out of the tourist trade?

5. This pandemic was caused by an influenza virus ‘identical to the 1918 flu virus’. What was the fatality rate? How does that compare with the 1918 influenza and 2020 coronavirus pandemics, and seasonal flu? How quickly did it spread? What was the incubation period, and how soon were people non-infectious? What were the symptoms? What was the pandemic’s origin?
6. Do you see any parallels or differences between the story and the 2020 coronavirus pandemic?
7. In the novel, there are some fairly complex explanations about supply chains, the City, analysts, modelling. How successful was this for you? How does Martha’s ‘connecting dots’ relate to the modelling? What was omitted from the modelling?
8. ‘It is said that our society is three square meals from anarchy’. ‘It won’t be the disease that kills us, it will be the fear’. In the novel, there is a complete breakdown of society. This does not appear to have happened as yet in the coronavirus pandemic. Why might this be?
9. ‘Self-interest drives everything we do’. Is this demonstrated in the book?
10. How many incursions did the village experience – were you surprised at how many/how few? How did you feel about the hoarding of food?
11. There are several books/authors referenced in the novel. Did you reference/ read/have you read any of them?
    a. Authors - Francis Galton, Norman Angell, Thomas Hobbes, Jared Diamond (in author’s notes)
    b. Fiction - Day of the Triffids, The Road, I am Legend, The Last Man – all post-apocalyptic survivalist…other relevant novels have we read?
12. Would this be a good book to recommend for bad bugs bookclub/students?

    For author:
13. What gave you the initial idea?
14. How much research was necessary?
15. Where in Cornwall did you picture St Piran to be?
16. How do you feel your book stands up in the current coronavirus pandemic? Is there anything you would have changed?

Joanna Verran

Bad Bugs Bookclub Reading Guide:

The Constant Gardener by John Le Carre

The aim of the Bad Bugs Book Club is to get people interested in science, specifically microbiology, by reading books (novels) in which infectious disease forms some part of the story. We also try to associate books, where possible, with some other activity or event, to widen interest, and to broaden impact.

We hope to encourage others to join, to set up their own bookclub, suggest books and accompanying activities to us, and give feedback about the books that they have read (j.verran@mmu.ac.uk).

Our bookclub comprises both microbiologists and members of the general public. We felt that this would encourage some discussion on the science – accuracy, impact etc – as well as about the book.

The Constant Gardener (2001) by John Le Carre is set in the context of a clinical trial, for treatment of tuberculosis, taking place in Kenya. The story was also made into a film in 2005 (dir: Fernando Meirelles) starring Ralph Fiennes and Rachel Weisz. The movie was filmed in Kenya. The movie and the novel can be discussed together or separately. The subject matter is wide-ranging, allowing discussion on many topics. An expert in clinical trials was invited to the meeting, and a twitter discussion was also posted.

Questions

1. Did you enjoy the book/film? If you saw both, how did they compare?
2. The novel is written from several points of view – initially Woodrow and later Quayle (with a bit of Gloria). Did you feel that the characters were well drawn?
3. There are a lot of characters, and the plot is fairly detailed and complex – with some differences between novel and movie. How did you cope with this? Are you familiar with Le Carre's work?

4. When was the novel set? Did you get a feel for the period? Did any of the issues resonate with current times?
5. Many references were made to real events, and the history of Kenya. Did you check any of it? The movie was filmed in Kenya, including Kibera. Did you enjoy the settings and landscapes in the novel/film?
6. In the DVD version of the movie, and at the end of the book, Le Carre provides an author's note, and explains what inspired him. Why was your impression of this addendum?
7. What disease was the focus of the book? What other diseases were mentioned? What are the current leading global infections/causes of death?
8. Is TB a global problem? What are the symptoms? Why is the number of those infected with TB so high? Why is it so difficult to control?
9. What about HIV/TB dual infection? What is the current status of TB therapy? What about antimicrobial resistance?
10. Tessa is angry that TB is/was linked to the Romantic period and literature. What do you know about this, and why is she angry? What names were used historically for TB?
11. What did Dypraxa do? Who was it given to? What were the side effects? What is the context for the ethical issues? Is the science plausible?
12. Is this how a trial works? What do you know about the phases of clinical trials? What was wrong with the trials being carried out for Dypraxa?
13. What is the situation for the provision of drugs to Africa?
14. How might this novel be used in HE?

Twitter questions

1. (8.00) Welcome to the #badbugsbookclub Twitter meeting about The Constant Gardener by @lecarre_news. Six questions will be posted over the next hour. Please reference the #badbugsbookclub hashtag and question number in your responses! Keep refreshing.
2. (8.01) Introduce yourself and briefly give your overall opinion/level of enjoyment of the book and/or movie The Constant Gardener #badbugsbookclub

3. (8.10) The novel is set in the context of a clinical trial for treatment of tuberculosis in Kenya. Is/was TB a problem? #badbugsbookclub
4. (8.20) In our bookclub, we mine our novels for microbiology content. Is this novel a good example? #badbugsbookclub
5. (8.30) In the novel, Tessa is angry that TB is associated with the Romantics. Why was this? What do you know about this? What names were used for TB? #badbugsbookclub
6. (8.40) Did you learn anything about clinical trials from the novel? #badbugsbookclub
7. (8.50) Why is MDRTB such a cause for concern? How has it emerged? #badbugsbookclub
8. (9.00) Thank you everyone for participating. Check the #badbugsbookclub website for previous and forthcoming reading. Happy #InternationalMicroorganismDay!

Joanna Verran

Bad Bugs Bookclub Reading Guide:

The Waiting Rooms by Eve Smith

The aim of the Bad Bugs Book Club is to get people interested in science, specifically microbiology, by reading books (novels) in which infectious disease forms some part of the story. We also try to associate books, where possible, with some other activity or event, to widen interest, and to broaden impact.

We hope to encourage others to join, to set up their own bookclub, suggest books and accompanying activities to us, and give feedback about the books that they have read (j.verran@mmu.ac.uk).

Our bookclub comprises both microbiologists and members of the general public. We felt that this would encourage some discussion on the science – accuracy, impact etc – as well as about the book.

The Waiting Rooms by Eve Smith (2020) is set in the near future (and in the recent past), in a world beset by antibiotic resistance. The lack of effective antibiotics has had enormous repercussions on our way of life and civilisation itself. In this world, Kate searches for her birth mother. The author joined our online discussion. The meeting was held during World Antimicrobial Awareness Week, and a twitter discussion was also hosted.

Questions

1. Did you enjoy the novel? Did the characters and relationships draw you into the plot? Did you have empathy or antipathy for any of the characters? Was there any circularity with Kate and Lily? What about childbirth – how did it differently affect Lily, Cara, Kate and her daughter?
2. Do you think the representation of a contemporary world without antibiotics (or with very few) is a realistic one? Around when (what year) did the 'crisis' happen?

3. In the novel, what are the strategies for maintaining control of the few antibiotics that are effective?
4. What sort of conditions/medical/clinical procedures are affected by AMR?
5. Lily complains about the ‘ageist apartheid’. What are your views on this? The author describes how Lily’s body makes her dependent, whilst her brain remains active. Did you think this was well drawn? There are several different places where ‘the elderly’ go – what are they and how do they differ? Where are the Waiting Rooms?
6. Our previous read, The Constant Gardener, also focused on TB and Africa. What similarities are there in the two novels? Why is TB so difficult to treat? What is the current prevalence of TB?
7. Decades before the ‘crisis’ Mary and Piet were searching for alternative antimicrobials. Are you aware of any natural antimicrobials, or projects searching for them (ie not from other microorganisms). WAAR (World Antimicrobial Awareness Week) this year is focusing on antimicrobials not specifically antibiotics (hence the name change from antibiotics to antimicrobials). Why do you think this is the case?
8. Were you aware of the increasing problem of AMR prior to reading this book? Has the book made you consider any changes to your behaviour/attitude?
9. Did you look up anything as a result of reading the book?
10. In comparison to for example influenza, there have been very few novels written about AMR. Why do you think this is the case?
11. Do you think that this novel is useful in helping non-scientists to understand/discuss AMR? Would you become an Antibiotic Guardian?

Questions for the author

1. When did you start writing the book?
2. In what year did the Crisis happen?
3. Has writing the novel made you more aware of AMR? Do you keep up to date with developments? Why did you choose TB?
4. Did you consider that childbirth (and therefore family) was a theme? Most of the key characters are women.
5. Botanical names all checked out! What is your interest in botany, and Africa?

6. There were so many occasions where the novel and the coronavirus pandemic blended. We particularly enjoyed the scientist/government discussions. How did you ‘predict’ this?
7. What will your next novel be about?

Twitter Questions

1. 8.00 Welcome to our #WAAW discussion on The Waiting Rooms by @evecsmith. Please use #badbugsbookclub in your posts, and reference the question number (1-6) in your responses. You can ask questions too! First (not a question), say hi and note your interest in #AMR, and/or the novel.
2. 8.01 Q1 Do any of the actions made to control AMR in the novel resonate with those suggested/implemented for coronavirus? Do we live in an era of ‘ageist apartheid’? Is AMR an issue with coronavirus? #badbugsbookclub
3. 8.10 Q2 As an ongoing theme throughout the discussion, what questions would you like to ask @evecsmith about The Waiting Rooms and what did you particularly like or find challenging about the book? Do you have any questions for our scientists? #badbugsbookclub
4. 8.20 Q3 How does AMR emerge in the novel and in real life? #badbugsbookclub
5. 8.30 Q4 The novel describes a contemporary/near-future world where few antibiotics are effective. Were you surprised by any of the impacts? Several types of microorganisms are implicated. What do you think are currently the most important drug resistant pathogens? #badbugsbookclub
6. 8.40 Q5 The slogan for WAAW 2020 is ‘Antimicrobials: handle with care’. Why antimicrobials and not antibiotics? What is bioprospecting? #badbugsbookclub
7. 8.50 Q6 Do you think that reading fiction can be used to consider aspects of infectious disease? Do you have any further questions or comments? #badbugsbookclub
8. 9.00 Thank you. That’s all folks! Think about any behavioural changes you might make (www.AntibioticGuardian.com). Goodnight and stay safe. #badbugsbookclub

For University students (Antimicrobial Resistance is so important: the novel can provide a useful opportunity for further exploration and discussion of the topic).

Questions

1. Did you enjoy the novel? Did the characters and relationships draw you into the plot? Did you have empathy or antipathy for any of the characters? Was there any circularity with Kate and Lily's stories? What about childbirth – how did it differently affect Lily, Cara, Kate and her daughter?
2. Do you think the representation of a contemporary world without antibiotics (or with very few) is a realistic one? Around when (what year) do you think the 'crisis' happened?
3. In the novel, what are the strategies for maintaining control of the few antibiotics that are effective?
4. What sort of conditions/medical/clinical procedures are affected by AMR?
5. Lily complains about the 'ageist apartheid'. What are your views on this?
6. The author describes how Lily's body makes her dependent, whilst her brain remains active. Did you think this was well drawn? How old was she?
7. There are several different places where 'the elderly' go – what are they and how do they differ? Where are the Waiting Rooms?
8. What reasons are given for the emergence of antibiotic resistance?
9. Why is TB so difficult to treat? What is the current prevalence of TB?
10. Decades before the 'crisis' Mary and Piet were searching for alternative antimicrobials. Are you aware of any searches for natural antimicrobials? WAAR this year is focusing on antimicrobials not specifically antibiotics: what is the difference between antibiotics and antimicrobials?
11. Several infections/conditions were noted that resulted from AMR, and several bacterial pathogens were described. For you, what are the most important drug resistant pathogens currently?
12. Were you aware of the increasing problem of AMR prior to reading this book? Has the book made you consider any changes to your behaviour/attitude?
13. Did you look up anything as a result of reading the book? Did you read the author's blog?
14. Do you see any parallels or relationship with the current coronavirus pandemic?

15. In comparison to for example influenza, there have been very few novels written about AMR. Why do you think this is the case?
16. Do you think that this novel is useful in helping non-scientists to understand/discuss AMR? Would you become an antibiotic Guardian?

Joanna Verran

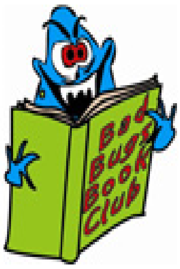

Bad Bugs Bookclub Reading Guide:

Little Women by Louisa M Alcott

The Bone Garden by Tess Gerittsen

The aim of the Bad Bugs Book Club is to get people interested in science, specifically microbiology, by reading books (novels) in which infectious disease forms some part of the story. We also try to associate books, where possible, with some other activity or event, to widen interest, and to broaden impact.

We hope to encourage others to join, to set up their own bookclub, suggest books and accompanying activities to us, and give feedback about the books that they have read (j.verran@mmu.ac.uk).

Our bookclub comprises both microbiologists and members of the general public. We felt that this would encourage some discussion on the science – accuracy, impact etc – as well as about the book.

Little Women by Louisa M Alcott (1868) is a very well-known story of four sisters growing up during the American Civil War. One of the sisters contracts scarlet fever, and subsequently (probably) dies of the related condition, rheumatic fever. The Bone Garden by Tess Gerritsen (2007) describes a series of gruesome murders taking place in the 1840s, related to a maternity hospital in which puerperal/childbed fever is killing women who have just given birth. The 'bad bugs' aspect in both books is the bacterium *Streptococcus pyogenes* (pyo-pus; genes – producing).

Questions

Who read both books? One book? Which? Did you see any films?

Little Women

1. Did you read the book/see the film(s)? If so, when? Have you read the book afresh? What did you think of the book/film/characters/story?
2. What do you know about the author?

3. Do you think the novel conveyed well the time in which it was set (1861)/written (1868)?
4. Since our focus is on infectious disease, what is the disease that affected the plot so dramatically? How did Beth catch it? Why was she ill for so long afterwards (6 years) – do you think it was rheumatic fever?
5. The bacterium that causes scarlet fever is *Streptoccocus pyogenes*. What are its key properties/pathogenic determinants?
6. What are the main manifestations of *S.pyogenes*?
7. What are the symptoms of scarlet fever? How important was it in the mid-nineteenth century? How did families try to prevent the spread of infection?
8. How important is *S,pyogenes* nowadays?
9. How are/were infections caused by *S.pyogenes* treated?
10. Do you think Little Women has potential for future Bad Bugs Bookclub users? Are you aware of the novel 'March' by Geraldine Brooks?

The Bone Garden

1. Did you enjoy the book? When was it set?
2. What are your opinions of the plot/setting/characters?
3. Why was there such antagonism and prejudice against 'the Irish'?
4. How were medical students trained in the mid-nineteenth century?
5. Did you spot any real-life characters?
6. What are resurrectionists? Abolitionists? Transcendentalists? Unitarians?
7. The main infection described in this book was puerperal fever /childbed fever. What is puerperal fever? What was its prevalence in the 1840s? How was it transmitted? What infection did Charles Lackaway contract when his hand was injured?
8. How was puerperal fever eventually prevented? Who first published findings on transmission and prevention?
9. Do you think that The Bone Garden is an interesting book for the Bad Bugs Bookclub?

Comparing the two novels

1. Apart from the obvious differences in writing style, plot, intended audience etc., there are several common aspects of the two novels. Did you spot any of them?
2. How different were the degrees of poverty/being poor presented in the two novels? How did fashion/clothing illustrate these differences?
3. Consider how the novels present the array of infections caused by *S.pyogenes*.
4. Do you think that this was a sensible/useful book pairing?

Joanna Verran

Bad Bugs Bookclub Reading Guide: Moloka’i by Alan Brennert

The aim of the Bad Bugs Book Club is to get people interested in science, specifically microbiology, by reading books (novels) in which infectious disease forms some part of the story. We also try to associate books, where possible, with some other activity or event, to widen interest, and to broaden impact.

We hope to encourage others to join, to set up their own bookclub, suggest books and accompanying activities to us, and give feedback about the books that they have read (j.verran@mmu.ac.uk).

Our bookclub comprises both microbiologists and members of the general public. We felt that this would encourage some discussion on the science – accuracy, impact etc. – as well as about the book.

Moloka’I by Alan Brennert (2003) describes the life of Rachel (1886 – 1970), a Hawaiian girl, who was exiled aged seven to the town of Kalaupapa on the island of Moloka’i. This island was used as a quarantined leprosy settlement for over a century.

Questions

1. Did you enjoy the book? The characters? Were there particular aspects that stood out for you? How old was Rachel when she died?
2. The book was inspired by significant historical research. Did you do any additional research?
3. What did you learn about Hawaii and its history and culture? Were/are Hawaiians particularly susceptible to disease?
4. Did you find evidence of racism? What was thought to be the origin of the disease? What does leprosy reveal in terms of prejudice/attitudes?
5. What role did religion play on the island?
6. What did you know about leprosy before you read the book?
7. How is leprosy transmitted and diagnosed?

8. How was leprosy prevented/controlled? Was isolation successful in controlling incidence/prevalence? What regulations on Molokai helped to enforce this separation? Did this remind you of any other diseases?
9. What different approaches were used in the treatment of leprosy? When and how did treatment become successful? Did the treatment have any long-term effects?
10. How did the book compare with others we have read about leprosy (the Island by Victoria Hislop and The Samurai's Garden by Gail Tsukiyama). What was the attitude of the Japanese to leprosy?
11. What is the current prevalence of leprosy? Is it useful to learn about a disease little experienced these days? Did the book cast light on any aspects of coronavirus pandemic?
12. Will you read the sequel? Do you think the book is useful in microbiology education? Do you have other questions/questions suggested in the book?

Joanna Verran

Bad Bugs Bookclub Reading Guide:

The Pull of the Stars by Emma Donoghue

The aim of the Bad Bugs Book Club is to get people interested in science, specifically microbiology, by reading books (novels) in which infectious disease forms some part of the story. We also try to associate books, where possible, with some other activity or event, to widen interest, and to broaden impact.

We hope to encourage others to join, to set up their own bookclub, suggest books and accompanying activities to us, and give feedback about the books that they have read (j.verran@mmu.ac.uk).

Our bookclub comprises both microbiologists and members of the general public. We felt that this would encourage some discussion on the science – accuracy, impact etc – as well as about the book.

The Pull of the Stars (2020) by Emma Donoghue is set in a maternity/fever ward in Dublin towards the end of the First World War, and during the 1918 influenza pandemic. It describes an intense four-day period as the nurses, patients and other staff confront childbirth, death and life outside the ward.

Questions for Discussion

1. Did you enjoy the book? Were you aware of the author previously? Did you see any comparison with her most well-known book, Room (or the one we had read previously, Frog Music)?
2. Why was the novel called The Pull of the Stars?
3. All but one of the characters were fictional. Did you think they were well drawn? Did you look up more about the one ‘real’ character? What were the romantic themes?
4. What was happening in Dublin (and Ireland) in 1918? Did any factors external to the maternity/fever ward impact on the plot?

5. Of course, the focus of the novel is very much on women. How were they affected by the mores of the time?
6. Were/are pregnant women particularly susceptible to influenza? (what about coronavirus?)
7. The women in the ward were brought together by two common factors: imminent birth and influenza. How well did you think influenza was portrayed as it affected the different characters? Who died from influenza? What was the significance of red to brown to blue to black?
8. We read the Bone Garden about women in a (Boston, Catholic) hospital in the previous century. Did you see any similarities between these two novels? Was puerperal fever/childbed fever noted at all? Was the midwifery/obstetrics explained well? What is whiteleg?
9. How was the population preventing or treating influenza?
10. How do the descriptions of the influenza pandemic sit alongside other books we have read about influenza?
11. Did the novel make you reflect on your experience of coronavirus in any way?
12. If you could ask the author a question, what would it be?

Joanna Verran

Bad Bugs Bookclub Reading Guide:

Many Different Kinds of Love by Michael Rosen, and Summer by Ali Smith

The aim of the Bad Bugs Book Club is to get people interested in science, specifically microbiology, by reading books (novels) in which infectious disease forms some part of the story. We also try to associate books, where possible, with some other activity or event, to widen interest, and to broaden impact.

We hope to encourage others to join, to set up their own bookclub, suggest books and accompanying activities to us, and give feedback about the books that they have read (j.verran@mmu.ac.uk).

Our bookclub comprises both microbiologists and members of the general public. We felt that this would encourage some discussion on the science – accuracy, impact etc – as well as about the book.

Many Different Kinds of Love by Michael Rosen (2021) describes the experiences of the poet/author (in his early 70s) during his hospitalisation, induced coma and slow recovery from COVID-19 in spring 2020. Summer by Ali Smith (2020) is a work of fiction set in the UK during February – July 2020, when the coronavirus pandemic first hit. The pandemic is part of the backdrop to the story and plays no direct part in it.

Questions for Discussion

Michael Rosen

1. Have you read/did you know of Michael Rosen previously?
2. Did you enjoy the book? What were your first/emotional responses? Did you find humour?
3. Did you enjoy the format/layout/style of the story (free verse, disjointed text, use of space)? Rosen said that the gaps help the author not tell the reader what to think (as a modernist signal, compared with, for example Enid Blyton’s ‘naughty girl’).

4. Do you think that Rosen has become a voice for COVID sufferers? What other impressions did you take from reading the book?
5. Were you moved by the author's experiences – anything particular?
6. This is not an acute illness. Did Rosen's narrative of his recovery contribute to your understanding of, for example, long COVID or other post-viral syndrome?
7. What did the various carers do? Where did they come from? Did your views of the NHS alter at all having read the book?
8. The theme of the book is love – who is the target, or the giver of this love?

Ali Smith

1. Did you enjoy the book? Have you read any other of Smith's novels? (particularly Autumn, Winter, Spring). Smith has been 'the great chronicler of our age'. Do you agree or disagree?
2. The aim of the book was to write a 'current' novel and publish it as close to the time within as possible. Do you think this aim provided something new to literature?
3. Smith won the Orwell prize for political fiction for Summer: 'a time-capsule which will prove to be essential reading for anyone seeking to understand the mood of Britain during this turbulent time'. Where was politics evident in the novel? Did anything particular stand out for you?
4. Did you think that the acknowledgement of the coronavirus pandemic in the novel was interesting/understated/important?
5. Does the immediacy of the writing and the speed of publication add to or detract from our understanding of living in the coronavirus pandemic?
6. The news is constantly 'streamed' through the novel. The timeframe is, of necessity, short – do you think it will date/has dated?
7. In terms of the non-coronavirus aspects, there were many connections between characters and events throughout the novel. Which did you spot?
8. What role did migrants and refugees have in the novel?
9. Q for author: were you conscious of time passing/events happening – when did you know how/when to stop? Were you concerned about where it (novel? Coronavirus) was going? Did you plan an arc? If yes, how?

General questions

1. The two books were very different, but we were looking for references to coronavirus. Did you see any other similarities between the two?
2. Do you think there will be covid fiction in the future? What form might it take? Do you think these two books are representative (Direct impact vs background impact)?

Joanna Verran

Bad Bugs Bookclub Reading Guide:

Hamnet by Maggie O’Farrell

The aim of the Bad Bugs Book Club is to get people interested in science, specifically microbiology, by reading books (novels) in which infectious disease forms some part of the story. We also try to associate books, where possible, with some other activity or event, to widen interest, and to broaden impact.

We hope to encourage others to join, to set up their own bookclub, suggest books and accompanying activities to us, and give feedback about the books that they have read (j.verran@mmu.ac.uk).

Our bookclub comprises both microbiologists and members of the general public. We felt that this would encourage some discussion on the science – accuracy, impact etc – as well as about the book.

Hamnet (2020) by Maggie O’Farrell is a fictional account of the death from plague of Shakespeare’s son, and the impact of this death on Hamnet’s mother (and latterly on Shakespeare himself).

Questions

1. Did you enjoy reading the book?
2. How realistic were the settings? Have you been to Stratford on Avon?
3. Do you think Hamnet was a good choice for Bad Bugs Bookclub?
4. Is the book about plague? Or what is it about?
5. One review says ‘an aspect that has gained accidental pertinence’ is the narrative of a family in the shadow of pestilence. Do you think this attention is justified? Can we learn anything about plague from the book in the current context of the coronavirus pandemic?
6. There is a lot of avoidance of ‘naming’. Why is naming so important, and what effect does omission have?

7. Is the book about Hamnet?
8. Agnes (Anne Hathaway) is somewhat ethereal, a ‘wise woman’. Did any of her remedies appear to you as potentially effective?
9. Within the book there are stories within stories. For example, are you convinced as to how Judith caught the plague? What do you know about plague transmission, and how does this story dovetail with your knowledge?
10. The movie All is True (2018) gives a different perspective to Hamnet’s death. Was plague in Stratford-on-Avon in 1598? (Does it matter?)
11. How do Shakespeare’s plays resonate within the novel?
12. There are many, many novels about plague – they could be divided into the settings of time periods of the Black Death, the Great Plague, and ‘modern times’. Are you familiar with any of these novels? Why do you think there are so many novels about the plague? Which would be most suitable for use in science education?

Joanna Verran

Bad Bugs Bookclub Reading Guide:
The Perfect Predator by Steffanie Strathdee and Thomas Patterson

The aim of the Bad Bugs Book Club is to get people interested in science, specifically microbiology, by reading books (novels) in which infectious disease forms some part of the story. We also try to associate books, where possible, with some other activity or event, to widen interest, and to broaden impact.

We hope to encourage others to join, to set up their own bookclub, suggest books and accompanying activities to us, and give feedback about the books that they have read (j.verran@mmu.ac.uk).

Our bookclub comprises both microbiologists and members of the general public. We felt that this would encourage some discussion on the science – accuracy, impact etc – as well as about the book.

The Perfect Predator (2019) is a memoir by Steffanie Strathdee and (her husband) Thomas Patterson, describing 'a scientist's race to save her husband from a deadly superbug' – using phage therapy. At that time (and still), this was an extremely innovative approach to treatment, and the efforts of the scientists and medical/clinical staff involved in Tom's treatment and care are also included in the story. The authors joined us online.

Questions

1. How was it for you as a read? Are there any differences in perspectives from 'scientists/non-scientists'? How was it for you as a science learning experience? Did you want to find out more about the author? If yes, where did you look?
2. Clinical case studies are typically written as non-emotional reports (see references below for example). In contrast, this (obviously much longer) memoir is driven by the love of Steff for her husband (supported by her knowledge and experience as scientist/'pitbull'). It has been suggested in the pedagogy literature that the personalisation of case studies assists learning –

do you think this is likely to be the case? Would this book be of interest to students – studying what?

3. Does the book represent the lives of scientists/clinicians/medics/careworkers well? How important do you think collaboration is in science?
4. The memoir includes many back stories about different microorganisms and microbiologists (see previous read Microbe Hunters by Paul du Kreif). Did you enjoy these supplementary stories (the ‘gumshoe epidemiologists’ such as the work of John Snow in a previous read The Ghost Map)? Did you find any stories particularly interesting?
5. The underlying theme of the memoir was the emergence of antimicrobial resistance (AMR), the search for alternative therapies and a route for production (see previous read A Fierce Radiance by Lauren Belfer). Why is AMR such a problem? What drug resistant bacteria have you heard of? Why did Steff pack ‘cipro’ when they went on holiday? Was this a good idea? Do you have ‘spare’ antibiotics in the house?
6. Much is made of the emergence of multi-resistant bacteria. Had you heard of *Acinetobacter baumannii*, the ‘worst bacteria [sic] on the planet’? Should you have? Do you like the term Iraqibacter? Where do you think Tom’s Iraqibacter came from? Why are hospitals such a focus for ‘nosocomial’ infections? What was your reaction to the technical terms and bacterial nomenclature?
7. Did you know about phage? Aren’t they amazing! Were the explanations clear/interesting? (We have read previously about searching for treatments - Arrowsmith by Sinclair Lewis)
8. Microbiologically speaking, were there any aspects that were impressive/irritating/incorrect/informative/fascinating?
9. Tom has several septic shock episodes. Could you see any parallels to the Michael Rosen book we read recently (Many Different Kinds of Love) describing his experience of COVID? Did you see any other points that reminded you of the covid pandemic?
10. What did Steff learn from her experiences with HIVAIDS work that helped her? Are there similarities between the HIV and the superbug pandemics? What about COVID?
11. Did you find any part particularly emotive? Spiritual?

12. The impact/outcome of Tom’s treatment has been far-reaching. Why is phage therapy especially difficult? Why might genetic engineering be necessary?
13. How does this book contribute to the aims of WAAW (World Antimicrobial Awareness Week). What particular points does it raise?
14. How do you think novels about AMR differ from those about infectious disease?

Questions for the authors

1. How are you both?
2. How easy was it to write the book, as a scientist used to writing in a passive non-emotional tone? When did you start writing? How did you decide when/where/how to include Tom’s interludes? What was the intended readership? How would the movie portray the drama?
3. How did you cope with the insurance claims/cost?
4. Were you uncomfortable with the sense of privilege?
5. How has writing the book/living the experience changed your lives?

Joanna Verran

References

Rao S et al., 2021. Critically ill patient with multidrug-resistant Acinetobacter baumannii respiratory infection successfully treated with intravenous and nebulised bacteriophage therapy. AAC (Antimicrobial Agents and Chemotherapy) doi:10.1128/AAC.00824-21

Schooley RT., 2017. Development and use of personalised bacteriophage-based therapeutic cocktails to treat a patient with a disseminated resistant Acinetobacter baumannii infection. AAC 61(10)1 – 15 doi: 10.1128/AAC00954-17

Bad Bugs Bookclub Reading Guide:

The End of Men by Christina Sweeney-Baird

The aim of the Bad Bugs Book Club is to get people interested in science, specifically microbiology, by reading books (novels) in which infectious disease forms some part of the story. We also try to associate books, where possible, with some other activity or event, to widen interest, and to broaden impact.

We hope to encourage others to join, to set up their own bookclub, suggest books and accompanying activities to us, and give feedback about the books that they have read (j.verran@mmu.ac.uk).

Our bookclub comprises both microbiologists and members of the general public. We felt that this would encourage some discussion on the science – accuracy, impact etc – as well as about the book.

The End of Men by Christina Sweeney-Baird (2020) describes how a viral pandemic that kills only males impacts on the globe – before, during and after the event. It is told through the experiences of several (up to 20) women (one man) and encompasses the years 2026 – 2031. The author joined us online.

Questions for discussion

1. The End of Men was written as speculative fiction in June 2019, foreword written in April 2020. Did you enjoy the book? Are some of the author's speculations rather too close for comfort?
2. The book was set in 2026 - 2031, but there is no mention of coronavirus, which would have happened before the beginning of the time at which the novel was set – do you think coronavirus should be mentioned as part of normality in fiction in general, since it is now an historic/globally important event?

3. Did anything resonate with you compared with the current pandemic?
4. The story is written from many different points of view (POV). Did you have a favourite/least favourite character/experience? A previous read, World War Z, was also written by survivors. Do you see similarities/differences?
5. What did you find out about the pathogen? What caused the disease? How was it transmitted? Did you think the science held up?
6. How was the disease eventually controlled? Was this plausible (perhaps in the context of what we know about COVID-19)?
7. Is this a feminist novel? Have you read any non-fiction books in this context? Catherine says: 'this must be what men used to feel like'. How did women cope in a world without most men? How were things changed after the pandemic? Were there any consequences of the pandemic that you hadn't anticipated?
8. There seem to be a few novels where a pandemic/cataclysmic event forces women to 'succeed/cope'. Do you know of any of these novels? Does this happen in fiction with men?
9. 'You always assume that people in power will know what to do'. Discuss! Who did know what to do?
10. Who has the God complex? The remaining men or the successful women?
11. Sweeney-Baird writes in a Guardian article (23 April 2021) 'people have always used fiction as a way of processing the world in times of crisis'. Do you think the Bad Bugs Bookclub enables this?

Questions for author

1. What was the process of writing the novel from the initial idea to publication?
2. How did you decide on the characters? Did you have a favourite character?
3. The stories are very human – how did you work on the global politics issues?
4. Did you have any say on the audiobook voices?
5. The book is set in the very near future. Is there anything you missed?
6. What do you think happened to the United Kingdom?
7. Would you have done anything differently now that we have had the coronavirus pandemic? What next?

Joanna Verran

Bad Bugs Bookclub short Reading Guide: Darwin's Radio by Greg Bear

The aim of the Bad Bugs Book Club is to get people interested in science, specifically microbiology, by reading books (novels) in which infectious disease forms some part of the story. We also try to associate books, where possible, with some other activity or event, to widen interest, and to broaden impact.

We hope to encourage others to join, to set up their own bookclub, suggest books and accompanying activities to us, and give feedback about the books that they have read (j.verran@mmu.ac.uk).

Our bookclub comprises both microbiologists and members of the general public. We felt that this would encourage some discussion on the science – accuracy, impact etc – as well as about the book.

Darwin's Radio by Greg Bear (1999) is a 'technothriller' describing how endogenous 'Junk' DNA (retrovirus?) begins to express, causing enormous and rapid evolutionary change in *Homo sapiens*. The impact on a variety of characters, and the actions of scientists and politicians are also addressed.

The bookclub discussed this and other similar (gendercide) novels at a shortened meeting with fewer members (because as a secondary suggested read, not all members had read it). Thus there are fewer questions than usual, although there are big topics that can derive from these questions.

Questions

1. Did you enjoy the book? Did you 'learn' from it? It was written in 1999 (Bill Cosby gets a mention) – has it dated?
2. How does microbiology fit into the plot? What other infections are caused by retrovirus? What is endogenous retrovirus?
3. The story is detailed, the technology is intricately explained, and there are many characters. Were you able to follow the science underpinning the story?

Did any other aspects stand out for you? For example politics, business, women's rights, disease prevention, management and control…

4. Women were directly affected in this novel. Do any of the issues have resonance today?
5. If the topic of 'gendercide' is a genre of fiction, are you aware of any other novels that address the topic? Do you think this is a fruitful topic, or is somewhat hackneyed?
6. Would you recommend this as a good book for our bookclub? Would it be useful for (science) student discussion?

Joanna Verran

Bad Bugs Bookclub Reading Guide:
Off Target by Eve Smith

The aim of the Bad Bugs Book Club is to get people interested in science, specifically microbiology, by reading books (novels) in which infectious disease forms some part of the story. We also try to associate books, where possible, with some other activity or event, to widen interest, and to broaden impact.

We hope to encourage others to join, to set up their own bookclub, suggest books and accompanying activities to us, and give feedback about the books that they have read (j.verran@mmu.ac.uk).

Our bookclub comprises both microbiologists and members of the general public. We felt that this would encourage some discussion on the science – accuracy, impact etc – as well as about the book.

Off Target by Eve Smith (2022) is set in a near/parallel future where genetic engineering of children has become the norm and parents are prepared to take immense risks to ensure their babies are ‘perfect’. This novel takes us into a series of dilemmas around conception, child-rearing and relationships as Susan, desperate to conceive, finds herself faced with significant moral choices. The author joined us online. A twitter discussion was also hosted.

Questions

1. What emotions did this novel stir in you?
2. Do you think Eve has drawn good portraits of the various protagonists? Who for you was the most empathetic character?
3. What do you think of Susan’s decision? Did Carmel have a persuasive role? Does Susan’s first decision affect subsequent ones? Do you/where do you think she should have stopped? Do you think that Zurel was a good name choice (would Steve have approved)?

4. How well is the science underpinning the concept of the novel explored and explained? What do you know about CRISPR, and the role of women in its discovery and implementation?
5. The book notes the development of DNA for family history, and the potential ramifications of that - revealing family secrets on the way. How acceptable to you is genetic screening/engineering/editing? How far can you go? Does the novel make you think about it? What is ‘Off Target’?
6. Ethics, morals, eugenics - where is the line? Does the humanzee exist?
7. Does the novel sufficiently address the impact on the children affected by their parents’ decisions?
8. Was it unfortunate/inevitable/timely that Eastern Europe (Ukraine/Russia etc) was brought into the plot? Do global aspects in the novel complement current issues regarding Ukraine’s invasion? Is the behaviour of different countries prescient or predictable?
9. When do you think the story was set? Can you work it out? Where are the clues?
10. Would you have liked to live in Susan’s technology-assisted world? What is your favourite example? Is there anything else you would have liked?
11. The protesting groups, role of social media, newspaper articles, adverts, all added more realism. Again, did you think this was timely? How did you feel about the violence?
12. Are there any other issues that you think might be useful for discussion? ‘A woman’s right to choose’ for example?

The author also poses questions for discussion at the end of the novel.

Questions to the author

1. Where did the idea for the book come from? How much research did you do?
2. Why is Susan’s daughter called Zurel?
3. Are you pleased with the reaction to this novel? How does it compare with your previous book?
4. How did the fact that Susan’s father wasn’t her biological father contribute to the plot?

Off Target Twitter Questions

1. 8.00 Welcome to our discussion on #OffTarget by @evecsmith. Please use #badbugsbookclub in your posts/responses, and reference the question number (1-6) every time. You can ask questions too! First (not a question) say hi and tell us why you are interested in, or have read #OffTarget.
2. 8.01 Q1 As an ongoing theme throughout the discussion, what questions would you like to ask @evecsmith and our scientists about #OffTarget? Please keep asking! #badbugsbookclub
3. 8.10. Q2 #OffTarget featured aspects of genetic engineering: good and bad. How much of this was new to you? Were you surprised about what might be possible one day? #badbugsbookclub
4. 8.20 Q3 What did you particularly like or find challenging in #OffTarget? #badbugsbookclub
5. 8.30 Q4 In #OffTarget, choices are made that have significant ethical and moral implications. Were you surprised by any of the consequences? What would you have done? #badbugsbookclub
6. 8.40 Q5 How far away do you think we are from the fictional world of #OffTarget? #badbugsbookclub
7. 8.50 Q6 #Offtarget author Eve has previously said that, in this world of questionable facts and news, she believes storytelling is more important than ever to engage people in real life issues. Do you agree?#badbugsbookclub

9.00 That’s it thank you! If you have any more questions, please post on #badbugsbookclub and we will look at them over the next 24 hours.

Joanna Verran

Bad Bugs Bookclub Reading Guide:
The Bone Code by Kathy Reichs

(and The Locked Room by Elly Griffiths)

The aim of the Bad Bugs Book Club is to get people interested in science, specifically microbiology, by reading books (novels) in which infectious disease forms some part of the story. We also try to associate books, where possible, with some other activity or event, to widen interest, and to broaden impact.

We hope to encourage others to join, to set up their own bookclub, suggest books and accompanying activities to us, and give feedback about the books that they have read (j.verran@mmu.ac.uk).

Our bookclub comprises both microbiologists and members of the general public. We felt that this would encourage some discussion on the science – accuracy, impact etc – as well as about the book.

For this meeting, two books were considered, both of which were selected from a series, to see how the coronavirus pandemic was incorporated into the narrative.

The Bone Code (2021) by Kathy Reichs is the 21st in a series focusing on forensic scientist Temperance Brennan. In this story, two bodies are washed up in a container, and Tempe links them to a previous case of hers alongside an unusual disease outbreak, in a post-pandemic USA/Canada setting.

The Locked Room (2022) by Elly Griffiths is the 14th in a series focusing on forensic archaeologist Ruth Galloway. In this story a series of suicides appear to be linked, but investigation proves difficult as the country (UK) goes into lockdown.

Questions

Who read both books? Who would read more in either series?

The Bone Code – general questions first, then to the science

1. Did you enjoy the writing style?

2. What was Tempe's job? How did she find out about science with which she was not acquainted? (how does that help the reader?)
3. How did the phrenology/death masks link to the overall plot? Did you look up Robert Noel? If yes, how did the information you found differ from that in the novel?
4. There is a lot of technical text, particularly around the post-mortems – what did you think of it? What about the more gory aspects?
5. There is a fair bit of microbiology in the narrative – did you spot bacteria, viruses and even algae?
6. What did you find out about *Capnocytophaga*? How many cases were there? How many outbreaks/clusters? What sort of reaction did the public have? Did capno spread from person to person? Did every person bitten by a dog get capno do you think? Does every dog carry capno?
7. How much mention is there of covid? Did the recent pandemic impact on the story?
8. The vaccine provides a lot of discussion points for our group. In part, we are told about all the different types of influenza vaccine – egg-based flu vaccine, cell-based flu vaccine, recombinant flu vaccine. Did you find this useful?
    a. Do we presume that it is the flu vaccine that is modified by the bioterrorists (would you say they were bioterrorists)? How often is the flu vaccine given in the US, and to whom?
    b. What type of vaccine was modified?
    c. We have read about CRISPR in our previous book (Off Target). What did you learn about CRISPR? What is the function of CRISPR in the modified vaccine? Why was the vaccine modified?
    d. Did you find the description of the mRNA vaccine useful? Was Reichs positive about this vaccine? How significant were mRNA vaccines in the 'fight against COVID'?
9. Tempe speculates that this vaccine meddling could have been associated with other disease outbreaks. What were they, and do you think they are good examples (for speculative fiction)?
10. Do you think that this book gives vaccines positive support? Does it fit with the bookclub aims? Did you look at other reviews?

The Locked Room

1. Did you enjoy reading this book? Again, what did you think of the writing style and the characters?
2. Could you see any similarities between the two novels? How successfully do these novels absorb covid into the long-term narrative of their characters?
3. Both novels are very much related to place. Would you like to visit the locations (Norfolk, Montreal, Charleston), in terms of how the authors describe them?
4. COVID plays a significant role in the behaviour of the protagonists in the Locked Room. Do you think it presents a good representation of the beginning of the pandemic in the UK (or elsewhere)?
5. How obedient are our protagonists with respect to the lockdown rules? Do you think the novel could be used to discuss different reactions to lockdown?
6. Did you like the plot? How did COVID interfere with the investigation? Do you think the book is appropriate for our bookclub aims?

Joanna Verran

Bad Bugs Bookclub Reading Guide:
The Fever Tree by Jennifer McVeigh

The aim of the Bad Bugs Book Club is to get people interested in science, specifically microbiology, by reading books (novels) in which infectious disease forms some part of the story. We also try to associate books, where possible, with some other activity or event, to widen interest, and to broaden impact.

We hope to encourage others to join, to set up their own bookclub, suggest books and accompanying activities to us, and give feedback about the books that they have read (j.verran@mmu.ac.uk).

Our bookclub comprises both microbiologists and members of the public. We felt that this would encourage some discussion on the science – accuracy, impact etc – as well as about the book.

The Fever Tree by Jennifer McVeigh (2012) is a sweeping narrative of English privilege, Victorian adventure, diamond mining history, South Africa, love, and lust! A key focus of the novel is a smallpox outbreak, which affects the lives of the protagonists, the miners and the mine owners.

Questions

1. How well for you did the book fit the bookclub remit? Did you enjoy it? Why is acacia called the fever tree?
2. What did you think of Frances' character? What do you think her mother died of? Did her father bring her up well? Did Frances being Irish have any role in the story or the nature of the characters?
3. How does Edwin Matthews compare to William Westbrook? What affects Frances' impression of them both? How does this affect Frances' views of smallpox?
4. The book provides interesting narratives about a range of topics. How does it address:

    a. racism/colonialism?
    b. The business of diamond mining?
    c. Life on the veldt/Karoo?
5. Although smallpox is fundamental to the story development, it is not mentioned until one third of the way through the novel. Why do you think it takes so long to emerge? Are any other disease mentioned in passing?
6. What do you know about smallpox vaccination? When was it first used? How successful was it? Why does it not protect Frances and her friends? Were there any antivaxxers in the novel?
7. Why is the quarantine station so important? What is the value of fumigation?
8. Why are the reasons for smallpox-denying? What is pemphigus?
9. What do you know about poxviruses?
10. 'Spillover events' occur when diseases jump from animals to humans. Are you familiar with any examples?
11. What do you know about monkeypox? How is it related to smallpox? Do you think knowledge of smallpox will help our understanding of the emerging monkeypox outbreaks?
12. Can you see any parallels between smallpox, monkeypox and the coronavirus pandemic?

Joanna Verran

Bad Bugs Bookclub Reading Guide:
State of Wonder by Ann Patchett

The aim of the Bad Bugs Book Club is to get people interested in science, specifically microbiology, by reading books (novels) in which infectious disease forms some part of the story. We also try to associate books, where possible, with some other activity or event, to widen interest, and to broaden impact.

We hope to encourage others to join, to set up their own bookclub, suggest books and accompanying activities to us, and give feedback about the books that they have read (j.verran@mmu.ac.uk).

Our bookclub comprises both microbiologists and members of the general public. We felt that this would encourage some discussion on the science – accuracy, impact etc – as well as about the book.

State of Wonder by Ann Patchett (2011) describes the journey of Marina Singh to the Amazon jungle as she searches for a research colleague who is missing, believed dead, and attempts to discover the progress of the research project being conducted there. Research is being carried out on a natural product that can extend female fertility into old age, but is also potentially useful as a malaria vaccine. Conflicting attitudes on research funding, clinical trials, ownership, intervention and many other topics are complemented by beautiful descriptions of the journey, and of changes in Marina’s ‘state’.

Questions for discussion

1. Did you enjoy the book?
2. Many of the characters were scientists. How do you think science and scientists were represented? What relationships did they have with one another, and how did this affect their behaviours? Did any of them change as the story developed?
3. Are there good and bad characters in the book?

4. Much of the novel was set in Manaus and subsequently the Amazon jungle. What are your views on the pace of Marina's journey/quest, and how did it affect the storyline?
5. The research was funded by a pharmaceutical company (Vogel). How did you view the monitoring of the funding? What did Vogel think it was funding? What was it funding?
6. What are your views on the ethics of this particular clinical trial? How well was it designed, if at all? What contribution did the Lakashi make to the trial?
7. Do you see any aspects of racism/colonialism/'going native' in the story? What was the relationship between the two indigenous tribes, and how did the researchers interact with the two tribes (Lakashi and Hummoca)?
8. What is the value of children and childbirth to the various protagonists? Swenson, Singh, the Lakashi, Hummoca, Anders, Vogel etc. How does Easter fit in?
9. What is the global burden of malaria? Why is it so difficult to control? What methods are used to control/prevent malaria?
10. Marina is endlessly pestered with insect bites. What are the risks of insect borne (eg arboviruses) in Brazil? What other microbial infections are noted in the story?
11. What is the origin of the treatment and its mode of action? How important are the interactions between these components of a unique ecosystem (what are the components?).
12. How important are fungi to man and life in general?
13. Lariam is used as a prophylactic – prevents infection. How does that differ from the vaccination that the new drug offers? Are there currently any vaccines for malaria?

Joanna Verran

Bad Bugs Bookclub Reading Guide:

All the Young Men by Ruth Coker Burks, and Love from the Pink Palace by Jill Nalder

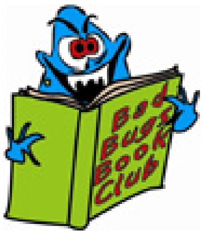

The aim of the Bad Bugs Book Club is to get people interested in science, specifically microbiology, by reading books (novels) in which infectious disease forms some part of the story. We also try to associate books, where possible, with some other activity or event, to widen interest, and to broaden impact.

We hope to encourage others to join, to set up their own bookclub, suggest books and accompanying activities to us, and give feedback about the books that they have read (j.verran@mmu.ac.uk).

Our bookclub comprises both microbiologists and members of the general public. We felt that this would encourage some discussion on the science – accuracy, impact etc – as well as about the book.

For this session (World AIDS Day), both of our chosen books described the authors' encounters with people suffering from AIDS in the 1980s. We wanted to remind ourselves about the severity of AIDS, the suffering and impact of the pandemic – in part due to the relative success of treatments resulting in fewer media stories nowadays. This disease is part of our history, and our present. It should not be forgotten or overlooked.

In All the Young Men by Ruth Coker Burks (2020), Ruth first comes across a very ill young man who has been isolated and badly treated in a hospital. After his death, she supports and befriends many extremely sick young men with AIDS until their death, treating them with love and dignity, and helps them find housing, treatment and somewhere to be buried. In Love from the Pink Palace (2022), Jill Nalder tells of her life in musical theatre, and how many of her friends are directly affected with HIV, many dying from AIDS, with other surviving as treatments arise.

Members were also recommended How to Survive a Plague by David France (2016), which is a detailed and moving narrative of the entire pandemic, focusing on

the activism of many of those people affected by AIDS, including patient engagement with scientists, and a range of political angles. This was supplementary reading – two books were already more than enough!

Questions

Before you start your discussions, perhaps find out whether members have read one or both books, and then modify the questions as appropriate.

1. What did you think of the book(s) you read? If you only read one, why did you choose it?
2. The two narratives shared many common themes. If you read both, how did they compare? What did you think of the writing style? Did the location affect the story progression?
3. What about the narrators? It has been said of Ruth, 'Erin Brockovitch meets Dallas Buyers Club'. Would you agree? Jill is a successful West End actor. How did this profession impact on her story?
4. Many of the most memorable characters were PWA (people with AIDS). How did they cope with their prognosis? Were there any similarities between the gay characters in the two books?
5. Both are recent publications (2020, 2022), yet both are set in the early-mid 1980s. Why do you think it took so long for these books to be written? What value do you see in these stories 40 years after the events they describe? Was any mention/comparison made to COVID in the texts? Can you make any comparisons – or are the two diseases too different?
6. Did you do any research on the authors? Since they are both telling their stories, what do you think the stories revealed about their characters?
7. Both books focused on the lives of the friends of the authors. How did these friendships develop and progress? Were any of the characters well known?
8. What do you know about AIDS? Progression, current treatment, prevalence, prognosis, prevention etc. Did the book add to your knowledge/perspective? Should we keep talking about AIDS? What is the epidemiology of HIV/AIDS now?
9. There were many microorganisms/diseases described in addition to HIV. How familiar are you with these different opportunist pathogens?

10. How well did the two books convey the issues surrounding AIDS at the time? Did the settings (UK/US) affect perspectives? Have prejudices changed?
11. What TV/films/documentaries/novels have you encountered related to HIV/AIDS, and how did they inform your perceptions or knowledge?

An additional question that might be added to every bookclub meeting!

12. We have been involved with covid for more than 2 years now. What other 'bad bugs' have you noticed in the media lately?

Joanna Verran

Bad Bugs Bookclub Reading Guide:

Pure by Andrew Miller

The aim of the Bad Bugs Book Club is to get people interested in science, specifically microbiology, by reading books (novels) in which infectious disease forms some part of the story. We also try to associate books, where possible, with some other activity or event, to widen interest, and to broaden impact.

We hope to encourage others to join, to set up their own bookclub, suggest books and accompanying activities to us, and give feedback about the books that they have read (j.verran@mmu.ac.uk).

Our bookclub comprises both microbiologists and members of the general public. We felt that this would encourage some discussion on the science – accuracy, impact etc – as well as about the book.

Pure by Andrew Miller (2011) describes the varied experiences of an engineer in 1785 Paris, tasked with organising the removal of hundreds/thousands of skeletons from Les Innocents cemetery.

Questions

1. Did you enjoy the book? Did you like the characters? What could you find out about the characters from their activities (reading, theatre etc)? Did you look at book reviews?
2. Did you learn anything? Did the book inspire you to find out more about the period/the cemetery? Did you enjoy the more morbid aspects of the task of cemetery clearance?
3. Why do you think the novel was called Pure?
4. What was the reason for clearing out the graveyard and the church? Have you been to that part of Paris? What has happened in that area since this excavation? Where are the bones now?

5. Why did some people not want the cemetery excavated? What was happening in Paris at the time? Are there indications of unrest/revolution?
6. The existence/clearing/excavation of cemeteries still gets interest from the public/media. Can you think of any examples?
7. It is not very easy to find anything about bad bugs here. What examples did you find? How might discussion be extended? (or is it too far to stretch?)
8. What is the role of smell/breath/air in the novel? Where did the odour come from? What about the breath of some of the protagonists?
9. Is there any microbiological value or relevance in the excavation of cemeteries? Are there any risks? In the book, is there any evidence of risk?
10. The aim of the Bad Bugs Bookclub was ‘to get people interested in science, specifically microbiology, by reading books (novels) in which infectious disease forms some part of the story. We also try to associate books, with some other activity or event, to widen interest and to broaden impact. Our bookclub comprises both microbiologists and members of the general public. We felt that this would encourage some discussion on the science – accuracy, impact etc – as well as about the book’. Do you think these aims still hold? What else do you think the bookclub has achieved (if anything)? This book, and some others (recent and older) have not been directly about microbiology, so are they of benefit to the aims of the bookclub?

Joanna Verran

Bad Bugs Bookclub Reading Guide:

The Great Stink by Clare Clark

The aim of the Bad Bugs Book Club is to get people interested in science, specifically microbiology, by reading books (novels) in which infectious disease forms some part of the story. We also try to associate books, where possible, with some other activity or event, to widen interest, and to broaden impact.

We hope to encourage others to join, to set up their own bookclub, suggest books and accompanying activities to us, and give feedback about the books that they have read (j.verran@mmu.ac.uk).

Our bookclub comprises both microbiologists and members of the general public. We felt that this would encourage some discussion on the science – accuracy, impact etc – as well as about the book.

The Great Stink by Clare Clark (2005) is set in the year 1858, where ‘the great stink’ describes the smell emanating from the Thames at the time. The novel describes how ex-soldier William May is involved in Bazalgette’s reconstruction of the sewerage system – amongst corruption, filth and murder in the underground world. The author joined us online.

Questions

1. What were your first impressions of the novel? Did it paint a good picture for you?
2. Can you see any similarities between Clark’s work and Dickens?
3. The ‘hero’ William May had some significant events in his life – the war in Crimea, sewer engineering and surveying, marriage and family life, asylum, prison boat. What did you make of his character? How well do you think his wife coped with him? Did any other characters stand out for you?
4. Were any real-life characters mentioned? What was happening in London at the time?

5. Did you do any extra research? Did you find out anything new/interesting to you? What role did the city of London itself play in the novel?
6. The role of mental health was addressed in several contexts. Is this relatively modern treatment surprising in a novel set in Victorian times?
7. How did different characters take their leisure?
8. The descriptions of the Crimea, the sewer, the asylum and the prison ship were vivid and perhaps vile? Do you think the author was able to portray the varying types of filth?
9. As with Pure our previous read, there was little direct reference to disease, although the ‘miasma’ theory of disease transmission would have found a significant role if it were true! Was this lack of infection surprising? What evidence of infectious disease did you find? Why was there little mention of cholera? How do you feel the two books compared with regards to conveying issues of odour and filth? What is ‘slop gas’?
10. How might the book be used in science education? Humanities education? Science literacy? Engineering? Is the Great Stink in the school curriculum?
11. Can you see any relevance of issues addressed in this novel that touch upon issues today?

Questions for the author

1. What was your inspiration for the book? How did you go about the research?
2. What did you want the reader to take away?
3. Did you plan a happy ending?
4. The bricks were important to the plot – why was this?
5. How did you do the research for the mental health aspects of the novel?
6. Crimea, sewer, asylum, prison boat, various ‘homes’. All with different types of filth. Did you run out of words to describe it all?
7. Since your title is the same as that of the phenomenon itself, did you fear any crossover or confusion with non-fiction works?

Joanna Verran

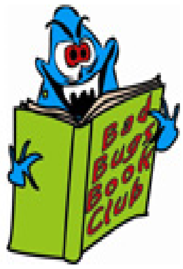

**Bad Bugs Bookclub Reading Guide:
The Doomsday Book by Connie Willis**

The aim of the Bad Bugs Book Club is to get people interested in science, specifically microbiology, by reading books (novels) in which infectious disease forms some part of the story. We also try to associate books, where possible, with some other activity or event, to widen interest, and to broaden impact.

We hope to encourage others to join, to set up their own bookclub, suggest books and accompanying activities to us, and give feedback about the books that they have read (j.verran@mmu.ac.uk).

Our bookclub comprises both microbiologists and members of the general public. We felt that this would encourage some discussion on the science – accuracy, impact etc – as well as about the book. Since May 2020 our meetings have been held online.

The Doomsday Book by Connie Willis (1992) is an award-winning science fiction novel. In the near-future, research through time-travel is being conducted in Oxford. Postgraduate student Kivrin travels back in time to the early 1300s, but an error in 'the net' causes her to arrive during the Black Death. In the future from which she is sent, Oxford also experiences an outbreak of a new influenza strain, shortly after a significant pandemic. The novel (almost 600 pages) describes how the two diseases affect human behaviour and survival in the two time periods – and the attempts to bring Kivrin back.

Questions

1. What were your impressions of the novel? Did you enjoy reading it? Would you agree that it is science fiction? For those of you who enjoy science fiction, how did this book stand in your judgement? What do you know about the author? Will you read more of her work?

2. There were a lot of characters, both in the 'present' and the past. Did you have any favourites? How did you find the character development? What was the role of Basingame? William? Colin? William's mother?
3. What about pace?
4. Did your version have an introduction by Adam Roberts? If yes, did it bring any insights?
5. The book was written in 1992. When was it set?
6. How successful was the author in looking forwards? In terms of
    a. technology,
    b. human behaviour
    c. pandemic response
    d. pandemic consequences
7. Uncannily, the author describes a recent pandemic. How did that affect or inform behaviours in the new outbreak?
8. What was the new pathogen? How was it managed/controlled? What did you learn about influenza epidemiology? Did you see any resonance with our experiences during COVID?
9. Who was patient zero in the present/the origin of the influenza outbreak?
10. How successful was the author in looking backwards?
11. Did you learn a lot about the plague?
12. What difference did Kivrin make to the outbreak in her village? How was she prepared, and how successful were the preparations? Were any incorrect assumptions made about the past by the time travelling researchers?
13. Could you find any parallels in the two eras in the book?
14. Did you spot any errors in the text – did that matter? Are we being too pedantic if we criticise? For example, cholera and influenza were noted to be prevalent during the medieval time period – where is the evidence for this?
15. Two pandemics are described in this novel. Influenza killed 20,000,000 in 1918; plague killed 50,000,000 in 1349. There have been seven cholera pandemics in the past 200 years. Covid killed at least 7,000,000. COVID was recently declared to no longer be a global health problem. Do you think that this book has value in terms of microbiology education?

Joanna Verran

Bad Bugs Bookclub Reading Guide:

Spike by Jeremy Farrar with Anjana Ahuja

The aim of the Bad Bugs Book Club is to get people interested in science, specifically microbiology, by reading books (novels) in which infectious disease forms some part of the story. We also try to associate books, where possible, with some other activity or event, to widen interest, and to broaden impact.

We hope to encourage others to join, to set up their own bookclub, suggest books and accompanying activities to us, and give feedback about the books that they have read (j.verran@mmu.ac.uk).

Our bookclub comprises both microbiologists and members of the general public. We felt that this would encourage some discussion on the science – accuracy, impact etc – as well as about the book.

Spike (2021) by Jeremy Farrar with Anjana Ahuja tells the story of Jeremy’s experiences during the beginning and first year of the COVID pandemic. As the director of the Wellcome Trust, and an infectious disease expert, he was a key participant and observer in the events taking place.

Questions

1. This book was chosen as a non-fiction example to enable us to reflect on our experience of the pandemic. Do you enjoy reading non-fiction?
2. What were your first impressions of the book?
3. ‘We are living through history’ is a quote from the book. Do you agree?
4. What do you know about the author? What role did the co-author have do you think?

5. Looking back, are there any particular aspects of the book or the events that it describes that annoy or impact on you in any positive/negative ways? Does the content reflect any of your experiences of covid?
6. Is this a book for a wide readership? How accessible/readable did you find it?
7. The book is very UK-centric. How would non-UK readers find it? Are there comparable books in other countries?
8. What other non-fiction books have you come across about the pandemic? Do you know of any other books that might have been a better/additional choice? What value might these accounts have in HE/more widely?
9. What would you ask the author(s) if you had the opportunity? Sequel?
10. Is it useful to use a non-fiction book to parallel our fiction reading experiences?

Joanna Verran

Bad Bugs Bookclub Meeting Report:

The Butchering Art by Lindsey Fitzharris

The aim of the Bad Bugs Book Club is to get people interested in science, specifically microbiology, by reading books (novels) in which infectious disease forms some part of the story. We also try to associate books, where possible, with some other activity or event, to widen interest, and to broaden impact.

We hope to encourage others to join, to set up their own bookclub, suggest books and accompanying activities to us, and give feedback about the books that they have read (j.verran@mmu.ac.uk).

Our bookclub comprises both microbiologists and members of the general public. We felt that this would encourage some discussion on the science – accuracy, impact etc – as well as about the book.

The Butchering Art by Lindsey Fitzharris (2017) is a biography of Joseph Lister, describing his ‘quest to transform the grisly world of Victorian medicine’. He used scientific methods and experiments to test his theory for prevention of contamination and infection in post-surgical wounds.

Questions for discussion

1. What were your first impressions on reading the book?
2. Was Lister someone with whom you were familiar? Did you learn anything new about him? What was your impression of his personality (did you like him?). Have you read any other books about him?
3. What was the role of women in the hospital environment in Lister’s time?
4. Can you put yourself in the era, and understand the thoughts prevalent at the time regarding infection/surgical practice/cause of infection etc? What must it have felt like?

5. How well did Lister conduct his research? Did he follow the scientific method? Was he a good scientist? (Read about Semmelweis and see how the two compare.)
6. The era in which Lister conducted his work was stellar in terms of discoveries about microbiology, hygiene etc. Several discoveries and individuals are mentioned in the book. Can you think/did you find out about any others?
   a. Louis Pasteur and the germ theory (they were mutual admirers)
   b. Florence Nightingale and the hygiene hypothesis
   c. Mary Seacole
   d. Ignacz Semmelweiss, puerperal fever
   e. Oliver Wendell Holmes, puerperal fever
   f. Edwin Chadwick, sanitaria
   g. John Snow, cholera
   h. Sara Elizabeth Ward-Roberts, nurse training
   i. Van Leeuwenhoek
7. There was much opposition to Lister's discoveries (and those of Semmelweis). What were the reasons for this opposition, and was it justified?
8. Is this type of opposition apparent today for different types of pioneering medical/surgical etc discoveries?
9. Did you notice any topics from books we have read previously? Do you have any additional recommended reading?
10. When we teach about disease transmission, we note routes of ingestion, inhalation, inoculation, direct contact (animate/inanimate - fomites), vertical transmission. Which route(s) of transmission was Lister addressing?
11. Do you think that this book could be of value in teaching medical microbiology? If yes, in what ways? What about in education generally, or for a lay public.

Joanna Verran

Bad Bugs Bookclub Reading Guide:
The Great Believers by Rebecca Makkai
and

The Way We Live Now by Susan Sontag

The aim of the Bad Bugs Book Club is to get people interested in science, specifically microbiology, by reading books (novels) in which infectious disease forms some part of the story. We also try to associate books, where possible, with some other activity or event, to widen interest, and to broaden impact.

We hope to encourage others to join, to set up their own bookclub, suggest books and accompanying activities to us, and give feedback about the books that they have read (j.verran@mmu.ac.uk).

Our bookclub comprises both microbiologists and members of the general public. We felt that this would encourage some discussion on the science – accuracy, impact etc – as well as about the book.

For World AIDS Day 2023, we read The Great Believers by Rebecca Makkai (2018), an award-winning novel that 'chronicles a mother's search for her estranged daughter against the backdrop of the AIDS crisis, and contemplates the ripples of grief affecting generations of survivors' (www.Pulitzer.org). We coupled this rather long piece (just over 500 pages) with Susan Sontag's short story (20 pages) The Way We Live Now (1986), which also focuses on the impact of HIV on a circle of friends.

The Great Believers

1. Was the book a 'good read'? Did you find out anything about the author? Do you think she did a lot of research?
2. The book is described as 'An artful novel that chronicles a mother's search for her estranged daughter against the backdrop of the AIDS crisis, and contemplates the ripples of grief affecting generations of survivors' (Pulitzer) and 'A novel of friendship and redemption in the face of tragedy' (Makkai). Would you agree?
3. The novel is written from two POV's in two time periods. Why do you think the baton was passed between those two people (and places) rather than any

other pair in the story? What are the links between the two periods? What third time period is important to the text?

4. There is a significant ensemble of characters, particularly in the earlier time period. How did you find this? Who did you/didn't you like?
5. How did AIDS impact on behaviour and relationships of the characters? What roles did testing and activism play?
6. Did you learn anything different about the disease, and how was this affected by the narrative and characters? Do you think there are more novels about AIDS that we could read as a bookclub?
7. In terms of HIVAIDS, how do you think the narrative compared with last year's books (All the Young Men by Ruth Coker Burks, and Love from the Pink Palace by Jill Nalder)? Which point of view do you prefer in terms of exploring the impact of HIV/AIDS during the early stages of the pandemic? (observer – as last year's books - or embedded, as these. NB last year's books were factual, this is not). Do you think the story is useful in the context of HIV/AIDS today? As an educational experience?
8. What role did the art have in the narrative? Did art have any relationship with disease?
9. Did you have any feel for who would survive the 1980s? What are the long-term health impacts of years on anti-HIV medication? What are the current issues affecting the incidence of HIV/AIDS? When did antiretroviral therapy begin to 'work'?
10. What did you think of Susan Sontag's short story? What do you know about Susan Sontag? How did you read it? (quickly, slowly). How many characters were in the support network? (how many in Makkai's during the 1980s period?)
11. Does the AIDS story depart from Sontag's usual output. Can you see any similarities between the two books?
12. What is this year's message for World AIDS Day?

Joanna Verran
